# Fire blight disease reactome: RNA-seq transcriptional profile of apple host plant defense responses to *Erwinia amylovora* pathogen infection

**DOI:** 10.1038/srep21600

**Published:** 2016-02-17

**Authors:** Tim Kamber, Jan P. Buchmann, Joël F. Pothier, Theo H. M. Smits, Thomas Wicker, Brion Duffy

**Affiliations:** 1 Agroscope Changins-Wädenswil ACW, Plant Protection Division, Wädenswil, Switzerland; 2 Institute of Plant Biology, University of Zurich, Zurich, Switzerland; 3 Environmental Genomics and Systems Biology Research Group, Institute of Natural Resource Sciences, Zurich University of Applied Sciences (ZHAW), Wädenswil, Switzerland

## Abstract

The molecular basis of resistance and susceptibility of host plants to fire blight, a major disease threat to pome fruit production globally, is largely unknown. RNA-sequencing data from challenged and mock-inoculated flowers were analyzed to assess the susceptible response of apple to the fire blight pathogen *Erwinia amylovora*. In presence of the pathogen 1,080 transcripts were differentially expressed at 48 h post inoculation. These included putative disease resistance, stress, pathogen related, general metabolic, and phytohormone related genes. Reads, mapped to regions on the apple genome where no genes were assigned, were used to identify potential novel genes and open reading frames. To identify transcripts specifically expressed in response to *E. amylovora*, RT-PCRs were conducted and compared to the expression patterns of the fire blight biocontrol agent *Pantoea vagans* strain C9-1, another apple pathogen *Pseudomonas syringae* pv. *papulans*, and mock inoculated apple flowers. This led to the identification of a peroxidase superfamily gene that was lower expressed in response to *E. amylovora* suggesting a potential role in the susceptibility response. Overall, this study provides the first transcriptional profile by RNA-seq of the host plant during fire blight disease and insights into the response of susceptible apple plants to *E. amylovora*.

Plants have developed an arsenal of defense responses elicited by biotic and abiotic stresses. The specific recognition of pathogen effectors (Avr) by disease resistance (R) proteins leads to the induction of a hypersensitive response (local cell death) at the infection site and inhibition or growth stop of the pathogen. These activated responses are accompanied by an induction of salicylic acid (SA) dependent signaling and expression of pathogen related proteins contributing to disease resistance. Cell wall reinforcement by callose and lignin deposition as physical barriers and phytotoxin production at the entry site of the pathogen represent a first line of defense. Defence responses against many necrotrophic pathogens are dependent on the combined accumulation and signaling of jasmonic acid and/or ethylene^1^. Beside this, jasmonic acid and ethylen also control responses to wounding and various stresses^2^,^3^.

The apple genome of the fire blight susceptible cultivar ‘Golden Delicious’ (*Malus* × *domestica* Borkh., family Rosaceae, tribe Pyreae) was recently sequenced and annotated^4^. The assembly covers about 81.3% of the genome sequence and approximately 90% of the genes. The apple genome consists of 63,541 predicted genes with an estimated size of 742.3 Mb. The genome sequence provides the basis for genetic, genomic, and transcriptomic analyses to gain insights into the apple biology.

The molecular basis of resistance and susceptibility of apple to *Erwinia amylovora,* a plant pathogenic enterobacterium that is the causative agent of the fire blight disease, is largely unknown. The variable levels of tolerance found in germplasm collections are indicative of a complex cohort of disease response genes^5^. This is further supported by several discovered quantitative trait loci that exhibit different levels of resistance^6^. Nevertheless, the recent discovery of individual genes indicates a gene-for-gene relation, similar to the interaction of RPS2 from *A*. *thaliana* and AvrRpt2 from *Pseudomonas syringae* with RIN4 as guard, for the *Malus* × *robusta* 5 – *E*. *amylovora* system^7^. However the molecular background and activation of the disease response remain relatively unclear. The infection process is accompanied by an oxidative burst in compatible and incompatible *E*. *amylovora*-host plant interaction, indicating that the bacterium might exploit this pathway to invade host plant cells^8^. The hormone level of jasmonic acid increased in the resistant cultivar Evereste compared to the susceptible MM106, whereas salicylic acid accumulates to the same extend in both conditions^9^. The lower accumulation of jasmonic acid and down-regulation of genes belonging to the jasmonate pathway might be an important step for successful infection of *Malus* spp. by *E*. *amylovora.*

*E. amylovora* is a major threat to pome fruit production globally, with further impact on ecologically important cornerstone species. The pathogen infects plants mainly through the nectarthodes of flowers^10^. Further, bacteria might get access through wounds (*e.g.*, mechanical, insect depredation, hail damage). Once in the host plant, it can spread via the vascular system throughout the whole plant. The multiplication of bacteria inside the vessels leads to their disruption and causing the fire blight typical necrotic lesions in infected tissues^11^. *E*. *amylovora* can provoke disease symptoms in fruits, shoots (“shepherd’s crook”) and rootstocks. Successful infection of susceptible host plant is dependent on secreted proteins from the bacterium by the hypersensitive response and pathogenicity (Hrp) type III secretion system (T3SS)^12^,^13^. HrpN and the disease-specific protein, DspA/E, are essential for virulence, since mutations in the encoding genes renders the pathogen less (Δ*hrpN*) or non-virulent (Δ*dspA/E*)^14^,^15^. DspA/E physically interacts with four serine/threonine receptor kinases of apple, designated as DspE-interacting proteins^16^. Beside these well characterized virulence factors, novel insights into host-specificity, evolutionary aspects, and core genes were provided by comparative genomic approaches of sequenced *E*. *amylovora* strains^17^,^18^,^19^,^20^,^21^.

Previous studies used microarray, cDNA amplified fragment length polymorphism or suppression subtractive cDNA hybridization techniques to identify genes involved in the *E*. *amylovora*–*Malus* interaction^22^,^23^,^24^. These studies gave first insights into this host-pathogen interaction, but are not reflecting the whole genome-wide transcriptional changes. In this study, we used RNA-sequencing to analyze the apple transcriptome of *E*. *amylovora* challenged flowers to gain further knowledge of the susceptible responses. The infection led to the differential expression of 1,080 transcripts at 48 h post inoculation, including genes of the jasmonic, ethylene, phenylpropanoid pathway, pathogenesis-related, and putative disease resistance genes. Additionally, previously unknown genes in the apple genome could be assigned and ORFs were detected in the genomes of *M.* × *domestica* and *Prunus persica*.

## Results

### Apple transcriptome analysis in response to *E*. *amylovora*

**Figure 1:**
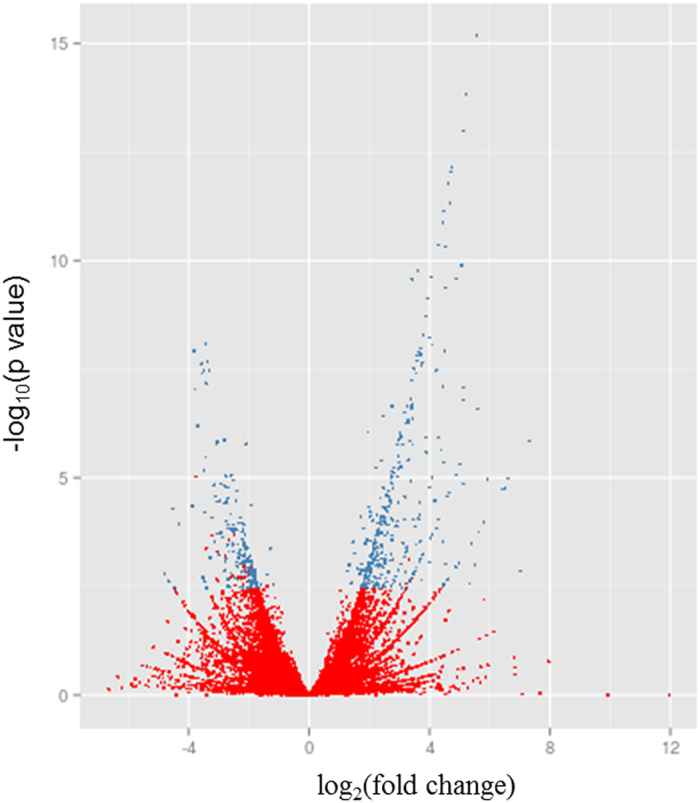
Volcano plot representing all expressed transcripts. For every transcript, the fold change of control and inoculated plant was plotted against the –log *P* value. Statistically significant differentially expressed genes, with a fold change ≥1.5 or ≤−1.5, are depicted as blue, insignificant as red dots. Of the 67,958 expressed transcripts at 48 h post inoculation 1,080 were differentially expressed (872 up- and 208 down-regulated).

**Figure 2:**
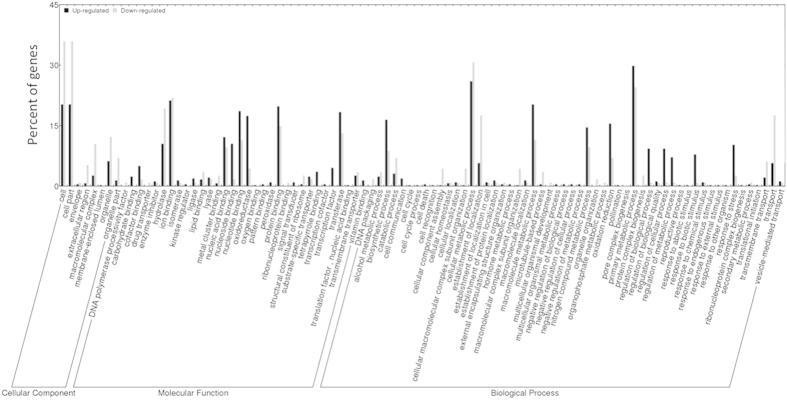
Classes of apple genes (GO terms) that were differentially expressed in *Malus* × *domestica* ‘Golden Delicious’ flowers infected with *E. amylovora* (versus mock-inoculated non-infected controls) representing the fire blight disease apple reactome.

We inoculated apple flowers to investigate the susceptible response of the primary infection court of *E*. *amylovora*. Newly opened flowers were inoculated with a bacterial suspension or mock inoculated with water and collected 48 h post inoculation. The time-point of sampling was expected to include the temporal expression of genes previously associated with the fire-blight disease, *e*.*g* PR genes, jasmonic acid biosynthesis genes^9^,^25^. Disease symptoms were not visible at the stage of sampling. Total RNA was isolated from flowers cleared from petals, rRNAs were depleted and subsequently the cDNA libraries were constructed for sequencing. A total of 3,549,589 and 12,839,290 (control, inoculated sample) 100 bp reads were obtained from sequencing. The reads were filtered from primer and polyA + sequences resulting in 1,991,992 (mock-inoculated) and 6,138,188 (pathogen inoculated) reads, of which 47.1% and 47.5% respectively could be aligned to the apple genome sequence. The reads mapped to 63,508 annotated genes and 2,064 unannotated regions in the apple genome sequence. Analysis of the mapped reads showed that the infection of *E*. *amylovora* led to significant differential expression of 1,080 apple transcripts, of which 208 are down- and 872 are up-regulated (Fig. 1; Supplementary Table 1). The differentially expressed transcripts were assigned to cellular component, biological processes and molecular function according to the gene ontologies (GO) (Fig. 2). The main biological processes include response to stimulus, pigmentation, metabolic process, localization, establishment of localization, cellular process, and biological regulation.

### Discovery of novel differentially expressed genes, open reading frames, and conserved non-coding sequences

Of the 1,080 differentially expressed transcripts, 840 showed sequence identity to annotated apple genes. However, 240 (217 upregulated and 23 downregulated) mapped to regions where previously no open reading frames (ORFs) were annotated. The sequences of these 240 unassigned transcripts were extracted from the apple genome sequence and a BLASTX analysis against *Arabidopsis thaliana* peptide database (TAIR 10) was performed resulting in 84 ORFs showing significant sequence identity to *A*. *thaliana* proteins (Supplementary Table 2). These include transcripts with similarities to 1-aminocyclopropane-1-carboxylate (ACC) synthase, glutathione transferases, disease resistance and putative pathogenesis-related genes.

The 156 sequences which did not yield a significant BLASTX hit were used in BLASTN searches against the *A*. *thaliana* and *P*. *persica* genomes to identify possible conserved non-coding sequences (CNS) or novel ORFs. None had significant DNA sequence identity with *A*. *thaliana*, while 47 sequences showed significant DNA sequence identity to the (more closely related) *P*. *persica*. These were screened for DNA alignments in apple and *P*. *persica* sequences for base mismatches that are separated from each other by multiples of three. In protein coding sequences, the third position of the codon is more likely to differ, due to the degeneration of the genetic code. We identified six sequence alignments that were significantly enriched (*P* < 0.05) for such mismatch spacing. We conclude that these six sequences represent previously unknown ORFs specific to the *Malus*/*Prunus* lineage (Table 1). The remaining 41 transcripts with no significant DNA sequence similarity between *P*. *persica* and *M.* x *domestica* showed no protein coding capacity and were classified as putative CNS. The remaining 109 transcripts have no orthologs in *P*. *persica* and represent specific *M.* x *domestica* transcripts.

### Differentially expressed genes

Genes coding for proteins with functions generally related to response to biotic and abiotic stimuli including glutathione S-transferases (*e.g.*, GSTU8, GSTU19), cytochromes (mainly P450), NAC domain containing proteins (*e.g.*, NAC083, NAC002), and ubiquitin- hydrolases and proteases (*e.g.*, BRIZ1, UBQ12, UBQ13), showed differential expression. The first three classes of genes are all upregulated, whereas the ubiquitin-related class contains up- and down-regulated genes. Both groups of genes identified as gluthatione S-transferases and cytochromes include three novel transcripts (respectively GSTU7, GSTU8, GSTU19 and CYP72A9, CYP72A14, CYP76C2).

The identified photosynthesis-related genes encoding light harvesting complexes (*e.g.*, LHCB2.2, LHCB4.2) and chlorophyll A/B binding proteins (*i.e.*, CAB1, ELIP1) are all downregulated. Processes affected during the infection of apple blossoms by *E*. *amylovora* include defense responses to bacteria and fungi reflected by the differential expression of potential disease resistance, leucine-rich repeat, phytohormones and chitinase genes. Marker genes for hormones jasmonic acid and ethylene biosynthesis and signaling pathway are upregulated. In addition to the probable ethylene biosynthesis genes 1-aminocyclopropane-1-carboxylate (ACO/ACC) and ethylene-forming enzyme (ACO), genes involved in ethylene perception and signaling are induced, *e.g.*, ethylene responsive element binding factors (ERF-), ethylene sensor (ETR2), and ethylene response factors (ERF). One of the central genes in methyl jasmonate biosynthesis, jasmonic acid carboxyl methyltransferase (JMT), is not differentially expressed at the sampling time point; however, genes potentially involved in jasmonic acid signaling were induced (*i.e.*, JAZ2, MYC2/JIN1). Transcripts of JAR1 described as responsible for conversion of jasmonate to its active isoleucine form were either not detected or only at low, insignificant levels in the control and in the challenged datasets. Genes involved in the phenylpropanoid and flavonoid biosynthesis were induced indicated by the expression of the phenylalanine ammonia lyase (PAL), chalcone isomerase (CHI) and various chalcone and stilbene synthases (CHS).

Transcripts potentially involved in either direct or indirect perception and signaling of pathogens and wounding are differentially expressed (*e.g.*, LOX2, RBOHD, MAPKKK4/19, NHL genes). Although we analyzed a susceptible host-pathogen interaction we detected putative disease resistance genes of the TIR-NBS-LRR and CC-NBS-LRR classes, receptor like kinases, WRKY transcription factors (*e.g.*, WRKY33, WRKY40) and pathogenesis-related genes (*e.g.*, PR1, PR4, PR5, PR8). All WRKY transcription factors, most putative resistance genes, and PR genes show higher transcript abundance in the infected than in the control plants.

We identified five genes encoding for leucine-rich repeat (LRR) family proteins that were differentially expressed in the *E*. *amylovora* inoculated samples. One of these LRR family proteins encoding gene, MDP0000207774, is identical to MxdRLP1-1 at amino acid level and a second, MDP0000392201 is largely similar but differs on the C terminus by being 60 amino acids longer in sequence. The third LRR family protein, MDP0000315498, is distantly related to RLP1 alleles. Based on the amino acid sequence, MDP0000281307 is not related to RLP1s. MDP0000303781 is annotated as a LRR family protein but analysis of the amino acid sequence indicates that LRR are absent from the gene product and therefore is not considered to belong to LRR-proteins.

### Reverse transcription PCR (RT-PCR)

**Figure 3:**
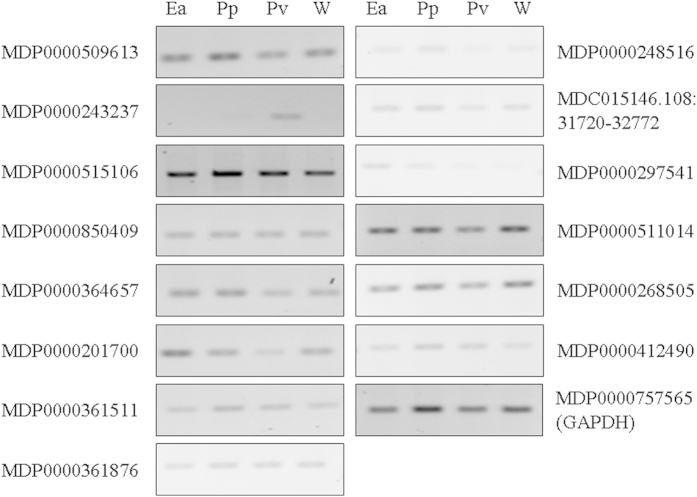
Expression patterns of selected genes in response to *E*. *amylovora* (Ea), *P*. *syringae* pv. *papulans* (Pp), *P*. *vagans* (Pv) and water (W) inoculation. Gene annotations are shown to the left and right of corresponding gel panels. The same cDNA template was used for the different primer sets with the housekeeping gene glyceraldehyde-3-phosphate-dehydrogenase (GAPDH) as a control.

In order to assess if the transcriptional changes observed in the RNA-seq data were specific apple responses to *E*. *amylovora* infection, or common responses to bacteria, RT-PCRs were performed. *E*. *amylovora* strain CFBP 1430 and *P*. *syringae* pv. *papulans* strain FAW 388–01 were chosen to detect potential differences in apple response to pathogenic bacteria, *P*. *vagans* strain C9–1 for its influence as a biocontrol strain. Genes expressed in all bacteria-inoculated flowers indicated a general reaction of the host towards these bacteria or stress responses. Water-inoculated flowers were used as control group. The flowers were inoculated with the bacterial strains or water and collected 48 h post inoculation for RNA extraction and subsequent cDNA reverse transcription. The RT-PCR was performed for 29 targets selected from the differentially expressed transcripts (Table 2) of the RNA-seq data. The targets were randomly selected and included 17 downregulated and 12 upregulated transcripts. Amplicons were obtained for 14 targets (Fig. 3) with varying band intensities for some of the genes tested in response to the different treatments. The gene MDP0000243237 was only expressed in the *P*. *syringae* pv. *papulans* strain FAW 388–01 and *P*. *vagans* strain C9–1 inoculated samples. The gene is annotated as coding for a peroxidase superfamily protein. The RNA-seq data indicated that this gene was upregulated in the *E*. *amylovora* inoculated compared to the mock*-*inoculated samples. The gene had a FPKM value of 0 in the mock and a FPKM value of 4.93 in the *E*. *amylovora* samples. The abundance of the transcripts might be too low to be detected by RT-PCR.

### Discovery of novel potential genes, ORFs, and CDS

Similar analysis, as described above, was repeated for all unassigned transcripts (non- significant differential expression) of the transcriptome and yielded 749 BLASTX hits (Supplementary Table 3). In total 1,088 transcripts that mapped to the apple genome did not correspond to annotated genes. Neither did they show sequence identity at the protein level to *A*. *thaliana* proteins. To identify possible novel ORFs or CDS, we used these 1,088 sequences for BLASTN searches against the closely related *P*. *persica*. In total, 269 ORFs showed significant DNA sequence identity (*E*-value < 10E–10) between *M.* × *domestica* and *P*. *persica*. Using the spacing between DNA mismatches as the determinative criteria (see above), 25 novel putative ORFs were identified (Table 1). We propose that these represent protein-encoding genes that are specific to the *M.* × *domestica*/*P*. *persica* lineage. The other 244 transcripts were considered to be potential CDS and the remaining 819 transcripts without DNA sequence identity to be specific to *M*. × *domestica*.

## Discussion

The fire blight disease caused by *E*. *amylovora* is a global invasive threat for apple and pear production. RNA-seq technology offers a novel tool for transcriptional profiling and to discover previously undetected genes in the target genome. The recent publication of the apple genome sequence^4^ enabled us to study the transcriptional changes in apple blossom elicited by *E*. *amylovora* in its genetic background. RNA-seq is advantageous to other methods (*e.g.*, microarray) used to analyze transcriptomes. It generates a higher nucleotide resolution, quantifies low abundant transcripts, avoids hybridization issues, and allows a non-biased survey of genes, as it enables the detection of novel ORFs beyond the selected targets for microarray. In this study, RNA-sequencing was applied to investigate the susceptible response of blossoms of the economically important apple cultivar ‘Golden Delicious’. The analysis of the susceptible response network is important to evaluate and select true resistance genes from wild species and optimize their potential^26^. The genes discussed further in more detail are summarized in Table 3.

The transcriptome analysis not only led to the discovery of pathways and differentially expressed genes in response to *E*. *amylovora*, but also revealed novel genes and putative ORFs in apple and *P*. *persica*. The BLASTX analysis of all unassigned transcripts of the apple transcriptome against the *A. thaliana* proteins yielded potential novel genes in the *M.* × *domestica* genome^4^, but did not cover all transcripts. *A*. *thaliana* is commonly used as reference to annotate novel plant genomes, but might be insufficient to annotate all genes of distantly related plant species. Therefore we applied a comparative BLASTN approach, using the closely related species *P*. *persica* genome as reference^27^, to identify potential new ORFs. This method was more efficient by using the *P*. *persica* compared to *A. thaliana* genome and led to the identification of ORFs specific to the *Malus*/*Prunus* lineage.

Our results show that genes involved in jasmonic acid responses were differentially expressed after 48 h post inoculation. It was recently reported that jasmonic acid levels increased to much higher extend in apple leaves in the resistant cultivar ‘Evereste’ compared to the susceptible MM106^9^. Treatment of the susceptible plants with methyl-jasmonate prior inoculation rendered them more resistant to *E*. *amylovora* infection. Accordingly, we found that the jasmonic acid biosynthesis genes *JMT* and *JAR1* were not differentially expressed in ‘Golden Delicious’ flowers 48 h post inoculation. However, we identified genes (*JAZ2*, *JAZ10*, *MYC2*/*JIN1*) involved in jasmonic acid signalling that are significantly up-regulated. In *A*. *thaliana JAZ2*, *JAZ10*, and *MYC2*/*JIN1* are essential modulators of the jasmonate signaling pathway. JAZ proteins were identified as negative regulators of jasmonate signaling^28^. The transcription factor MYC2/JIN1 represses defense response against necrotrophic pathogens and activates systemic responses to wounding^29^. If the differential expression pattern of *JAZ2, JAZ10*, *MYC2*/*JIN1* in the infected cultivar ‘Golden Delicious’ leads to differing jasmonic acid accumulation and signaling needs to be investigated.

Ethylene-biosynthesis, and -responsive genes were significantly induced. The ethylene-responsive genes include previously detected genes in *E*. *amylovora* infected apple plants^24^. The up-regulation of these genes suggests either a direct response to *E*. *amylovora* invasion and/or infection or a disease induced wounding/stress response. Ethylene biosynthesis silenced apple fruits were shown to be more susceptible to the fungal pathogen *Botrytis cinerea*^30^. In contrast to that led the suppression of ethylene production in *E*. *amylovora* infiltrated leaves to a significant reduction in lesion development in resistant and susceptible apple varieties^31^. Additionally, ethylene production and accumulation of reactive oxygen species (ROS) in primary lesions coincided. This led to the assumption that ethylene signaling in *E*. *amylovora* induced hypersensitive response (HR) cell death might act in combination with ROS in both the susceptible and resistant apple hosts. Tomato plants impaired in ethylene perception or synthesis in response to *Xanthomonas campestris* pv. *vesicator*ia, *Pseudomonas syringae* pv. *tomato* and *Fusarium oxysporum* f. sp. *lycopersici* showed a significant reduction in disease symptoms compared to wild-type plants^32^. Furthermore, ethylene perception was shown to be critical for systemic spread of the fungal pathogen in host-plants. Further research on the impact of ethylene signaling and perception on systemic spread and disease development in the *E*. *amylovora* pathosystem is needed to specify their exact function.

The diverse branches of the phenylpropanoid can lead to the production of compounds with antimicrobial properties, antioxidant protectants and structural components^33^. The impact of *E*. *amylovora* infection on the phenylpropanoid–flavonoid pathway was demonstrated in apple and pear and proposed to contribute to the defense mechanism^34^,^35^. Additionally, the alteration of the flavonoid metabolism by the growth-regulator prohexadione-calcium redirects the flavonoid biosynthesis, beside other polyphenols, to form compounds inhibitory to *E*. *amylovora*^36^,^37^,^38^. A gene silencing approach in apple to mimic the inhibitory effect led to the accumulation of flavonones, but did not result in an accumulation of inhibitory compounds and thus did not reduce susceptibility of the plants^39^. The observed up-regulation of PAL was in agreement with previous studies on the transcriptional responses of apple to *E*. *amylovora* infection^22^,^24^,^40^. Transient prevention of transcription of CHS genes by *E*. *amylovora* was proposed to occur at 15 h and 24 h after infection in susceptible apple genotypes^40^. A down-regulation of CHS genes was identified in apple flowers at 24 h post incoculation^24^. In our RNA-seq data all identified CHS genes were up-regulated at sampling point. The induction of genes of the phenylpropanoid pathway (*e.g.*, PAL, CHS) indicates secondary metabolite production, in the susceptible interaction of *E*. *amylovora* and *M*. × *domestica* cultivar ‘Golden Delicious’. The timing, quantity and the nature of the produced compounds might be crucial to develop an inhibitory effect. Nevertheless, the identified differentially expressed genes of the phenylpropanoid pathway in the present work provide additional targets to improve apple resistance to fire blight.

The identification of two biphenyl synthases in our data indicates the production of phytoalexins in *M*. × *domestica* cultivar ‘Golden Delicious’ flowers. The precursors of phytoalexins in apple are formed by biphenyl synthases and their respective genes were differentially expressed in fire blight infected apple plants^41^. Additionally, these phytoalexins were shown to accumulate in the transition zone (healthy-necrotic) of a susceptible apple cultivar^42^. These phytoalexins showed *in vitro* inhibitory effects against *E*. *amylovora* at elevated levels, however *in planta* the disease progress was not stopped but might been delayed. Alternatively, a resistance mechanism might exist that prevents the accumulation of phytoalexins in the bacterial cells, as proposed for interactions between apple rootstocks and *E*. *amylovora*^43^. Fruits and leaves of the cultivar ‘Golden Delicious’ produce glycosides of phytoalexins, which might develop their toxicity after being released from the plant cell vacuoles^44^. It is currently not known if a *de novo* synthesis of phytoalexins in the cultivar ‘Golden Delicious’ challenged with *E*. *amylovora* occurs.

Genes encoding LRR and TIR-NBS were detected in previous transcriptome analyses^22^,^24^. Recently, five genes encoding for leucine-rich repeat, receptor-like proteins were identified as putative fire blight resistance genes (*MxdRLP1-1* to *MxdRLP1-5*)^45^. The allele *MxdRLP1-2* was identified only in resistant cultivar *M*. × *robusta* 5, whereas the others were detected in resistant and susceptible *Malus* varieties. Genes encoding leucine-rich repeat (LRR) family proteins were detected in our study. The gene MDP0000207774 is identical to *MxdRLP1-1* and MDP0000392201 is highly similar to it on amino acid level except for the C-terminus. Both this leucine-rich repeat (LRR) family proteins had lower transcript abundance in the fire blight inoculated apple blossoms compared to the control. The *MxdRLP1* alleles are candidate resistance genes; therefore genes encoding LRR family proteins might be direct or indirect targets in the *E*. *amylovora* – *M.* × *domestica* ‘Golden Delicious’ interaction in order to modulate host defense responses. Beside these resistance genes candidates, additional searches for known candidates did not lead to their identification in our dataset^46^,^47^.

The induction of pathogenesis-related proteins has been reported in various plant-pathogen interactions during the infection process by viruses, fungi and bacteria^48^,^49^. Direct involvement in resistance to fungal infection was demonstrated for *PR1*, *PR4*, and *PR5* genes from apple and prune^50^,^51^,^52^. Differential expression of PR genes were detected in previous studies^22^,^24^. A specific up-regulation of *PR2*, *PR5*, and *PR8* genes was shown for susceptible apple genotypes in response to *E*. *amylovora,* an induction of *PR1* genes was not observed using Northern hybridization or RT-PCR^25^,^40^. Accordingly to these findings we found an up-regulation of *PR5* (thaumatin-related) genes in our RNA-seq data. However, the *PR8* gene identified was down-regulated and *PR2* was not detected in the RNA-seq data. The gene expression of additional *PR8* and *PR2* genes was under the significance level used to evaluate our data. The identification of induced genes by Northern hybridization might cover all genes of the PR multi-gene families, and is hence not applicable to detect expression changes of single genes. Further we identified in the apple transcriptome that *PR1-like*, *PR4*, *PR6, PR13* and *PR14* genes significantly differentially expressed. The *PR1-like*, *PR4*, and *PR6* had a higher transcript abundance whereas *PR13*, *PR14* and a plant defensin family gene (PDF2.2) showed lower in the inoculated compared to the mock treated flowers. The high expressed genes might be a general response to pathogen infection or alternatively the expression of these genes is delayed in the *M.* × *domestica* ‘Golden Delicious’ interaction. The exact function in the susceptible response of the lower expressed pathogenesis related protein genes needs further evaluation.

The observed down-regulation of photosynthesis genes is in accordance with observations in the *E*. *amylovora* - apple interaction as wells as in other pathosystems^22^,^23^,^53^,^54^. The photosystems are a potential source of ROS that can induce defense responses and a HR in incompatible interaction limiting pathogen growth. The opposing case occurs in compatible interactions between *E*. *amylovora* and host plants, the pathogen seems to exploit ROS production to provoke cell death to invade plant tissues^8^.

The RT-PCR revealed that MDP0000243237 was only expressed in the *P*. *syringae* pv. *papulans* strain FAW 388–01 and *P*. *vagans* strain C9–1 inoculated samples. In the RNA-seq data this gene was not expressed in the mock (FPKM value was 0) and only at a low level in the *E*. *amylovora* (FPKM value was 4.93) inoculated sample, which led to the assignment that the gene is significantly differentially expressed. This low level of expression might be under the detection limit of RT-PCR for this gene in the *E*. *amylovora* inoculated samples. Nevertheless, MDP0000243237 (annotated as coding for a peroxidase superfamily protein) is expressed in apple flowers in response to *P*. *syringae* pv. *papulans* strain FAW 388–01 and *P*. *vagans* strain C9–1 inoculation. Peroxidases are involved in diverse biological functions, including cell wall reinforcement, generation and detoxification of reactive oxygen species, stress and defense responses^55^,^56^. Until now it has not been determined in which of these processes the peroxidase gene MDP0000243237 is involved. The down-regulation at early time-points of two peroxidase genes in *E*. *amylovora* inoculated leaves in a resistant apple genotype suggested an association with resistance^45^. Up-regulation of peroxidase genes at early time-points of infection, followed by a down-regulation at later ones, was demonstrated in the interaction of *E*. *amylovora* with susceptible hosts^22^,^45^. The identified peroxidase gene in our data was assigned to be up-regulated, nevertheless the gene was lower expressed in *E*. *amylovora* inoculated samples. Since gene expression was induced in response to the other bacteria used, this indicated that *E*. *amylovora* either modulates general stress response or that the gene has a potential role in the susceptibility response of apple blossoms during the infection process.

Results of RT-PCR indicate that specific apple responses to *E*. *amylovora* CFPB 1430 can be detected by comparing the gene expression patterns of the plant inoculated with different bacteria. This approach could be extended to whole transcriptome analysis in order to identify candidate genes conferring resistance or susceptibility of different apple varieties. This is advantageous to the comparison of cultivars with different genetic background by reducing the number of genes to a common set.

The analysis of the compatible host-pathogen interaction of *E. amylovora* and *M*. × *domestica* using RNA-seq gives a first full-view of the responses in its genetic background. The genes, novel ORFs and pathways identified in this study will be used to further characterize the susceptible as well as the resistance mechanisms in *M*.* × domestica* to *E. amylovora*. Further characterization will reveal their exact function in the response and their potential application as markers for susceptibility. Further, the function and contribution to susceptibility of the differentially expressed novel ORFs, as they were previously undetected in the apple genome sequence, need to be clarified. Our data provides evidence for genes and corresponding proteins that potentially are targets for modulation of host-defense responses by *E. amylovora*. Future studies will determine if these candidates are direct or indirect targets of *E. amylovora* and their connection to the susceptibility of *M*. × *domestica* in response to the pathogen.

## Methods

### Bacterial strains, media and growth conditions

The fire blight pathogen *E*. *amylovora* strain CFBP 1430^17^, the biocontrol strain *Pantoea vagans* strain C9–1^57^ and the causal agent of blister spot, *Pseudomonas syringae* pv. *papulans* strain FAW 388–01, were used for flower inoculations. The strains were plated on King’s B medium (KB) from glycerol stocks kept at −86 °C. Incubation temperature for all strains was 28 °C. Inoculum and flower population sizes were determined on nutrient sucrose agar plates.

### Flower inoculation

All freshly opened flowers of two years old ‘Golden Delicious’ plants (three per bacterial strain), stored previously in a 4 °C cold room, were used for inoculation. The flowers were inoculated with 10 μl bacterial suspension (OD_600_ = 0.1, indicates approximately ~10^8^ cfu ml^−1^) containing one of the above mentioned strains or were mock inoculated with water. The suspension was directly applied to the hypanthium with a pipette, not touching nor damaging plant organs. The flowering trees were kept at 20 °C day and 18 °C night with a relative humidity of 80%. Flowers were collected 48 h post inoculation. The flowers were cut with scissors, removing most of the peduncles. The petals were removed and the remainder parts were flash frozen in liquid nitrogen and kept at −86 °C until RNA extraction.

### RNA extraction

Flowers were ground in liquid nitrogen using RNAse-free pestles and mortars (incubated at 200 °C overnight). Total RNA from flowers was isolated using the innuPREP Plant RNA Kit (Analytik Jena, Germany) according to the manufacturer’s instructions. Total RNA was treated with DNase I (Thermo Scientific AG, Reinach, BL, Switzerland) and PCR controls were performed to confirm absence of contaminating DNA. Quality and concentration of the RNA samples were determined using the Bioanalyzer 2100 (Agilent Technologies, Germany). Samples were pooled to the required amount of 10 μg for RNA-sequencing.

### RNA-sequencing

The cDNA libraries were prepared by Vertis Biotechnologie AG (Freising, Germany) and sequenced on an Illumina HiSeq 2000 machine. From the total RNA samples polyA + RNA was isolated, treated with tobacco acid pyrophosphatase (TAP), then a RNA oligonucleotide carrying the T7 RNA promoter sequence (5′-TAATACGACTCACTATAGGGAGA-3′) was ligated to the RNAs. First-strand cDNA synthesis with oligo(dT)-linker primer was performed and PCR amplified. The full-length cDNAs were ultrasound fragmented, end-repaired and purified using the Agencourt AMPure XP kit (Beckman Coulter Genomics). TruSeq sequencing adapters were ligated to the cDNA fragments and PCR-amplified to about 20–30 ng/μl.

### Data analysis

The RNA-seq reads were deposited at ENA and can be accessed from ArrayExpress database under accession number E-MTAB-4110 (http://www.ebi.ac.uk/arrayexpress/experiments/E-MTAB-4110/). The RNA-seq reads were mapped to the apple genome^4^ using TopHat version 2.0.0^58^ with Bowtie version 0.12.7^58^. Analysis of differential expression levels was performed using Cufflinks version 1.3.0^59^. Gene expression levels were normalized using the fragments per kilobase of exon per million mapped reads (FPKM) report values. To evaluate gene expression, four housekeeping genes coding for elongation factor 1 alpha subunit (MDP0000297959), importin alpha Isoform 9 (MDP0000126113), actin (MDP0000126113), and glyceraldehyde-3-phosphate dehydrogenase (MDP0000757565) were analyzed for significant differential expression. None of these genes was significantly differentially expressed, meeting the requirements for further transcriptome analysis. Genes were considered as up- or down-regulated, when their fold change was ≥1.5 or ≤−1.5, and they were significant at *P* < 0.005. The underlying genomic sequences of read contigs that mapped to regions without assigned genes were extracted from the apple genome for further analysis. These sequences were used for TBLASTX searches against the *A. thaliana* protein database 10 (TAIR10, http://www.arabidopsis.org) and BLASTN searches against the *P*. *persica* genome sequence version 1 (https://www.rosaceae.org) to cover previously undetected coding sequences and to screen for novel ORFs. GO annotations were retrieved from http://www.rosaceae.org and used as input to create figures with the software WEGO (http://wego.genomics.org.cn/cgi-bin/wego/index.pl)^60^.

### Construction of cDNA libraries and RT-PCR

Plant RNA for RT-PCR was isolated as described above and the RevertAid H Minus First Strand cDNA Synthesis Kit (Thermo Scientific, Switzerland) was used to construct cDNA. Aliquots of 1 μg of RNA were reverse transcribed with random hexamers according to the manufacturer’s instructions. Primer pairs were designed from the gene sequences retrieved from https://www.rosaceae.org and span intron/exon boundaries where possible. Primer sequences and PCR conditions are listed in Table 4.

## Additional Information

**How to cite this article**: Kamber, T. *et al.* Fire blight disease reactome: RNA-seq transcriptional profile of apple host plant defense responses to *Erwinia amylovora* pathogen infection. *Sci. Rep.* **6**, 21600; doi: 10.1038/srep21600 (2016).

## Supplementary Material

Supplementary Dataset 1

## Figures and Tables

**Table 1:** Novel open reading frames identified in the apple genome sequence^a^.

Chromosome	Contig^b^	Position	Length (bp)	Significantly differentially expressed
9	MDC003508.304	714–802	88	Yes
10	MDC004050.111	2972–3698	726	Yes
2	MDC006793.328	2502–2994	492	Yes
2	MDC006793.328	4674–5465	791	Yes
13	MDC017405.92	18343–18890	547	Yes
5	MDC019296.227	19299–19915	616	Yes
17	MDC000413.352	3148–3647	499	No
14	MDC002798.542	1811–2138	327	No
14	MDC003592.155	55–665	610	No
6	MDC005235.134	1103–1716	613	No
17	MDC005383.384	11599–12036	437	No
13	MDC005832.257	6368–6874	506	No
5	MDC009600.163	10216–10538	322	No
3	MDC009649.545	17049–17355	306	No
13	MDC010328.213	17735–18163	428	No
3	MDC010474.345	1721–2322	601	No
15	MDC010692.100	15414–15558	144	No
3	MDC010926.52	6566–6802	236	No
1	MDC011638.223	6587–6937	350	No
8	MDC012232.278	12728–13210	482	No
11	MDC012594.480	397–686	289	No
17	MDC013437.191	6984–7656	672	No
12	MDC014100.85	297–668	371	No
1	MDC016529.125	25569–25918	349	No
16	MDC017428.71	19752–19979	227	No
10	MDC017676.268	21261–21536	275	No
unanchored	MDC018139.149	19551–19623	72	No
15	MDC018302.132	703–1163	460	No
1	MDC020690.260	20149–20524	375	No
13	MDC038224.11	9262–9560	298	No
2	MDC041335.7	2001–2706	705	No

^a^Velasco *et al.* (2010).

^b^GenBank: ACYM00000000.1.

**Table 2:** Descriptions of RT-PCR targets.

Targets^a^	M. × domestica chromosome	A. thaliana accessions	A. thaliana chromosome	Gene name(s)	Gene description
MDC015146.108: 31720–32772	9	AT2G04780.2	2	FLA7	Fasciclin–like arabinogalactan–protein 7 (Fla7)
MDP0000201700	5	AT5G59845.1	5		Gibberellin-regulated family protein
MDP0000243237	14	AT5G17820.1	5		Peroxidase superfamily protein
MDP0000248516	11	AT5G09530.1	5		Hydroxyproline-rich glycoprotein family protein
MDP0000268505	5	AT1G29980.1	1		Protein of unknown function, DUF642
MDP0000297541	15	AT4G10810.1	4		
MDP0000361511	17	AT3G22142.1	3		Bifunctional inhibitor/lipid-transfer protein/seed storage 2S albumin superfamily protein
MDP0000361876	9	AT4G03210.1	4	XTH9	Xyloglucan endotransglucosylase/hydrolase 9
MDP0000364657	11				
MDP0000412490	4	AT1G56430.1	1	ATNAS4, NAS4	Nicotianamine synthase 4
MDP0000509613	5	AT5G54500.1	5	FQR1	Flavodoxin-like quinone reductase 1
MDP0000511014	12				
MDP0000515106	1	AT5G51970.1	5		GroES-like zinc-binding alcohol dehydrogenase family protein
MDP0000850409	5				
MDP0000757565	13	AT1G13440.1	1	GAPC-2, GAPC2	Glyceraldehyde-3-phosphate dehydrogenase

The housekeeping gene GAPC2 was used as internal control.

^a^*M*. × *domestica* accessions.

**Table 3:** Summary table of selected differentially expressed *M. × domestica* ‘Golden delicious’ genes challenged with *E. amylovora*.

Description	*M*. × *domestica accessions*	Name	Expression	*A*. *thaliana* accessions	Gene description
Jasmonate	MDP0000241358	JAZ2, TIFY10B	Up	AT1G74950.1	TIFY domain/Divergent CCT motif family protein
	MDP0000242554	JAI1, JIN1, MYC2	Up	AT1G32640.1	Basic helix-loop-helix (bHLH) DNA-binding family protein
		JAZ10, JAS1, TIFY9	Up	AT5G13220.4	Jasmonate-zim-domain protein 10
Ethylene	MDP0000805422	ERF-1	Up	AT4G17500.1	Ethylene responsive element binding factor 1
	MDP0000679280	RAP2.3, ATEBP, EBP	Up	AT3G16770.1	Ethylene-responsive element binding protein
	MDP0000689946	ERF2	Up	AT5G47220.1	Ethylene responsive element binding factor 2
	MDP0000251295	EFE, ACO4, EAT1	Up	AT1G05010.1	Ethylene-forming enzyme
		ACS6	Up	AT4G11280.1	1-aminocyclopropane-1-carboxylic acid (acc) synthase 6
		ACS6	Up	AT4G11280.1	1-aminocyclopropane-1-carboxylic acid (acc) synthase 6
	MDP0000127134	ERF1	Up	AT3G23240.1	Ethylene response factor 1
	MDP0000167207	ERF1	Up	AT3G23240.1	Ethylene response factor 1
	MDP0000920189	ETR2	Up	AT3G23150.1	Signal transduction histidine kinase, hybrid-type, ethylene sensor
Phenylpropanoid	MDP0000388769	PAL1	Up	AT2G37040.1	PHE ammonia lyase 1
	MDP0000168735	CHS, TT4	Up	AT5G13930.1	Chalcone and stilbene synthase family protein
	MDP0000716308	CHS, TT4	Up	AT5G13930.1	Chalcone and stilbene synthase family protein
	MDP0000287919	CHS, TT4	Up	AT5G13930.1	Chalcone and stilbene synthase family protein
		CHS, TT4	Up	AT5G13930.1	Chalcone and stilbene synthase family protein
	MDP0000302905	CHS, TT4	Up	AT5G13930.1	Chalcone and stilbene synthase family protein
	MDP0000208899	CHS, TT4	Up	AT5G13930.1	Chalcone and stilbene synthase family protein
	MDP0000134791		Down	AT5G05270.1	Chalcone-flavanone isomerase family protein
	MDP0000523487	TT5, A11, CFI	Up	AT3G55120.1	Chalcone-flavanone isomerase family protein
Pathogenesis-related	MDP0000348327		Down	AT1G12663.1	Pathogenesis-related plant thionin (PR-13) family protein
			Up	AT4G33720.1	Cysteine-rich secretory protein, Antigen 5, and PR 1 superfamily
	MDP0000246775		Up	AT1G75800.1	Pathogenesis-related thaumatin (PR-5) superfamily protein
	MDP0000552328		Up	AT1G75800.1	Pathogenesis-related thaumatin (PR-5) superfamily protein
	MDP0000218699		Up	AT1G75800.1	Pathogenesis-related thaumatin (PR-5) superfamily protein
	MDP0000788170		Up	AT1G78780.2	Pathogenesis-related family protein
	MDP0000782085	PR4, HEL, PR-4	Up	AT3G04720.1	Pathogenesis-related 4
			Up	AT4G33720.1	Cysteine-rich secretory proteins, Antigen 5, and PR1 superfamily
	MDP0000362305	LCR69, PDF2.2	Down	AT2G02100.1	Low-molecular-weight cysteine-rich 69, plant defensin family
	MDP0000132621		Up	AT5G43580.1	Belongs to the PR-6 proteinase inhibitor family
	MDP0000694597	LTP3	Down	AT5G59320.1	Lipid transfer protein 3. Predicted to encode a PR-14 family protein
	MDP0000661371	LTP3	Down	AT5G59320.1	Lipid transfer protein 3. Predicted to encode a PR-14 family protein
	MDP0000304369	LTP3	Down	AT5G59320.1	Lipid transfer protein 3. Predicted to encode a PR-14 family protein
	MDP0000280265	CHIA	Down	AT5G24090.1	Chitinase A. Predicted to encode a PR-8 protein
Leucine-rich repeat	MDP0000315498	DRT100	Down	AT3G12610.1	Leucine-rich repeat (LRR) family protein
	MDP0000392201		Down	AT3G20820.1	Leucine-rich repeat (LRR) family protein
	MDP0000207774		Down	AT3G20820.1	Leucine-rich repeat (LRR) family protein
	MDP0000281307		Up	AT5G07910.1	Leucine-rich repeat (LRR) family protein
	MDP0000303781		Up	AT1G15740.1	Leucine-rich repeat (LRR) family protein
			Up	AT5G66900.1	Disease resistance protein (CC-NBS-LRR class) family
	MDP0000171644		Up	AT5G66900.1	Disease resistance protein (CC-NBS-LRR class) family
	MDP0000287351		Up	AT5G36930.2	Disease resistance protein (TIR-NBS-LRR class) family
	MDP0000222184		Up	AT5G36930.2	Disease resistance protein (TIR-NBS-LRR class) family
			Up	AT5G45230.1	Disease resistance protein (TIR-NBS-LRR class) family
	MDP0000178868		Down	AT5G17680.1	Disease resistance protein (TIR-NBS-LRR class) family
	MDP0000685425		Up	AT5G17680.1	Disease resistance protein (TIR-NBS-LRR class) family
	MDP0000146255		Up	AT3G14470.1	NB-ARC domain-containing disease resistance protein
	MDP0000182716		Up	AT3G14470.1	NB-ARC domain-containing disease resistance protein
Photosynthesis	MDP0000708928	LHCB2.2, LHCB2	Down	AT2G05070.1	Photosystem II light harvesting complex gene 2.2
	MDP0000757636	LHCB4.2	Down	AT3G08940.2	Light harvesting complex photosystem II
	MDP0000656154	LHB1B1, LHCB1.4	Down	AT2G34430.1	Light-harvesting chlorophyll-protein complex II subunit B1
	MDP0000417927	LHB1B1, LHCB1.4	Down	AT2G34430.1	Light-harvesting chlorophyll-protein complex II subunit B1
	MDP0000417929	LHB1B1, LHCB1.4	Down	AT2G34430.1	Light-harvesting chlorophyll-protein complex II subunit B1
	MDP0000601491	LHB1B1, LHCB1.4	Down	AT2G34430.1	Light-harvesting chlorophyll-protein complex II subunit B1
	MDP0000235175	LHB1B1, LHCB1.4	Down	AT2G34430.1	Light-harvesting chlorophyll-protein complex II subunit B1

**Table 4:** RT-PCR primers designed in this study.

Target^a^	Chromosome	Forward primer sequence (5′–3′)	Reverse primer sequence (5′–3′)	Amplicon size (bp)	Annealing temperature (°C)
MDP0000509613	5	GACAAGAGACTACCCCCTTG	AGCAATTTCTAGTTCTAGATCAG	193	53
MDP0000243237	14	CCTTTTGTAGTGGCTGACCT	CGAGTATCAATCCTAACAAAGC	167	53
MDP0000515106	1	TTCCTTGGCAAATCAGATTG	AGCAAAGGCTTCTTCCACCT	640	53
MDP0000850409	5	CTACTCACGGAGATGGGAA	GAACGTGAATGTTGGGTTG	196	53
MDP0000364657	11	GAGGCAATGAAGATGAACC	CACATACAACTCCATCAACAA	241	53
MDP0000201700	5	CCCTCGCTACTTCACCATA	CCTTGTCGTTCTTCTTGTCC	180	53
MDP0000361511	17	GATGGTGGAGGCAGTACAA	TACCCAACTCTCTGGAGAGTT	297	53
MDP0000361876	9	GCTTTCGATCATTTCACCTA	ACCGTCCGATGACATATAGA	177	53
MDP0000248516	11	GGAGACTTCCTTGCCTCAT	AGCTTTGGTACATTTTCGG	321	53
MDC015146.108: 31720–32772	9	TCTGACCATCCTGAGGTAAG	GAAAGACAGTGCCTTCACAG	225	53
MDP0000297541	15	GTCGAGCGGCTCATCACA	GTCGTTACAATCATCGCCGT	148	53
MDP0000511014	12	CCTTAGATCTCAGAAATTCAACCA	AGGCAACGGAGGCAATGT	239	53
MDP0000268505	5	CTGATAGGCCCAAAGACAAC	GTGGCACTGCGTAATGGTA	189	53
MDP0000412490	4	CACCCTCTCGACCATCTC	CCAATGTGTCGATGTCATAGTT	211	53
MDP0000757565	13	ATTGGTGACAGCAGGTCAA	AGAGGGTGGATGCTACGTG	148	53
