# Supplementary material for: Fire blight disease reactome: RNA-seq transcriptional profile of apple host plant defense responses to Erwinia amylovora pathogen infection
Source: Sci Rep. 2016 Feb 17;6:21600. doi: 10.1038/srep21600 (PMC4756370; doi:10.1038/srep21600)
Supplement: Supplementary Dataset 1 [file srep21600-s1.pdf]

Supplementary Material

belonging to:

Fire blight disease reactome: RNA-seq transcriptional profile of apple host plant defense responses to *Erwinia amylovora* pathogen infection

Tim Kamber, Jan P. Buchmann, Joël F. Pothier, Theo H.M. Smits, Thomas Wicker & Brion Duffy

Supplemental table 1: Descriptions of the significantly differentially expressed *M.* x *domestica* transcripts in response to fire blight infection.

Supplemental table 2: BLASTx results of the significantly differentially expressed novel ORFs in *M.* x *domestica* in response to fire blight infection.

Supplemental table 3: BLASTx results of all expressed (non-significant) novel ORFs in *M.* x *domestica*.

Supplementary Table 1 **Descriptions of the significantly differentially expressed *M* . x *domestica* transcripts in response to fire blight infection.**

M. × domestica **accession**	***M* . × *domestica*** **locus**	**log_2(fold_change)**	***A . thaliana*** **accession**	**Name**	**Description**
MDP0000756448	MDC000011.378:16806-16970	1.79769e+308	AT5G46630.2		Clathrin adaptor complexes medium subunit family protein
MDP0000268505	MDC000020.209:8435-13377	-4.53718	AT1G29980.1		Protein of unknown function, DUF642
0	MDC000127.652:4251-4492	1.79769e+308	AT4G33720.1		CAP (Cysteine-rich secretory proteins, Antigen 5, and Pathogenesis-related 1 protein) superfamily protein
MDP0000163276	MDC000130.277:7155-7601	2.19171	AT5G43380.1	TOPP6	type one serine/threonine protein phosphatase 6
MDP0000659818	MDC000144.408:251-3627	1.79769e+308	AT2G39200.1	ATMLO12,MLO12	Seven transmembrane MLO family protein
MDP0000417927	MDC000149.326:14929-15724	-1.93472	AT2G34430.1	LHB1B1,LHCB1.4	light-harvesting chlorophyll-protein complex II subunit B1
MDP0000417929	MDC000149.326:17749-18547	-1.87154	AT2G34430.1	LHB1B1,LHCB1.4	light-harvesting chlorophyll-protein complex II subunit B1
MDP0000601491	MDC000149.357:271-1069	-1.87058	AT2G34430.1	LHB1B1,LHCB1.4	light-harvesting chlorophyll-protein complex II subunit B1
MDP0000235175	MDC000149.357:3094-3889	-1.80257	AT2G34430.1	LHB1B1,LHCB1.4	light-harvesting chlorophyll-protein complex II subunit B1
MDP0000265831	MDC000164.370:18851-32386	2.71647	AT5G41770.1		crooked neck protein, putative / cell cycle protein, putative
MDP0000161380	MDC000178.398:6450-13590	1.79769e+308	AT5G19485.1		transferases;nucleotidyltransferases
MDP0000256732	MDC000211.300:6961-7241	1.79769e+308	AT2G18290.1	APC10	anaphase promoting complex 10
MDP0000255157	MDC000270.326:10081-10388	1.79769e+308	AT4G21430.1	B160	Zinc finger, RING-type;Transcription factor jumonji/aspartyl beta-hydroxylase
0	MDC000319.348:8176-8367	-2.48801			
MDP0000265100	MDC000357.185:21423-36365	2.72442	AT5G23760.1		Copper transport protein family
MDP0000264101	MDC000368.238:2725-8711	1.79769e+308	AT4G05410.1	YAO	Transducin/WD40 repeat-like superfamily protein
0	MDC000416.247:30134-30295	1.79769e+308			
0	MDC000446.497:2613-3451	2.36105	AT3G19615.1		unknown protein
0	MDC000455.144:3026-3468	3.21257	AT4G24620.1	PGI1, PGI	phosphoglucose isomerase 1
MDP0000323652	MDC000503.259:1794-2875	2.4077			
MDP0000263664	MDC000567.258:7588-8550	-2.52714	AT4G25040.1		Uncharacterised protein family (UPF0497)
MDP0000639167	MDC000600.439:7351-10850	1.79769e+308	AT2G45220.1		Plant invertase/pectin methylesterase inhibitor superfamily
MDP0000134666	MDC000618.395:5589-5886	1.79769e+308	AT1G62930.1		Tetratricopeptide repeat (TPR)-like superfamily protein
MDP0000146621	MDC000636.590:1152-4139	1.79769e+308	AT5G20080.1		FAD/NAD(P)-binding oxidoreductase
MDP0000413403	MDC000636.613:20700-20894	1.79769e+308	AT1G80160.1		Lactoylglutathione lyase / glyoxalase I family protein
MDP0000256199	MDC000662.220:27328-27666	1.79769e+308	AT3G63440.1	ATCKX6,ATCKX7,CKX6	cytokinin oxidase/dehydrogenase 6
MDP0000268130	MDC000695.201:7077-7269	1.79769e+308	AT1G09660.1		RNA-binding KH domain-containing protein
MDP0000262910	MDC000703.490:1546-15062	1.79769e+308	AT3G23940.1		dehydratase family
MDP0000235454	MDC000704.27:983-1106	1.79769e+308	AT5G47720.2		Thiolase family protein
0	MDC000706.497:11518-11660	1.79769e+308			
MDP0000903417	MDC000706.499:3489-5017	1.79769e+308	AT1G61560.1	ATMLO6,MLO6	Seven transmembrane MLO family protein
MDP0000119183	MDC000707.178:9077-13361	7.01306	AT5G13830.1		FtsJ-like methyltransferase family protein
MDP0000757070	MDC000711.381:8991-9419	-2.27797	AT5G38760.1		Late embryogenesis abundant protein (LEA) family protein
MDP0000119262	MDC000769.371:651-2301	1.70216	AT3G26430.1		GDSL-like Lipase/Acylhydrolase superfamily protein
MDP0000256865	MDC000791.288:5104-7135	1.79769e+308	AT4G10490.1		2-oxoglutarate (2OG) and Fe(II)-dependent oxygenase superfamily protein
MDP0000265644	MDC000810.342:26666-27910	1.79769e+308	AT1G52800.1		2-oxoglutarate (2OG) and Fe(II)-dependent oxygenase superfamily protein
MDP0000264592	MDC000841.228:2232-8160	4.28011	AT5G20230.1	ATBCB,BCB,SAG14	blue-copper-binding protein
MDP0000268271	MDC000866.255:10198-10455	1.79769e+308	AT1G31930.1	XLG3	extra-large GTP-binding protein 3
MDP0000264034	MDC000886.330:32493-36293	1.79769e+308	AT4G03180.1		
MDP0000503940	MDC000913.258:27076-29046	2.37597	AT3G11180.1		2-oxoglutarate (2OG) and Fe(II)-dependent oxygenase superfamily protein
MDP0000361449	MDC000929.194:1888-3055	-3.06288	AT3G16520.3	UGT88A1	UDP-glucosyl transferase 88A1
MDP0000052862	MDC000929.235:4940-6389	-3.0507	AT3G16520.3	UGT88A1	UDP-glucosyl transferase 88A1
MDP0000846313	MDC001071.282:19208-19513	1.79769e+308	AT3G10760.1		Homeodomain-like superfamily protein
MDP0000134250	MDC001204.784:1228-4995	-1.79053	AT1G53280.1		Class I glutamine amidotransferase-like superfamily protein
MDP0000803538	MDC001240.396:9691-9990	1.79769e+308	AT3G03940.1		Protein kinase family protein
MDP0000159558	MDC001261.116:13665-20797	1.79769e+308	AT4G32160.1		Phox (PX) domain-containing protein
MDP0000265913	MDC001262.249:1127-4650	1.95271	AT5G47770.1	FPS1	farnesyl diphosphate synthase 1
MDP0000445218	MDC001273.205:845-1730	1.79769e+308	AT1G02680.1	TAF13	TBP-associated factor 13
MDP0000235782	MDC001276.321:32854-33398	1.79769e+308	AT4G18160.1	ATKCO6,ATTPK3,KCO6,TPK3	Ca2+ activated outward rectifying K+ channel 6
MDP0000119600	MDC001284.344:1341-1621	1.79769e+308	AT2G45760.1	BAL,BAP2	BON association protein 2
0	MDC001441.272:13692-14163	1.79769e+308	AT5G43920.1		transducin family protein / WD-40 repeat family protein
0	MDC001471.424:93507-93619	1.79769e+308	AT2G29260.1		NAD(P)-binding Rossmann-fold superfamily protein
MDP0000527355	MDC001500.115:2484-4397	-1.88554	AT1G05850.1	ATCTL1,CTL1,ELP,ELP1,ERH2	Chitinase family protein
MDP0000706016	MDC001647.167:31621-31880	1.79769e+308	AT1G32360.1		Zinc finger (CCCH-type) family protein
MDP0000498524	MDC001659.508:3688-13621	1.79769e+308	AT1G22380.1	AtUGT85A3,UGT85A3	UDP-glucosyl transferase 85A3
0	MDC001671.165:2805-2973	1.79769e+308	AT1G78380.1	ATGSTU19, GST8, GSTU19	glutathione S-transferase TAU 19
MDP0000496027	MDC001715.358:1470-1791	1.79769e+308	AT4G36220.1	CYP84A1,FAH1	ferulic acid 5-hydroxylase 1
MDP0000268417	MDC001733.221:3341-3606	1.79769e+308	AT3G59200.1		F-box/RNI-like superfamily protein
MDP0000163727	MDC001753.378:36916-37587	1.79769e+308	AT2G15780.1		Cupredoxin superfamily protein
MDP0000159000	MDC001757.664:1294-3548	3.69176	AT3G11180.2		2-oxoglutarate (2OG) and Fe(II)-dependent oxygenase superfamily protein
MDP0000576922	MDC001757.678:208-6183	4.517	AT2G36690.1		2-oxoglutarate (2OG) and Fe(II)-dependent oxygenase superfamily protein
MDP0000340981	MDC001781.94:3875-6197	1.79769e+308	AT1G28120.1		
MDP0000877084	MDC001787.334:3408-3753	1.79769e+308			
MDP0000265806	MDC001831.292:8893-9169	2.3957			
MDP0000588582	MDC001839.110:1393-3069	-2.34333	AT1G68220.1		Protein of unknown function (DUF1218)
MDP0000264961	MDC001845.296:622-3441	1.79769e+308	AT5G20885.1		RING/U-box superfamily protein
MDP0000533638	MDC001848.381:1843-2323	3.75248	AT1G24020.1	MLP423	MLP-like protein 423
MDP0000831518	MDC001848.391:1325-1805	5.13328	AT1G24020.1	MLP423	MLP-like protein 423
MDP0000831519	MDC001848.391:5087-5567	4.99847	AT1G24020.1	MLP423	MLP-like protein 423
0	MDC001867.380:3629-3727	1.79769e+308			
0	MDC001900.112:16993-17583	-2.04989			
0	MDC001938.226:2272-2436	1.79769e+308			
MDP0000607969	MDC001963.417:459-4217	2.78368	AT4G27250.1		NAD(P)-binding Rossmann-fold superfamily protein
0	MDC001963.417:4816-4925	1.79769e+308	AT4G27250.2		NAD(P)-binding Rossmann-fold superfamily protein
MDP0000025413	MDC001972.273:3026-9072	2.43751	AT5G27450.1	MK,MVK	mevalonate kinase
MDP0000844719	MDC001977.912:3360-3668	1.79769e+308			
MDP0000267282	MDC001999.548:8598-8811	1.79769e+308	AT1G80400.1		RING/U-box superfamily protein
MDP0000452572	MDC002001.729:20293-31469	1.71884	AT3G53990.1		Adenine nucleotide alpha hydrolases-like superfamily protein
0	MDC002052.246:9559-9831	1.79769e+308			
MDP0000829170	MDC002080.143:3807-5483	1.79769e+308	AT5G19440.1		NAD(P)-binding Rossmann-fold superfamily protein
MDP0000873573	MDC002124.224:10772-12083	3.23587	AT5G51970.1		GroES-like zinc-binding alcohol dehydrogenase family protein
MDP0000515106	MDC002124.224:5558-6883	2.49012	AT5G51970.1		GroES-like zinc-binding alcohol dehydrogenase family protein
MDP0000707567	MDC002124.226:5571-6882	2.42596	AT5G51970.1		GroES-like zinc-binding alcohol dehydrogenase family protein
MDP0000120175	MDC002136.624:799-1408	1.79769e+308	AT1G68760.1	ATNUDT1,ATNUDX1,NUDX1	nudix hydrolase 1
0	MDC002136.660:2651-2671	4.09695			
0	MDC002149.751:1345-1407	1.79769e+308			
0	MDC002149.751:2007-2430	2.48927			
0	MDC002218.355:6778-7176	1.79769e+308			
0	MDC002270.126:3680-3863	1.79769e+308			
MDP0000163984	MDC002285.447:6979-7069	1.79769e+308	AT4G18530.1		Protein of unknown function (DUF707)
MDP0000157186	MDC002285.478:30943-31155	1.79769e+308	AT4G16444.1		
MDP0000266136	MDC002304.132:819-8300	-2.53248	AT1G14220.1		Ribonuclease T2 family protein
MDP0000457389	MDC002306.103:1840-2063	1.79769e+308	AT5G58350.1	WNK4,ZIK2	with no lysine (K) kinase 4
0	MDC002322.251:26803-27928	4.61002	AT4G11280.1	ACS6, ATACS6	1-aminocyclopropane-1-carboxylic acid (acc) synthase 6
MDP0000837051	MDC002335.203:3094-5164	1.79769e+308	AT1G50430.1	7RED,DWF5,LE,PA,ST7R	Ergosterol biosynthesis ERG4/ERG24 family
0	MDC002385.280:8975-9237	1.79769e+308			
MDP0000165382	MDC002388.638:6966-12223	1.79769e+308	AT2G41770.1		Protein of unknown function (DUF288)
MDP0000268597	MDC002412.301:3820-15392	1.79769e+308	AT5G27640.1	ATEIF3B-1,ATTIF3B1,EIF3B,EII	translation initiation factor 3B1
MDP0000164286	MDC002413.411:10891-11713	-2.76725	AT5G14920.1		Gibberellin-regulated family protein
MDP0000269551	MDC002413.411:4634-4664	1.79769e+308	AT5G14920.1		Gibberellin-regulated family protein
0	MDC002431.300:11772-11965	1.79769e+308	AT5G19130.2		GPI transamidase component family protein / Gaa1-like family protein
MDP0000236390	MDC002446.247:7797-8476	1.79769e+308	AT3G05950.1		RmlC-like cupins superfamily protein
MDP0000833077	MDC002505.371:15860-16380	1.79769e+308	AT1G63700.1	EMB71,MAPKKK4,YDA	Protein kinase superfamily protein
MDP0000897416	MDC002555.575:20823-21885	1.80749			
MDP0000062070	MDC002587.522:8090-8266	1.79769e+308	AT5G04620.2	ATBIOF,BIOF	biotin F
MDP0000525437	MDC002618.102:55-788	1.79769e+308	AT1G20760.1		Calcium-binding EF hand family protein
MDP0000265800	MDC002672.379:8552-21592	1.79769e+308	AT2G22300.1	CAMTA3,SR1	signal responsive 1
MDP0000840722	MDC002693.97:512-884	1.79769e+308	AT1G24020.1	MLP423	MLP-like protein 423
MDP0000236536	MDC002765.351:2478-6149	2.51354	AT3G25150.2		Nuclear transport factor 2 (NTF2) family protein with RNA binding (RRM-RBD-RNP motifs) domain
MDP0000155698	MDC002771.180:9590-12097	-2.80764	AT4G25150.1		HAD superfamily, subfamily IIIB acid phosphatase
MDP0000699497	MDC002791.491:4020-6869	-1.84137			
0	MDC002799.293:2023-2423	2.25333	AT2G29330.1	TRI	tropinone reductase
MDP0000361511	MDC002827.323:10419-11049	-3.81975	AT3G22142.1		Bifunctional inhibitor/lipid-transfer protein/seed storage 2S albumin superfamily protein
MDP0000788170	MDC002892.343:26056-27195	3.94434	AT1G78780.2		pathogenesis-related family protein
MDP0000236618	MDC002931.100:11631-11924	1.79769e+308	AT3G12345.1		
MDP0000173465	MDC002957.519:16454-16916	2.88122	AT4G38240.1	CGL,CGL1,GNTI	alpha-1,3-mannosyl-glycoprotein beta-1,2-N-acetylglucosaminyltransferase, putative
MDP0000885511	MDC002975.216:22992-23382	2.46881	AT3G56710.1	SIB1	sigma factor binding protein 1
MDP0000167437	MDC003038.458:3206-3383	1.79769e+308	AT2G33580.1		Protein kinase superfamily protein
MDP0000120758	MDC003093.180:517-1215	2.722	AT4G25370.1		Double Clp-N motif protein
MDP0000146334	MDC003164.614:3761-6028	-2.65594	AT2G45960.2	ATHH2,PIP1;2,PIP1B,TMP-A	plasma membrane intrinsic protein 1B
MDP0000274905	MDC003165.207:2450-6779	1.79769e+308	AT4G20000.1		VQ motif-containing protein
MDP0000120819	MDC003190.148:129-814	1.79769e+308	AT2G36690.1		2-oxoglutarate (2OG) and Fe(II)-dependent oxygenase superfamily protein
0	MDC003190.186:1345-1510	1.79769e+308			
0	MDC003236.290:5639-6048	-2.12497			
MDP0000637694	MDC003261.485:10664-15893	1.8661	AT1G48320.1		Thioesterase superfamily protein
0	MDC003306.225:3010-3406	-1.81213	AT2G16850.1	PIP3B, PIP2;8	plasma membrane intrinsic protein 2;8
MDP0000155343	MDC003320.570:27284-27737	-2.50676	AT4G14890.1		2Fe-2S ferredoxin-like superfamily protein
MDP0000233708	MDC003334.452:3099-5117	3.43165			
MDP0000276241	MDC003359.104:24766-32452	1.79769e+308	AT3G43860.1	AtGH9A4,GH9A4	glycosyl hydrolase 9A4
MDP0000270966	MDC003384.296:5308-10574	1.79769e+308	AT5G44280.1	ATRING1A,RING1A	RING 1A
MDP0000146255	MDC003415.452:19197-19538	2.55638	AT3G14470.1		NB-ARC domain-containing disease resistance protein
MDP0000272795	MDC003436.247:8739-9967	3.41978	AT5G66170.3	STR18	sulfurtransferase 18
MDP0000134791	MDC003451.570:18763-21683	-2.29196	AT5G05270.1		Chalcone-flavanone isomerase family protein
0	MDC003469.231:3820-4226	1.79769e+308			
MDP0000427722	MDC003504.134:67401-67998	-2.42477	AT1G24020.1	MLP423	MLP-like protein 423
MDP0000593502	MDC003507.409:7615-7888	1.79769e+308	AT1G07880.2	ATMPK13	Protein kinase superfamily protein
0	MDC003508.304:714-802	1.79769e+308			
MDP0000271527	MDC003560.581:10762-10811	1.79769e+308	AT3G26040.1		HXXXD-type acyl-transferase family protein
0	MDC003571.122:29326-29962	3.67188			
MDP0000121058	MDC003620.800:295-3822	1.79769e+308	AT3G60640.1	ATG8G	Ubiquitin-like superfamily protein
MDP0000277042	MDC003638.527:2878-5280	1.79769e+308			

MDP0000170501	MDC003661.185:59137-59369	1.79769e+308	AT1G03370.1		C2 calcium/lipid-binding and GRAM domain containing protein
MDP0000680997	MDC003670.313:733-3535	1.79769e+308	AT1G77120.1	ADH,ADH1,ATADH,ATADH1	alcohol dehydrogenase 1
MDP0000147293	MDC003688.191:1671-3161	2.53723	AT4G34100.1		RING/U-box superfamily protein
0	MDC003716.306:46958-47066	1.79769e+308	AT1G03260.1		SNARE associated Golgi protein family
0	MDC003741.386:2317-2600	1.79769e+308			
MDP0000391319	MDC003758.191:289-1601	3.01485	AT5G04550.1		Protein of unknown function (DUF668)
0	MDC003761.240:3013-3454	2.52991			
MDP0000276506	MDC003761.240:7197-17791	4.45347			
MDP0000441166	MDC003788.290:5474-5635	1.79769e+308	AT2G38800.1		Plant calmodulin-binding protein-related
MDP0000276278	MDC003807.231:14012-14233	1.79769e+308	AT4G28500.1	ANAC073,NAC073,SND2	NAC domain containing protein 73
MDP0000787808	MDC003810.272:15817-16803	1.79769e+308	AT4G12350.1	AtMYB42,MYB42	myb domain protein 42
MDP0000273012	MDC003865.562:8097-8515	1.79769e+308	AT3G54040.1		PAR1 protein
MDP0000167303	MDC003919.355:3369-5166	-1.74607	AT1G01780.1		GATA type zinc finger transcription factor family protein
MDP0000171644	MDC003966.297:5407-5482	1.79769e+308	AT5G66900.1		Disease resistance protein (CC-NBS-LRR class) family
MDP0000237113	MDC004009.559:4179-15094	1.79769e+308	AT3G18290.1	BTS,EMB2454	zinc finger protein-related
MDP0000854767	MDC004013.428:929-2727	1.79769e+308	AT3G12270.1	ATPRMT3,PRMT3	protein arginine methyltransferase 3
0	MDC004047.206:4467-4494	1.79769e+308			
MDP0000830926	MDC004049.516:1567-2569	2.10525	AT5G16080.1	AtCXE17,CXE17	carboxyesterase 17
0	MDC004050.111:2972-3698	2.97995			
0	MDC004050.111:8000-8340	1.79769e+308			
MDP0000169311	MDC004086.357:2616-11039	1.79769e+308	AT3G45140.1	ATLOX2,LOX2	lipoxygenase 2
MDP0000135837	MDC004095.241:944-5617	1.60314	AT1G60140.1	ATTPS10,TPS10	trehalose phosphate synthase
0	MDC004097.232:8798-9675	3.40094	AT2G46150.1		Late embryogenesis abundant (LEA) hydroxyproline-rich glycoprotein family
0	MDC004133.336:8038-8299	1.79769e+308			
MDP0000564592	MDC004154.351:39-6072	1.79769e+308	AT5G13180.1	ANAC083,NAC083,VNI2	NAC domain containing protein 83
MDP0000487384	MDC004165.256:2099-4204	-1.8127	AT5G23860.1	TUB8	tubulin beta 8
MDP0000155233	MDC004175.403:24292-27127	-2.1379	AT1G75060.1		
MDP0000951190	MDC004175.403:6190-7715	1.79769e+308	AT5G42610.1		Protein of unknown function (DUF607)
MDP0000147913	MDC004193.226:2939-4271	1.79769e+308	AT4G10490.1		2-oxoglutarate (2OG) and Fe(II)-dependent oxygenase superfamily protein
MDP0000168767	MDC004225.334:10573-10850	1.79769e+308			
MDP0000174018	MDC004233.457:20199-20679	2.2613	AT1G24020.1	MLP423	MLP-like protein 423
MDP0000864747	MDC004233.457:6800-7280	5.22546	AT1G24020.1	MLP423	MLP-like protein 423
MDP0000864748	MDC004233.457:9098-9578	4.01462	AT1G24020.1	MLP423	MLP-like protein 423
MDP0000175004	MDC004259.286:9836-10513	1.79769e+308			
MDP0000167080	MDC004293.550:16904-17262	1.79769e+308	AT1G14610.1	TWN2,VALRS	valyl-tRNA synthetase / valine--tRNA ligase (VALRS)
MDP0000167088	MDC004293.550:69667-71109	1.79769e+308	AT5G51970.1		GroES-like zinc-binding alcohol dehydrogenase family protein
MDP0000913358	MDC004324.263:15880-16063	1.79769e+308	AT5G67630.1		P-loop containing nucleoside triphosphate hydrolases superfamily protein
MDP0000170316	MDC004331.378:24044-24882	-2.69079	AT1G32560.1		Late embryogenesis abundant protein, group 1 protein
MDP0000147581	MDC004374.399:19-1173	1.79769e+308	AT5G61820.1		
MDP0000167786	MDC004427.577:2224-5046	-1.95698	AT1G73760.1		RING/U-box superfamily protein
0	MDC004475.200:15597-16341	5.32451	AT2G46150.1		Late embryogenesis abundant (LEA) hydroxyproline-rich glycoprotein family
MDP0000137468	MDC004475.200:20541-21775	3.67923	AT2G46150.1		Late embryogenesis abundant (LEA) hydroxyproline-rich glycoprotein family
MDP0000121566	MDC004513.349:138-370	1.79769e+308	AT2G14520.1		CBS domain-containing protein with a domain of unknown function (DUF21)
MDP0000065495	MDC004571.195:2433-4815	1.79769e+308	AT5G47080.1	CKB1	casein kinase II beta chain 1
MDP0000166057	MDC004613.228:11547-12800	1.79769e+308	AT5G05200.1		Protein kinase superfamily protein
MDP0000170777	MDC004652.210:48970-50473	1.79769e+308	AT5G53045.1		
MDP0000166875	MDC004673.395:10875-13759	-1.9178	AT2G38170.3	ATCAX1,CAX1,RCI4	cation exchanger 1
MDP0000249060	MDC004678.229:17526-20215	1.79769e+308	AT3G20640.1		basic helix-loop-helix (bHLH) DNA-binding superfamily protein
MDP0000421679	MDC004696.296:3968-4432	1.79769e+308	AT5G47910.1	ATRBOHD,RBOHD	respiratory burst oxidase homologue D
MDP0000237403	MDC004719.334:21812-23054	2.25432	AT5G42570.1		B-cell receptor-associated 31-like
MDP0000557465	MDC004732.225:9663-9840	1.79769e+308	AT2G19730.1		Ribosomal L28e protein family
MDP0000553127	MDC004757.363:10617-19812	2.12939	AT3G51680.1		NAD(P)-binding Rossmann-fold superfamily protein
MDP0000168735	MDC004766.533:4405-5962	1.79769e+308	AT5G13930.1	ATCHS,CHS,TT4	Chalcone and stilbene synthase family protein
MDP0000716308	MDC004766.552:11190-11877	1.79769e+308	AT5G13930.1	ATCHS,CHS,TT4	Chalcone and stilbene synthase family protein
MDP0000913661	MDC004804.396:7723-8379	1.88284	AT3G04300.1		RmlC-like cupins superfamily protein
0	MDC004821.392:9339-9512	1.79769e+308			
0	MDC004849.513:20-284	1.79769e+308			
MDP0000271982	MDC004885.793:4041-27077	5.6005	AT2G39340.1		SAC3/GANP/Nin1/mts3/eIF-3 p25 family
0	MDC004932.328:15182-15442	1.79769e+308			
MDP0000136847	MDC004932.329:23136-23429	1.79769e+308	AT1G73050.1		Glucose-methanol-choline (GMC) oxidoreductase family protein
0	MDC004932.329:29625-30381	3.26688			
MDP0000566117	MDC004936.114:731-11600	2.28845	AT4G20030.1		RNA-binding (RRM/RBD/RNP motifs) family protein
MDP0000170531	MDC004966.470:3884-8290	3.33886	AT1G70520.1	CRK2	cysteine-rich RLK (RECEPTOR-like protein kinase) 2
MDP0000148873	MDC004989.363:3672-5602	1.4007	AT4G27450.1		Aluminium induced protein with YGL and LRDR motifs
MDP0000121897	MDC005009.620:1896-3950	1.90789	AT1G27450.1	APT1,ATAPT1	adenine phosphoribosyl transferase 1
MDP0000147784	MDC005054.249:5101-5480	1.79769e+308	AT3G59110.1		Protein kinase superfamily protein
MDP0000137705	MDC005122.196:4548-6205	2.24	AT1G78440.1	ATGA2OX1,GA2OX1	Arabidopsis thaliana gibberellin 2-oxidase 1
MDP0000166259	MDC005161.483:10-1556	2.00714	AT2G38740.1		Haloacid dehalogenase-like hydrolase (HAD) superfamily protein
MDP0000275092	MDC005161.724:3466-11719	1.85637	AT2G38740.1		Haloacid dehalogenase-like hydrolase (HAD) superfamily protein
0	MDC005194.219:3090-3360	1.79769e+308			
MDP0000658768	MDC005200.174:34013-34414	3.23991	AT1G11090.1		alpha/beta-Hydrolases superfamily protein
MDP0000702868	MDC005243.382:9372-10625	2.27364	AT3G54420.1	ATCHITIV,ATEP3,CHIV,EP3	homolog of carrot EP3-3 chitinase
0	MDC005257.231:6449-6485	1.79769e+308			
0	MDC005259.535:4241-4601	1.79769e+308			
MDP0000273224	MDC005334.309:206-8584	-2.16795	AT5G53350.1	CLPX	CLP protease regulatory subunit X
MDP0000450601	MDC005362.240:6098-12887	1.79769e+308	AT3G11820.2	AT-SYR1,ATSYP121,ATSYR1,F	syntaxin of plants 121
MDP0000169025	MDC005381.283:29450-29871	1.79769e+308	AT1G12775.1		Pentatricopeptide repeat (PPR) superfamily protein
MDP0000678615	MDC005398.50:11698-13402	1.79769e+308	AT1G18400.1	BEE1	BR enhanced expression 1
MDP0000275383	MDC005401.171:13854-19232	1.79769e+308	AT5G01410.1	ATPDX1,ATPDX1.3,PDX1,PDX:	Aldolase-type TIM barrel family protein
MDP0000680695	MDC005410.203:4497-7949	1.79769e+308	AT5G18310.1		
MDP0000167207	MDC005423.311:6010-6700	3.44525	AT3G23240.1	ATERF1,ERF1	ethylene response factor 1
MDP0000249227	MDC005445.136:25859-31825	2.11406	AT3G53620.1	AtPPa4,PPa4	pyrophosphorylase 4
0	MDC005456.101:31462-31741	-2.20103			
0	MDC005479.52:19099-19266	1.79769e+308			
0	MDC005490.240:8153-8416	1.79769e+308	AT5G66180.3		S-adenosyl-L-methionine-dependent methyltransferases superfamily protein
MDP0000273966	MDC005490.243:32160-37061	2.95771	AT5G66160.1	ATRMR1,RMR1	receptor homology region transmembrane domain ring H2 motif protein 1
MDP0000273394	MDC005605.679:12982-15465	4.02694	AT1G06650.2		2-oxoglutarate (2OG) and Fe(II)-dependent oxygenase superfamily protein
0	MDC005625.410:4432-4879	1.79769e+308			
MDP0000257243	MDC005640.206:3033-6196	4.45015	AT1G73050.1		Glucose-methanol-choline (GMC) oxidoreductase family protein
0	MDC005648.396:5701-5725	1.79769e+308			
MDP0000679280	MDC005665.223:5730-9206	1.87481	AT3G16770.1	ATEBP,EBP,ERF72,RAP2.3	ethylene-responsive element binding protein
MDP0000807958	MDC005672.143:42154-43198	-3.70623	AT5G51550.1	EXL3	EXORDIUM like 3
MDP0000271007	MDC005673.323:4405-10172	1.73266	AT3G53970.1		proteasome inhibitor-related
MDP0000275079	MDC005673.340:1858-2675	2.64139	AT3G53970.1		proteasome inhibitor-related
MDP0000276802	MDC005701.391:5438-9015	-2.13886	AT1G64090.1	RTNLB3	Reticulan like protein B3
0	MDC005717.700:845-1059	1.79769e+308			
MDP0000271088	MDC005717.708:36286-47025	1.79769e+308	AT1G79830.1	GC5	golgin candidate 5
MDP0000784851	MDC005723.274:5102-5374	1.79769e+308	AT1G49000.1		
MDP0000931239	MDC005735.253:3467-3929	-1.71542	AT4G05320.2	UBQ10	polyubiquitin 10
MDP0000624279	MDC005778.440:252-801	1.79769e+308	AT4G20000.1		VQ motif-containing protein
MDP0000178868	MDC005799.514:759-806	-2.33718	AT5G17680.1		disease resistance protein (TIR-NBS-LRR class), putative
MDP0000232692	MDC005811.341:23198-25119	2.96568	AT5G53560.1	ATB5-A,ATCB5-E,B5 #2,CB5-E	cytochrome B5 isoform E
0	MDC005828.284:3570-4032	1.79769e+308			
MDP0000257423	MDC005856.445:16813-19559	1.45823	AT2G37430.1		C2H2 and C2HC zinc fingers superfamily protein
MDP0000776042	MDC005857.333:10992-11123	1.79769e+308	AT3G07700.3		Protein kinase superfamily protein
0	MDC005890.119:17882-18863	2.27453			
MDP0000279409	MDC005893.621:7340-26688	1.79769e+308	AT1G50030.1	TOR	target of rapamycin
0	MDC005927.393:5902-6584	3.89137	AT5G10830.1		S-adenosyl-L-methionine-dependent methyltransferases superfamily protein
MDP0000280686	MDC005983.384:14695-15478	1.79769e+308	AT4G13180.1		NAD(P)-binding Rossmann-fold superfamily protein
MDP0000507805	MDC005987.255:1295-1536	1.82319	AT2G38470.1	ATWRKY33,WRKY33	WRKY DNA-binding protein 33
MDP0000282531	MDC005992.425:10230-24855	1.79769e+308	AT2G16230.1		O-Glycosyl hydrolases family 17 protein
MDP0000713750	MDC006022.710:6794-7361	1.79769e+308	AT4G37710.1		VQ motif-containing protein
MDP0000281307	MDC006067.226:2770-5589	1.79769e+308	AT5G07910.1		Leucine-rich repeat (LRR) family protein
MDP0000280867	MDC006113.282:7307-21521	1.79769e+308	AT5G25757.1		RNA polymerase I-associated factor PAF67
MDP0000233439	MDC006132.110:38822-42176	1.79769e+308	AT2G31955.1	CNX2	cofactor of nitrate reductase and xanthine dehydrogenase 2
MDP0000233439	MDC006132.110:42437-42590	1.79769e+308	AT2G31955.1	CNX2	cofactor of nitrate reductase and xanthine dehydrogenase 2
MDP0000233440	MDC006132.110:44034-44983	2.81494	AT2G31945.1		
MDP0000344124	MDC006216.151:1502-1745	1.79769e+308			
MDP0000280820	MDC006246.458:682-958	2.04655			
0	MDC006260.462:8079-8672	2.73287	AT1G23440.1		Peptidase C15, pyroglutamyl peptidase I-like
MDP0000281098	MDC006273.129:20610-22757	-2.5382	AT5G51440.1		HSP20-like chaperones superfamily protein
MDP0000238184	MDC006273.74:424-2967	-2.52469	AT5G51440.1		HSP20-like chaperones superfamily protein
MDP0000032171	MDC006283.261:35240-41767	-1.94697	AT1G09310.1		Protein of unknown function, DUF538
0	MDC006289.408:7320-7584	-2.07297	AT2G45960.3	PIP1B, TMP-A, ATHH2, PIP1;2	plasma membrane intrinsic protein 1B
0	MDC006290.363:14600-14871	1.79769e+308			
MDP0000279018	MDC006290.371:8651-11538	3.88462	AT2G46150.1		Late embryogenesis abundant (LEA) hydroxyproline-rich glycoprotein family
MDP0000327743	MDC006290.371:8651-11538	4.09612			
MDP0000921896	MDC006365.287:19008-19524	-2.93166	AT5G09530.1		hydroxyproline-rich glycoprotein family protein
MDP0000278727	MDC006365.296:8642-9158	-3.15338	AT5G09530.1		hydroxyproline-rich glycoprotein family protein
0	MDC006367.239:347-584	1.79769e+308			
MDP0000278697	MDC006377.327:14719-15944	-2.64093			
MDP0000366350	MDC006462.291:4203-4755	-2.0333			
MDP0000279102	MDC006464.260:445-1034	2.00176	AT2G42160.1	BRIZ1	zinc finger (ubiquitin-hydrolase) domain-containing protein
MDP0000940872	MDC006465.421:24406-24762	4.01979	AT1G70160.1		
MDP0000694318	MDC006473.444:2709-3675	1.79769e+308	AT1G70250.1		receptor serine/threonine kinase, putative
MDP0000657053	MDC006504.484:24167-26272	-1.92738	AT4G14960.2	TUA6	Tubulin/FtsZ family protein
MDP0000281041	MDC006515.236:1405-3722	3.37856	AT3G02850.1	SKOR	STELAR K+ outward rectifier
MDP0000410264	MDC006528.136:5057-7941	-1.9936	AT2G37640.1	ATEXP3,ATEXPA3,ATHEXP AL	Barwin-like endoglucanases superfamily protein
MDP0000773502	MDC006536.318:82895-83101	1.79769e+308	AT5G64560.1	ATMGT9,MGT9,MRS2-2	magnesium transporter 9
MDP0000234325	MDC006553.299:18189-18514	3.21243	AT1G32230.2	ATP8,CEO,CEO1,RCD1	WWE protein-protein interaction domain protein family
MDP0000182716	MDC006600.356:5806-6097	1.93603	AT3G14470.1		NB-ARC domain-containing disease resistance protein
0	MDC006603.734:2300-2410	4.51059	AT5G39110.1		RmlC-like cupins superfamily protein
MDP0000182713	MDC006603.747:276-4246	1.80959	AT3G26430.1		GDSL-like Lipase/Acylhydrolase superfamily protein
MDP0000635152	MDC006630.203:6162-6951	1.79769e+308	AT2G36690.1		2-oxoglutarate (2OG) and Fe(II)-dependent oxygenase superfamily protein

MDP0000843913	MDC006630.345:13587-14886	1.79304	AT2G36690.1		2-oxoglutarate (2OG) and Fe(II)-dependent oxygenase superfamily protein
MDP0000552328	MDC006653.435:4109-5236	2.26965	AT1G75800.1		Pathogenesis-related thaumatin superfamily protein
MDP0000249299	MDC006684.239:7756-10611	-2.91344	AT3G21190.1		O-fucosyltransferase family protein
MDP0000249161	MDC006776.974:7590-21009	1.79769e+308	AT4G34580.1	COW1,SRH1	Sec14p-like phosphatidylinositol transfer family protein
MDP0000405576	MDC006783.229:11986-13497	1.79769e+308	AT1G52343.1		
MDP0000614645	MDC006784.251:4983-11622	2.1349	AT3G25655.1	IDL1	inflorescence deficient in abscission (IDA)-like 1
0	MDC006793.328:2502-2994	2.61703			
0	MDC006793.328:4674-5465	2.2282			
MDP0000248822	MDC006817.441:6965-9324	5.62372	AT2G26560.1	PLA IIA,PLA2A,PLP2	phospholipase A 2A
MDP0000814329	MDC006848.168:4784-9157	2.62974	AT3G06650.1	ACLB-1	ATP-citrate lyase B-1
MDP0000805422	MDC006858.526:1099-1672	1.86738	AT4G17500.1	ATERF-1,ERF-1	ethylene responsive element binding factor 1
MDP0000343634	MDC006858.527:5849-6150	-3.44095			
MDP0000238475	MDC006888.339:876-1073	1.79769e+308	AT3G30300.1		O-fucosyltransferase family protein
MDP0000178516	MDC006898.720:8191-8834	-2.21317	AT1G65295.1		
MDP0000136298	MDC006922.133:31989-32562	-3.54702	AT5G09530.1		hydroxyproline-rich glycoprotein family protein
MDP0000248516	MDC006922.133:35876-36560	-3.84131	AT5G09530.1		hydroxyproline-rich glycoprotein family protein
MDP0000137117	MDC006985.165:1223-3076	-1.92555	AT3G11400.1	ATEIF3G1,EIF3G1	eukaryotic translation initiation factor 3G1
0	MDC006994.179:16210-16462	1.79769e+308			
0	MDC006995.271:38249-38468	1.79769e+308			
MDP0000184167	MDC006998.275:5023-5392	1.79769e+308	AT1G80770.2	PDE318	P-loop containing nucleoside triphosphate hydrolases superfamily protein
MDP0000617009	MDC007021.246:25478-25865	-2.50768	AT2G46600.1		Calcium-binding EF-hand family protein
0	MDC007024.343:8632-8930	2.35329			
MDP0000182364	MDC007070.485:4341-4878	-2.28142	AT5G38760.1		Late embryogenesis abundant protein (LEA) family protein
MDP0000805474	MDC007073.330:12070-12457	1.79769e+308	AT3G09270.1	ATGSTU8,GSTU8	glutathione S-transferase TAU 8
0	MDC007088.513:4786-5156	1.79769e+308	AT5G24810.2		ABC1 family protein
MDP0000279516	MDC007100.397:805-12837	1.79769e+308	AT2G18470.1	PERK4	roline-rich extensin-like receptor kinase 4
MDP0000249404	MDC007109.204:49-2474	1.79769e+308	AT4G00590.1		N-terminal nucleophile aminohydrolases (Ntn hydrolases) superfamily protein
MDP0000147996	MDC007197.415:11084-11948	1.79769e+308	AT3G08690.1	ATUBC11,UBC11	ubiquitin-conjugating enzyme 11
MDP0000176426	MDC007216.431:6443-7603	1.79769e+308	AT1G68450.1		VQ motif-containing protein
0	MDC007223.300:45679-45974	-2.75104			
MDP0000729533	MDC007236.83:16404-17781	1.79769e+308	AT3G26040.1		HXXXD-type acyl-transferase family protein
MDP0000181621	MDC007245.594:763-2225	1.79769e+308	AT3G08880.1		
MDP0000693594	MDC007269.339:4912-5995	2.40891	AT4G37300.1	MEE59	maternal effect embryo arrest 59
MDP0000279107	MDC007287.149:7106-7274	1.79769e+308	AT2G27980.1		Acyl-CoA N-acyltransferase with RING/FYVE/PHD-type zinc finger domain
MDP0000811844	MDC007352.390:11700-12258	1.82193			
0	MDC007364.345:4479-4876	1.8905			
MDP0000147939	MDC007394.331:695-1727	-2.14127	AT3G55430.1		O-Glycosyl hydrolases family 17 protein
MDP0000180472	MDC007395.672:2994-3955	-2.52714	AT4G25040.1		Uncharacterised protein family (UPF0497)
MDP0000175691	MDC007406.309:1539-2750	1.79769e+308	AT1G52800.1		2-oxoglutarate (2OG) and Fe(II)-dependent oxygenase superfamily protein
MDP0000123467	MDC007407.185:1806-1906	1.79769e+308	AT5G13080.1	ATWRKY75,WRKY75	WRKY DNA-binding protein 75
MDP0000123467	MDC007407.185:1980-2255	1.79769e+308	AT5G13080.1	ATWRKY75,WRKY75	WRKY DNA-binding protein 75
MDP0000282711	MDC007441.706:4194-6829	1.9862	AT3G13710.1	PRA1.F4	prenylated RAB acceptor 1.F4
MDP0000231813	MDC007467.200:80611-80777	1.79769e+308	AT3G62580.1		Late embryogenesis abundant protein (LEA) family protein
0	MDC007474.161:727-1187	2.13649			
MDP0000281921	MDC007517.244:8152-8403	1.79769e+308	AT4G02920.1		
MDP0000231711	MDC007566.303:5209-5295	1.79769e+308	AT3G25590.1		
0	MDC007581.595:2473-2576	1.79769e+308	AT4G28460.1		unknown protein
MDP0000178934	MDC007586.376:13849-14356	-1.89038	AT2G25770.1		Polyketide cyclase/dehydrase and lipid transport superfamily protein
MDP0000238852	MDC007632.133:37-1671	1.79769e+308	AT5G15860.1	ATPCME,PCME	prenylcysteine methylesterase
MDP0000943804	MDC007651.256:10903-11228	1.79769e+308	AT4G32320.1	APX6	ascorbate peroxidase 6
MDP0000281059	MDC007667.245:11085-11336	1.79769e+308	AT1G71980.1		Protease-associated (PA) RING/U-box zinc finger family protein
MDP0000462809	MDC007696.347:224-1274	-2.36735	AT4G32390.1		Nucleotide-sugar transporter family protein
MDP0000284756	MDC007726.450:6011-6496	-1.90333	AT1G78880.1		Ubiquitin-specific protease family C19-related protein
0	MDC007779.587:2989-3343	1.79769e+308	AT3G14630.1	CYP72A14	cytochrome P450, family 72, subfamily A, polypeptide 14
0	MDC007779.587:3489-4308	5.81577	AT3G14680.1	CYP72A9	cytochrome P450, family 72, subfamily A, polypeptide 9
MDP0000320004	MDC007801.297:1668-14062	-2.60268	AT4G30600.1		signal recognition particle receptor alpha subunit family protein
MDP0000279567	MDC007839.76:255-4737	-1.98232	AT1G35140.1	EXL7,PHI-1	Phosphate-responsive 1 family protein
MDP0000279568	MDC007839.76:6045-12451	1.79769e+308	AT5G16800.2		Acyl-CoA N-acyltransferases (NAT) superfamily protein
MDP0000177906	MDC007908.251:15906-18464	3.66793	AT1G80840.1	ATWRKY40,WRKY40	WRKY DNA-binding protein 40
MDP0000156208	MDC007970.423:2201-3402	1.79769e+308	AT5G17820.1		Peroxidase superfamily protein
0	MDC008017.329:6473-7175	2.01024	AT2G46150.1		Late embryogenesis abundant (LEA) hydroxyproline-rich glycoprotein family
MDP0000176969	MDC008031.166:10358-10802	-1.71749	AT2G33820.1	ATMBAC1,MBAC1	Mitochondrial substrate carrier family protein
0	MDC008031.166:8984-9384	-1.91543	AT3G60030.1	SPL12	squamosa promoter-binding protein-like 12
0	MDC008070.309:6943-7245	1.79769e+308			
MDP0000178304	MDC008095.233:439-1308	1.79769e+308	AT3G09270.1	ATGSTU8,GSTU8	glutathione S-transferase TAU 8
MDP0000180902	MDC008107.194:8214-8566	1.79769e+308	AT1G52800.1		2-oxoglutarate (2OG) and Fe(II)-dependent oxygenase superfamily protein
MDP0000176147	MDC008111.220:3257-3489	1.79769e+308	AT3G08010.1	ATAB2	RNA binding
0	MDC008132.437:2842-3005	1.79769e+308			
MDP0000282113	MDC008133.431:23701-30184	-2.02622	AT5G39710.1	EMB2745	Tetratricopeptide repeat (TPR)-like superfamily protein
MDP0000124013	MDC008156.258:1804-1941	1.79769e+308	AT4G13250.1	NYC1	NAD(P)-binding Rossmann-fold superfamily protein
0	MDC008185.596:848-1396	4.18103			
MDP0000124065	MDC008191.272:1308-2582	1.33928	AT2G27830.1		
0	MDC008212.493:3616-4058	-2.22497	AT5G43180.1		Protein of unknown function, DUF599
0	MDC008216.318:26921-27156	1.79769e+308	AT4G20820.1		FAD-binding Berberine family protein
0	MDC008227.470:6-265	2.67388			
MDP0000248806	MDC008228.299:15226-24695	1.79769e+308	AT3G26070.1		Plastid-lipid associated protein PAP / fibrillin family protein
MDP0000280265	MDC008231.317:2716-4497	2.51441	AT5G24090.1	ATCHIA,CHIA	chitinase A
MDP0000182082	MDC008275.176:13207-16686	1.834	AT1G10670.1	ACLA-1	ATP-citrate lyase A-1
MDP0000149907	MDC008363.128:1345-1577	3.47723	AT5G51970.1		GroES-like zinc-binding alcohol dehydrogenase family protein
MDP0000149907	MDC008363.128:1584-2882	2.61724	AT5G51970.1		GroES-like zinc-binding alcohol dehydrogenase family protein
MDP0000281408	MDC008378.283:5092-5500	1.92703	AT1G36370.1	SHM7	serine hydroxymethyltransferase 7
0	MDC008382.148:13043-13406	1.79769e+308			
MDP0000281701	MDC008389.364:4307-10927	-2.13921	AT2G34770.1	ATFAH1,FAH1	fatty acid hydroxylase 1
MDP0000279279	MDC008406.198:19837-20128	1.79769e+308	AT4G21390.1	B120	S-locus lectin protein kinase family protein
MDP0000836165	MDC008510.241:8559-10423	3.04968	AT4G02330.1	ATPMEPCRB	Plant invertase/pectin methylesterase inhibitor superfamily
0	MDC008521.354:1264-1327	1.79769e+308			
MDP0000720186	MDC008527.309:2198-2323	1.79769e+308	AT2G34250.1		SecY protein transport family protein
MDP0000857802	MDC008558.180:29014-29454	1.79769e+308	AT1G30950.1	UFO	F-box family protein
0	MDC008563.271:10114-10663	2.28791	AT1G75620.1		glyoxal oxidase-related protein
MDP0000149770	MDC008563.295:2531-3710	1.79769e+308	AT5G40240.2		nodulin MtN21 /EamA-like transporter family protein
MDP0000873376	MDC008586.420:17793-18695	-1.96409	AT1G72230.1		Cupredoxin superfamily protein
0	MDC008600.171:902-1400	4.88147			
MDP0000285032	MDC008614.269:11939-16468	2.46831	AT3G18830.1	ATPLT5,ATPMT5,PMT5	polyol/monosaccharide transporter 5
MDP0000670527	MDC008741.603:1444-2027	-2.4068	AT2G27420.1		Cysteine proteinases superfamily protein
MDP0000124372	MDC008769.540:981-2223	1.79769e+308	AT2G35930.1	PUB23	plant U-box 23
MDP0000190474	MDC008769.546:2266-3508	1.79769e+308	AT2G35930.1	PUB23	plant U-box 23
MDP0000191423	MDC008781.274:12188-14442	2.96124	AT1G22170.1		Phosphoglycerate mutase family protein
MDP0000191423	MDC008781.274:14553-14944	2.64127	AT1G22170.1		Phosphoglycerate mutase family protein
MDP0000288293	MDC008798.391:19315-20212	5.12026	AT1G24020.1	MLP423	MLP-like protein 423
MDP0000185506	MDC008808.256:4241-10398	-2.75566	AT5G12190.1		RNA-binding (RRM/RBD/RNP motifs) family protein
MDP0000188275	MDC008847.375:4627-4939	1.79769e+308	AT1G56660.1		
MDP0000287351	MDC008886.243:375-5075	1.79769e+308	AT5G36930.2		Disease resistance protein (TIR-NBS-LRR class) family
MDP0000694597	MDC008926.173:3439-3781	-2.3258	AT5G59320.1	LTP3	lipid transfer protein 3
MDP0000773415	MDC008930.262:14990-15201	1.79769e+308	AT1G05180.1	AXR1	NAD(P)-binding Rossmann-fold superfamily protein
MDP0000290937	MDC008947.382:14535-20023	3.12302			
MDP0000347336	MDC008953.300:591-12745	2.93419			
0	MDC008958.527:6035-6390	1.79769e+308			
MDP0000188138	MDC008981.224:3552-6255	1.79769e+308	AT5G21940.1		
MDP0000090732	MDC009045.262:43011-46844	2.52716	AT5G48100.1	ATLAC15,LAC15,TT10	Laccase/Diphenol oxidase family protein
MDP0000291620	MDC009067.527:8837-9392	-2.07458	AT1G58170.1		Disease resistance-responsive (dirigent-like protein) family protein
MDP0000287628	MDC009095.320:4320-11111	-1.76609	AT3G58810.1	ATMTP3,ATMTPA2,MTP3,MTP	metal tolerance protein A2
MDP0000193050	MDC009100.720:14269-19338	2.72097	AT5G26340.1	ATSTP13,MSS1,STP13	Major facilitator superfamily protein
0	MDC009100.743:10602-10864	1.79769e+308			
0	MDC009139.374:7848-8934	2.91798	AT2G46150.1		Late embryogenesis abundant (LEA) hydroxyproline-rich glycoprotein family
MDP0000746652	MDC009231.274:4163-4333	1.79769e+308	AT1G58440.1	SQE1,XF1	FAD/NAD(P)-binding oxidoreductase family protein
MDP0000138090	MDC009247.159:1041-7164	2.43611	AT2G47800.1	ATMRP4,EST3,MRP4	multidrug resistance-associated protein 4
MDP0000841703	MDC009250.269:24582-24915	1.79769e+308	AT3G11440.1	ATMYB65,MYB65	myb domain protein 65
MDP0000361876	MDC009251.558:7920-10453	-3.37738	AT4G03210.1	XTH9	xyloglucan endotransglucosylase/hydrolase 9
0	MDC009291.198:2462-2529	1.79769e+308			
MDP0000124719	MDC009297.162:8847-9498	-1.9762	AT5G47610.1		RING/U-box superfamily protein
MDP0000239720	MDC009336.266:5288-15663	2.26922	AT5G05930.1	ATGC1,GC1	guanylyl cyclase 1
MDP0000671324	MDC009349.320:566-1709	1.79769e+308	AT4G10490.1		2-oxoglutarate (2OG) and Fe(II)-dependent oxygenase superfamily protein
MDP0000504527	MDC009360.278:896-3116	3.05153	AT1G78860.1		D-mannose binding lectin protein with Apple-like carbohydrate-binding domain
MDP0000262674	MDC009368.194:14350-15917	-1.88738	AT1G07473.1		
0	MDC009393.166:1252-1510	1.79769e+308			
MDP0000685425	MDC009404.603:2897-8558	1.79769e+308	AT5G17680.1		disease resistance protein (TIR-NBS-LRR class), putative
MDP0000941000	MDC009483.39:12100-14889	2.87019	AT1G47670.1		Transmembrane amino acid transporter family protein
MDP0000290562	MDC009567.313:17183-20584	-1.89197	AT3G55770.1		GATA type zinc finger transcription factor family protein
MDP0000936748	MDC009600.163:20757-21849	-1.7773	AT2G33850.1		
MDP0000150032	MDC009604.192:12640-14455	1.79769e+308	AT4G13220.1		
MDP0000285243	MDC009616.38:20066-24921	2.38728	AT4G19810.1		Glycosyl hydrolase family protein with chitinase insertion domain
MDP0000285243	MDC009616.38:20066-24921	1.79769e+308	AT4G19810.1		Glycosyl hydrolase family protein with chitinase insertion domain
0	MDC009630.367:24050-24262	1.79769e+308	AT2G03810.4		18S pre-ribosomal assembly protein gar2-related
MDP0000187983	MDC009673.411:12650-17839	1.79769e+308	AT2G41040.1		S-adenosyl-L-methionine-dependent methyltransferases superfamily protein
MDP0000290045	MDC009727.218:5205-32777	1.79769e+308	AT2G42810.2	PAPP5,PP5,PP5.2	protein phosphatase 5.2
MDP0000125002	MDC009738.162:4550-8590	1.79769e+308	AT1G26810.1	GALT1	galactosyltransferase1
MDP0000757636	MDC009757.32:1532-2923	-1.71887	AT3G08940.2	LHCB4.2	light harvesting complex photosystem II
MDP0000193574	MDC009761.287:26902-27496	1.79769e+308			
MDP0000353412	MDC009761.287:29079-29304	1.79769e+308			
MDP0000231304	MDC009798.251:9799-13128	1.78051	AT2G33150.1	KAT2,PED1,PKT3	peroxisomal 3-ketoacyl-CoA thiolase 3
MDP0000239947	MDC009815.256:8947-11799	2.62433	AT3G11180.1		2-oxoglutarate (2OG) and Fe(II)-dependent oxygenase superfamily protein
MDP0000292200	MDC009823.281:1297-20823	1.79769e+308	AT3G01610.1	CDC48C,emb1354	cell division cycle 48C
MDP0000292200	MDC009823.281:1297-20823	1.79769e+308	AT3G01610.1	CDC48C,emb1354	cell division cycle 48C
MDP0000287919	MDC009830.388:12732-14292	1.79769e+308	AT5G13930.1	ATCHS,CHS,TT4	Chalcone and stilbene synthase family protein

0	MDC009830.388:3008-4348	1.79769e+308	AT5G13930.1	CHS, TT4, ATCHS	Chalcone and stilbene synthase family protein
MDP0000703851	MDC009836.225:17066-18219	2.31731	AT4G37300.1	MEE59	maternal effect embryo arrest 59
MDP0000850409	MDC009848.204:8363-8600	-4.67506			
MDP0000125095	MDC009861.437:3891-4319	-2.80388	AT5G38760.1		Late embryogenesis abundant protein (LEA) family protein
MDP0000291925	MDC009861.464:1768-13770	-2.02662	AT5G38760.1		Late embryogenesis abundant protein (LEA) family protein
MDP0000187103	MDC009909.204:5928-6981	1.79769e+308	AT5G67080.1	MAPKKK19	mitogen-activated protein kinase kinase kinase 19
MDP0000454694	MDC009933.158:11532-19587	-2.20363	AT1G72820.1		Mitochondrial substrate carrier family protein
MDP0000125143	MDC009940.459:13383-13777	1.79769e+308	AT2G19385.1		zinc ion binding
MDP0000287566	MDC009986.307:3331-10534	2.41066	AT5G49230.1	HRB1	Drought-responsive family protein
MDP0000191667	MDC010015.212:741-6386	2.23913	AT4G13430.1	ATLEUC1,IIL1	isopropyl malate isomerase large subunit 1
MDP0000289011	MDC010104.925:5261-8066	2.73062	AT3G09270.1	ATGSTU8,GSTU8	glutathione S-transferase TAU 8
MDP0000285894	MDC010113.132:1890-6718	1.79769e+308	AT5G53130.1	ATCNGC1,CNGC1	cyclic nucleotide gated channel 1
MDP0000645502	MDC010137.193:5899-6763	1.79769e+308			
0	MDC010174.397:1163-1411	1.79769e+308	AT2G46150.1		Late embryogenesis abundant (LEA) hydroxyproline-rich glycoprotein family
MDP0000909081	MDC010177.373:8987-12973	3.00973	AT2G37790.1		NAD(P)-linked oxidoreductase superfamily protein
MDP0000776953	MDC010183.335:5076-5272	1.79769e+308	AT5G63310.1	ATNDPK2,NDPK IA,NDPK IA IA	nucleoside diphosphate kinase 2
MDP0000125336	MDC010195.886:3529-4847	-3.53045	AT1G55260.1		Bifunctional inhibitor/lipid-transfer protein/seed storage 2S albumin superfamily protein
MDP0000777260	MDC010201.200:11935-13257	1.6832	AT5G59530.1		2-oxoglutarate (2OG) and Fe(II)-dependent oxygenase superfamily protein
MDP0000138612	MDC010224.339:7684-14666	1.79769e+308			
MDP0000116244	MDC010239.242:2210-2815	3.42985	AT1G24020.1	MLP423	MLP-like protein 423
MDP0000116244	MDC010239.242:2917-3044	1.79769e+308	AT1G24020.1	MLP423	MLP-like protein 423
MDP0000638442	MDC010241.223:2599-3910	2.42596	AT5G51970.1		GroES-like zinc-binding alcohol dehydrogenase family protein
MDP0000188052	MDC010241.225:11561-12807	2.52239	AT5G51970.1		GroES-like zinc-binding alcohol dehydrogenase family protein
MDP0000251295	MDC010246.430:21589-22966	2.2344	AT1G05010.1	ACO4,EAT1,EFE	ethylene-forming enzyme
MDP0000125388	MDC010264.104:2067-2797	1.79769e+308	AT5G35960.1		Protein kinase family protein
MDP0000668138	MDC010296.225:19484-19807	1.79769e+308	AT3G52250.1		Duplicated homeodomain-like superfamily protein
MDP0000258062	MDC010332.272:588-1152	1.79769e+308	AT5G10350.1		RNA-binding (RRM/RBD/RNP motifs) family protein
MDP0000233489	MDC010426.795:7913-8261	1.79769e+308	AT5G48820.1	ICK6,KRP3	inhibitor/interactor with cyclin-dependent kinase
MDP0000292448	MDC010444.140:7446-9204	-3.31146	AT5G38020.1		S-adenosyl-L-methionine-dependent methyltransferases superfamily protein
MDP0000796596	MDC010462.160:15024-15360	1.79769e+308			
MDP0000588940	MDC010473.396:14867-15858	-3.86943	AT3G27200.1		Cupredoxin superfamily protein
0	MDC010474.340:3456-4185	1.79769e+308	AT2G46150.1		Late embryogenesis abundant (LEA) hydroxyproline-rich glycoprotein family
0	MDC010476.238:18498-19659	4.06594	AT2G46150.1		Late embryogenesis abundant (LEA) hydroxyproline-rich glycoprotein family
MDP0000292131	MDC010476.238:34773-35325	3.99199	AT2G46150.1		Late embryogenesis abundant (LEA) hydroxyproline-rich glycoprotein family
0	MDC010507.323:2408-2497	1.79769e+308			
MDP0000288622	MDC010541.240:1517-3505	1.79769e+308	AT5G22860.1		Serine carboxypeptidase S28 family protein
MDP0000194046	MDC010550.498:7035-7904	1.79769e+308	AT3G09270.1	ATGSTU8,GSTU8	glutathione S-transferase TAU 8
MDP0000566057	MDC010577.405:11254-12570	1.79769e+308	AT2G36690.1		2-oxoglutarate (2OG) and Fe(II)-dependent oxygenase superfamily protein
MDP0000557646	MDC010584.403:9855-16868	1.79769e+308	AT3G05500.1		Rubber elongation factor protein (REF)
0	MDC010589.234:1224-1539	1.79769e+308			
MDP0000440922	MDC010598.348:5127-6664	2.58806	AT5G36110.1	CYP716A1	cytochrome P450, family 716, subfamily A, polypeptide 1
MDP0000292176	MDC010630.204:5140-5351	1.79769e+308	AT1G08450.1	AtCRT3,CRT3,EBS2,PSL1	calreticulin 3
MDP0000191951	MDC010729.433:8183-8534	1.79769e+308			
MDP0000259250	MDC010764.217:2450-4361	-2.08975	AT4G11220.1	BTI2,RTNLB2	VIRB2-interacting protein 2
MDP0000549056	MDC010870.164:3469-9528	3.09695	AT1G68840.1	EDF2,RAP2.8,RAV2,TEM2	related to ABI3/VP1 2
0	MDC010876.313:27953-28158	1.79769e+308			
0	MDC010912.583:5714-6300	3.70536	AT2G36380.1	PDR6, ATPDR6	pleiotropic drug resistance 6
MDP0000893506	MDC010932.713:30968-31739	1.79769e+308	AT1G68450.1		VQ motif-containing protein
MDP0000187492	MDC010968.194:35563-43410	2.47873	AT2G26780.1		ARM repeat superfamily protein
0	MDC010981.245:18382-19274	3.15585	AT3G61510.1	ACS1, AT-ACS1	ACC synthase 1
MDP0000289768	MDC011000.158:4623-5925	1.79769e+308	AT3G23250.1	ATMYB15,ATY19,MYB15	myb domain protein 15
MDP0000126098	MDC011163.578:6912-8415	1.79769e+308	AT5G20030.1		Plant Tudor-like RNA-binding protein
MDP0000188976	MDC011170.439:2837-3077	1.79769e+308	AT2G15960.1		
MDP0000126152	MDC011264.166:12266-14181	-2.83625	AT1G09575.1		Protein of unknown function (DUF607)
MDP0000880247	MDC011311.340:11831-15202	2.41446	AT1G30755.1		Protein of unknown function (DUF668)
MDP0000906703	MDC011337.355:7470-8262	-2.25482	AT3G59400.1	GUN4	enzyme binding;tetrapyrrole binding
0	MDC011359.150:1008-1311	1.79769e+308			
0	MDC011372.235:19894-20135	1.79769e+308			
MDP0000560179	MDC011400.321:16471-19830	1.79769e+308	AT1G69370.1	cm-3,CM3	chorismate mutase 3
0	MDC011401.250:14560-14880	-2.31808			
MDP0000138204	MDC011412.200:2474-2656	1.79769e+308	AT1G01650.1	ATSPPL4,SPPL4	SIGNAL PEPTIDE PEPTIDASE-LIKE 4
MDP0000415910	MDC011412.206:13093-13390	1.79769e+308	AT4G01840.1	ATKCO5,ATTPK5,KCO5,TPK5	Ca2+ activated outward rectifying K+ channel 5
MDP0000290114	MDC011437.437:35717-37203	-2.74555	AT2G01770.1	ATVIT1,VIT1	vacuolar iron transporter 1
MDP0000031172	MDC011441.318:5874-6624	1.79769e+308	AT4G38620.1	ATMYB4,MYB4	myb domain protein 4
0	MDC011449.97:11886-12570	1.79769e+308	AT2G18680.1		unknown protein
MDP0000286168	MDC011456.83:10260-11593	-2.17958	AT5G13490.1	AAC2	ADP/ATP carrier 2
0	MDC011459.391:12253-12872	1.79769e+308	AT2G39210.1		Major facilitator superfamily protein
MDP0000361947	MDC011469.280:8071-8371	3.5144			
MDP0000661951	MDC011469.280:8412-12283	2.3818	AT4G11820.2	EMB2778,FKP1,HMGS,MVA1	hydroxymethylglutaryl-CoA synthase / HMG-CoA synthase / 3-hydroxy-3-methylglutaryl coenzyme A synthase
MDP0000902434	MDC011537.547:10554-10740	1.79769e+308	AT4G05030.1		Copper transport protein family
MDP0000902434	MDC011537.547:9633-10450	2.21998	AT4G05030.1		Copper transport protein family
0	MDC011542.145:14812-15089	-2.78853			
MDP0000290254	MDC011554.266:8335-11088	2.78301			
MDP0000185643	MDC011581.443:34972-37559	-2.0884	AT1G70090.1	GATL9,LGT8	glucosyl transferase family 8
MDP0000185643	MDC011581.443:34972-37559	1.79769e+308	AT1G70090.1	GATL9,LGT8	glucosyl transferase family 8
0	MDC011583.325:5690-5843	1.79769e+308			
MDP0000150382	MDC011596.290:3499-8925	2.75396	AT2G43820.1	ATSAGT1,GT,SAGT1,SGT1,UC	UDP-glucosyltransferase 74F2
0	MDC011598.529:10860-11398	2.42453			
MDP0000926540	MDC011602.378:2520-3183	1.88338	AT3G02430.1		Protein of unknown function (DUF679)
0	MDC011603.242:16362-17137	1.86683	AT4G33720.1		CAP (Cysteine-rich secretory proteins, Antigen 5, and Pathogenesis-related 1 protein) superfamily protein
MDP0000199152	MDC011612.43:0-3077	2.18079	AT4G16730.1	TPS02	terpene synthase 02
MDP0000139971	MDC011642.187:6067-13143	2.14987	AT4G36710.1		GRAS family transcription factor
MDP0000126455	MDC011659.224:3319-4725	-3.00214	AT1G10200.1	WLIM1	GATA type zinc finger transcription factor family protein
MDP0000292543	MDC011678.338:3910-4160	1.79769e+308	AT3G22380.2	TIC	time for coffee
MDP0000251253	MDC011704.133:11215-11348	1.79769e+308	AT1G76490.1	HMG1,HMGR1	hydroxy methylglutaryl CoA reductase 1
MDP0000771887	MDC011723.416:11571-12015	-2.22297	AT1G60950.1	ATFD2,FED A	2Fe-2S ferredoxin-like superfamily protein
MDP0000328550	MDC011745.99:15351-15722	-2.18845			
0	MDC011770.416:7739-8412	2.51628	AT2G46150.1		Late embryogenesis abundant (LEA) hydroxyproline-rich glycoprotein family
0	MDC011773.84:2484-3065	5.81577	AT2G23270.1		unknown protein
MDP0000873235	MDC011784.207:16-913	2.39345	AT3G54420.1	ATCHITIV,ATEP3,CHIV,EP3	homolog of carrot EP3-3 chitinase
MDP0000430546	MDC011784.298:1990-4727	2.46989	AT3G54420.1	ATCHITIV,ATEP3,CHIV,EP3	homolog of carrot EP3-3 chitinase
MDP0000336546	MDC011792.236:31818-38424	1.79769e+308	AT3G27460.1		SGF29 tudor-like domain
MDP0000672129	MDC011802.265:2819-3744	-1.97863	AT3G26420.1	ATRZ-1A	RNA-binding (RRM/RBD/RNP motifs) family protein with retrovirus zinc finger-like domain
MDP0000618065	MDC011822.207:10507-11080	1.79769e+308	AT3G11820.2	AT-SYR1,ATSYP121,ATSYR1,F	syntaxin of plants 121
MDP0000416120	MDC011856.191:7049-7379	1.79769e+308	AT4G27060.1	CN,SPR2,TOR1	ARM repeat superfamily protein
0	MDC011857.111:12385-12818	-2.80994			
0	MDC011938.114:11698-11941	1.79769e+308			
MDP0000259719	MDC011953.180:21178-29290	2.13451	AT1G21480.1		Exostosin family protein
0	MDC011960.157:14970-15370	1.79769e+308			
MDP0000203230	MDC011969.177:1032-2423	5.0603	AT5G38940.1		RmlC-like cupins superfamily protein
MDP0000139824	MDC011983.274:13866-23337	-2.1557	AT1G34630.1		
MDP0000342813	MDC011995.314:7397-7948	1.79769e+308	AT2G23090.1		Uncharacterised protein family SERF
MDP0000576584	MDC011995.316:6927-7232	1.79769e+308	AT5G64160.1		
MDP0000910523	MDC011998.433:31142-33376	2.47451	AT5G05320.1		FAD/NAD(P)-binding oxidoreductase family protein
MDP0000647373	MDC012019.302:8116-8353	1.79769e+308			
MDP0000140046	MDC012024.335:4054-9352	2.79415	AT5G47770.1	FPS1	farnesyl diphosphate synthase 1
0	MDC012039.280:32641-32933	1.79769e+308			
MDP0000750556	MDC012049.250:246-726	3.88038	AT1G24020.1	MLP423	MLP-like protein 423
MDP0000259661	MDC012049.376:1086-2288	3.32901			
MDP0000418277	MDC012049.376:7654-8475	4.23797			
MDP0000260110	MDC012049.377:1467-1947	3.64793	AT1G24020.1	MLP423	MLP-like protein 423
MDP0000611200	MDC012049.378:11944-12424	4.45514	AT1G24020.1	MLP423	MLP-like protein 423
MDP0000195614	MDC012049.378:15381-15861	3.37256	AT1G24020.1	MLP423	MLP-like protein 423
MDP0000293143	MDC012049.378:7676-9891	3.59297	AT1G24020.1	MLP423	MLP-like protein 423
MDP0000241271	MDC012083.378:41-1721	-2.27036	AT3G06778.1		Chaperone DnaJ-domain superfamily protein
MDP0000152278	MDC012083.521:11-527	4.0936	AT4G16740.1	ATTPS03,TPS03	terpene synthase 03
MDP0000126789	MDC012097.411:814-2116	1.79769e+308	AT1G07230.1	NPC1	non-specific phospholipase C1
MDP0000233177	MDC012168.191:14092-15547	-3.4026	AT1G10550.1	XET,XTH33	xyloglucan:xyloglucosyl transferase 33
MDP0000241358	MDC012226.83:8174-8929	3.45922	AT1G74950.1	JAZ2,TIFY10B	TIFY domain/Divergent CCT motif family protein
MDP0000126892	MDC012245.148:3093-3709	1.79769e+308	AT4G38060.2		
MDP0000295823	MDC012255.109:11234-11389	1.79769e+308	AT5G20040.1	ATIPT9,IPT9	isopentenyltransferase 9
0	MDC012256.180:17521-17661	1.79769e+308			
0	MDC012284.228:14592-15163	3.41084			
MDP0000126954	MDC012310.593:2815-3479	1.79769e+308			
0	MDC012310.599:10117-10381	1.79769e+308			
MDP0000241432	MDC012341.121:5328-14277	2.20523	AT1G60900.1		U2 snRNP auxilliary factor, large subunit, splicing factor
MDP0000126976	MDC012346.135:2972-4484	-2.48248	AT2G33510.1		
0	MDC012355.316:11597-11962	1.79769e+308			
MDP0000139052	MDC012379.228:1791-2421	2.02621	AT4G20780.1	CML42	calmodulin like 42
0	MDC012392.481:2083-2367	1.79769e+308	AT2G29420.1	ATGSTU7, GST25, GSTU7	glutathione S-transferase tau 7
0	MDC012392.481:2550-3061	4.57808	AT3G09270.1	ATGSTU8, GSTU8	
MDP0000151934	MDC012402.49:8557-11135	-2.01946	AT4G28850.1	ATXTH26,XTH26	xyloglucan endotransglucosylase/hydrolase 26
MDP0000294379	MDC012403.580:3692-9461	3.59693	AT1G24020.1	MLP423	MLP-like protein 423
MDP0000144836	MDC012403.589:9512-9725	3.55338			
MDP0000201530	MDC012413.391:9456-9744	1.79769e+308	AT5G14570.1	ATNRT2.7,NRT2.7	high affinity nitrate transporter 2.7
MDP0000152368	MDC012454.133:870-5284	1.79769e+308	AT5G60370.1		
0	MDC012466.566:156-300	1.79769e+308			
0	MDC012491.391:15695-16226	-1.79382			
0	MDC012493.147:8427-8656	1.79769e+308			
MDP0000609876	MDC012499.268:30672-31306	5.56244	AT5G28640.1	AN3,ATGIF1,GIF,GIF1	SSXT family protein
MDP0000903805	MDC012506.282:5827-8190	3.70347	AT1G73050.1		Glucose-methanol-choline (GMC) oxidoreductase family protein
MDP0000202716	MDC012509.602:2756-6464	1.84839	AT2G41690.1	AT-HSFB3,HSFB3	heat shock transcription factor B3
MDP0000197764	MDC012535.429:23420-31367	1.79769e+308	AT5G61450.1		P-loop containing nucleoside triphosphate hydrolases superfamily protein
MDP0000427172	MDC012537.136:2027-2365	1.79769e+308	AT1G69060.1		Chaperone DnaJ-domain superfamily protein

MDP0000427172	MDC012537.136:2472-3087	5.39463	AT1G69060.1		Chaperone DnaJ-domain superfamily protein
MDP0000648218	MDC012539.194:16182-17073	1.9807	AT1G23710.1		Protein of unknown function (DUF1645)
MDP0000150727	MDC012578.402:50168-50408	1.79769e+308	AT1G50500.1	ATVPS53,HIT1,VPS53	Membrane trafficking VPS53 family protein
MDP0000127122	MDC012582.369:20321-21404	-1.95481	AT2G32300.1	UCC1	uclacyanin 1
MDP0000708928	MDC012593.380:41318-42423	-3.50148	AT2G05070.1	LHCB2,LHCB2.2	photosystem II light harvesting complex gene 2.2
MDP0000152323	MDC012595.126:7479-7658	1.79769e+308	AT3G22430.1		
MDP0000127134	MDC012597.1552:1503-2265	3.29898	AT3G23240.1	ATERF1,ERF1	ethylene response factor 1
MDP0000205052	MDC012623.194:30088-30799	1.79769e+308			
MDP0000300351	MDC012623.194:31381-34645	1.79769e+308			
MDP0000197224	MDC012641.253:15589-15898	1.9322			
MDP0000127185	MDC012660.72:295-5944	1.79769e+308	AT5G14270.2	ATBET9,BET9	bromodomain and extraterminal domain protein 9
MDP0000334047	MDC012688.235:31743-33240	3.21746	AT1G01720.1	ANAC002,ATAF1	NAC (No Apical Meristem) domain transcriptional regulator superfamily protein
0	MDC012696.212:377-1037	1.91257			
MDP0000809488	MDC012716.217:13456-14896	1.86079	AT4G27450.1		Aluminium induced protein with YGL and LRDR motifs
0	MDC012733.121:11561-12529	1.79769e+308	AT4G11280.1	ACS6, ATACS6	1-aminocyclopropane-1-carboxylic acid (acc) synthase 6
MDP0000851204	MDC012735.249:4715-5222	-1.89038	AT2G25770.1		Polyketide cyclase/dehydrase and lipid transport superfamily protein
MDP0000241694	MDC012816.263:1287-2659	3.41245			
MDP0000299311	MDC012816.264:988-1468	4.67582	AT1G24020.1	MLP423	MLP-like protein 423
MDP0000295542	MDC012816.382:14124-14604	5.13553	AT1G24020.1	MLP423	MLP-like protein 423
MDP0000295543	MDC012816.382:17333-17813	5.13278	AT1G24020.1	MLP423	MLP-like protein 423
MDP0000814399	MDC012816.382:20717-21197	4.71788	AT1G24020.1	MLP423	MLP-like protein 423
MDP0000295540	MDC012816.382:7552-8032	4.73806	AT1G24020.1	MLP423	MLP-like protein 423
MDP0000294030	MDC012817.651:4113-4501	1.79769e+308			
MDP0000127346	MDC012859.232:2261-2690	1.79769e+308	AT5G16940.1		carbon-sulfur lyases
MDP0000364657	MDC012885.350:14190-14436	-1.84875			
MDP0000470916	MDC012888.280:16453-18375	2.25112	AT5G20230.1	ATBCB,BCB,SAG14	blue-copper-binding protein
MDP0000808076	MDC012888.280:5525-6239	2.08673	AT5G20230.1	ATBCB,BCB,SAG14	blue-copper-binding protein
0	MDC012892.148:4018-4407	1.79769e+308			
MDP0000155307	MDC012898.383:1071-4451	-1.78424	AT5G66740.1		Protein of unknown function (DUF620)
0	MDC012900.215:12407-12925	1.79769e+308	AT5G01710.1		methyltransferases
MDP0000879217	MDC012905.365:22031-22294	1.79769e+308	AT5G58860.1	CYP86,CYP86A1	cytochrome P450, family 86, subfamily A, polypeptide 1
MDP0000260620	MDC012905.365:5717-11456	-2.31112	AT2G29640.1	JOSL	JOSEPHIN-like protein
MDP0000241771	MDC012934.235:1022-7184	1.97977	AT2G22250.2	AAT,ATAAT,MEE17	aspartate aminotransferase
MDP0000697676	MDC012951.271:9076-10542	1.79769e+308	AT5G02230.1		Haloacid dehalogenase-like hydrolase (HAD) superfamily protein
MDP0000543204	MDC012957.285:3141-3411	1.79769e+308			
MDP0000141463	MDC012987.400:9772-11178	-3.00214	AT1G10200.1	WLIM1	GATA type zinc finger transcription factor family protein
MDP0000322416	MDC013003.189:92143-92977	1.79769e+308	AT5G16360.1		NC domain-containing protein-related
MDP0000297138	MDC013044.213:3080-8904	1.79769e+308	AT4G33580.1	ATBCA5,BCA5	beta carbonic anhydrase 5
MDP0000744506	MDC013064.706:8737-8927	1.79769e+308	AT1G58050.1		RNA helicase family protein
MDP0000201700	MDC013070.55:5428-5946	-4.65078	AT5G59845.1		Gibberellin-regulated family protein
MDP0000295655	MDC013084.249:842-11955	1.79769e+308	AT5G39890.1		Protein of unknown function (DUF1637)
MDP0000198406	MDC013085.232:856-1627	1.74151	AT4G03510.1	ATRMA1,RMA1	RING membrane-anchor 1
MDP0000943292	MDC013096.127:30413-30872	3.16275	AT2G47485.1		
MDP0000298575	MDC013096.127:5650-13638	1.79769e+308	AT1G68190.1		B-box zinc finger family protein
MDP0000298767	MDC013101.424:1874-8337	1.79769e+308	AT5G13020.1		Emsy N Terminus (ENT)/ plant Tudor-like domains-containing protein
0	MDC013104.583:2153-2490	1.79769e+308			
MDP0000391210	MDC013104.650:7020-7701	1.79769e+308	AT5G06320.1	NHL3	NDR1/HIN1-like 3
MDP0000298031	MDC013115.223:1700-1934	1.79769e+308	AT4G19210.1	ATRLI2,RLI2	RNAse l inhibitor protein 2
MDP0000297093	MDC013124.81:6887-8992	-2.2894	AT3G02380.1	ATCOL2,COL2	CONSTANS-like 2
MDP0000775613	MDC013124.82:36109-36537	-2.59035	AT5G38760.1		Late embryogenesis abundant protein (LEA) family protein
MDP0000729348	MDC013141.176:13225-14599	1.79769e+308	AT3G48180.1		
MDP0000294360	MDC013237.128:1097-2970	1.96755	AT1G72310.1	ATL3	RING/U-box superfamily protein
MDP0000746564	MDC013254.184:20555-22846	1.79769e+308	AT5G28770.2	AtbZIP63,BZO2H3	bZIP transcription factor family protein
MDP0000231605	MDC013292.274:11565-11880	4.44273	AT3G03560.1		
MDP0000770800	MDC013293.150:1391-3136	4.58566	AT5G54160.1	ATOMT1,OMT1	O-methyltransferase 1
MDP0000552625	MDC013293.259:4365-6445	1.79769e+308	AT5G54160.1	ATOMT1,OMT1	O-methyltransferase 1
MDP0000251667	MDC013317.553:6374-6890	2.19849	AT3G53030.1	SRPK4	ser/arg-rich protein kinase 4
MDP0000297541	MDC013322.286:4846-5170	-3.45586	AT4G10810.1		
MDP0000299310	MDC013323.310:3368-3941	3.23961	AT5G37740.1		Calcium-dependent lipid-binding (CaLB domain) family protein
MDP0000509613	MDC013334.62:6231-9119	2.82965	AT5G54500.1	FQR1	flavodoxin-like quinone reductase 1
0	MDC013340.296:1896-2851	2.15177	AT5G66900.1		Disease resistance protein (CC-NBS-LRR class) family
MDP0000879254	MDC013370.129:3544-3757	1.79769e+308	AT5G27740.1	EMB161,EMB251,EMB2775,RF	ATPase family associated with various cellular activities (AAA)
MDP0000321244	MDC013391.281:2664-5545	-2.72681	AT2G39730.1	RCA	rubisco activase
MDP0000127750	MDC013396.400:1704-2232	1.79769e+308	AT5G06280.1		
MDP0000781483	MDC013398.12:1975-2220	1.79769e+308	AT5G05780.1	AE3,ATHMOV34,RPN8A	RP non-ATPase subunit 8A
0	MDC013436.578:2-321	4.86954			
MDP0000197294	MDC013447.197:2467-3134	-2.37931	AT1G59590.1	ZCF37	ZCF37
MDP0000330267	MDC013483.319:9906-10138	1.79769e+308	AT1G51720.1		Amino acid dehydrogenase family protein
MDP0000242107	MDC013483.320:693-11036	1.79769e+308	AT3G21295.1		Tudor/PWWP/MBT superfamily protein
MDP0000294034	MDC013503.290:1841-10868	-3.89521	AT5G54490.1	PBP1	pinoid-binding protein 1
MDP0000202103	MDC013530.431:3610-9556	2.12598	AT5G66680.1	DGL1	dolichyl-diphosphooligosaccharide-protein glycosyltransferase 48kDa subunit family protein
0	MDC013531.194:13169-13505	-1.68373	AT3G54820.1	PIP2D, PIP2;5	plasma membrane intrinsic protein 2;5
MDP0000127858	MDC013560.287:995-1943	2.54815	AT1G01550.1	BPS1	Protein of unknown function (DUF793)
MDP0000296782	MDC013694.297:8575-9076	-2.08309	AT1G76100.1	PETE1	plastocyanin 1
MDP0000292511	MDC013737.149:32341-48180	-2.49478	AT1G31280.1	AGO2	Argonaute family protein
MDP0000293269	MDC013748.450:1014-3320	-2.4209	AT1G23740.1		Oxidoreductase, zinc-binding dehydrogenase family protein
0	MDC013772.153:26501-26780	1.79769e+308			
MDP0000292908	MDC013792.490:2156-2412	1.79769e+308	AT1G11300.1		protein serine/threonine kinases;protein kinases;ATP binding;sugar binding;kinases;carbohydrate binding
MDP0000613481	MDC013811.289:959-1532	1.79769e+308	AT3G11820.2	AT-SYR1,ATSYP121,ATSYR1,F	syntaxin of plants 121
0	MDC013818.335:4169-4688	3.36997			
0	MDC013883.433:28953-29243	1.79769e+308	AT2G27450.2	NLP1, ATNLP1, CPA	nitrilase-like protein 1
MDP0000852436	MDC013898.320:3711-4586	1.93167	AT4G14305.1		Peroxisomal membrane 22 kDa (Mpv17/PMP22) family protein
MDP0000801806	MDC013941.232:17328-17757	1.79769e+308	AT2G44310.1		Calcium-binding EF-hand family protein
MDP0000300051	MDC013941.240:13812-14241	1.79769e+308	AT2G44310.1		Calcium-binding EF-hand family protein
0	MDC013941.240:3518-4137	1.79769e+308			
0	MDC013955.368:6283-6564	1.78501			
0	MDC013969.325:6821-6931	1.79769e+308			
MDP0000321180	MDC013970.243:6073-9465	-2.361	AT4G27310.1		B-box type zinc finger family protein
MDP0000328448	MDC013970.243:6073-9465	-2.18489			
0	MDC014001.354:28948-29441	3.47093			
MDP0000199273	MDC014010.337:25823-32224	3.30595	AT1G70710.1	ATGH9B1,CEL1,GH9B1	glycosyl hydrolase 9B1
MDP0000232028	MDC014064.227:18740-19212	1.79769e+308	AT4G14290.1		alpha/beta-Hydrolases superfamily protein
MDP0000299402	MDC014069.193:12100-15622	1.96623	AT5G47770.1	FPS1	farnesyl diphosphate synthase 1
0	MDC014100.85:6143-6452	1.79769e+308			
MDP0000259550	MDC014142.468:6624-9069	-2.19289	AT3G23560.1	ALF5	MATE efflux family protein
0	MDC014145.337:859-1071	1.79769e+308			
MDP0000934638	MDC014183.458:6858-6948	1.79769e+308	AT1G68320.1	AtMYB62,BW62B,BW62C,MYB	myb domain protein 62
MDP0000151848	MDC014188.335:19493-19720	1.79769e+308	AT4G38960.3		B-box type zinc finger family protein
MDP0000151871	MDC014189.230:15027-17751	1.79769e+308	AT5G63140.1	ATPAP29,PAP29	purple acid phosphatase 29
MDP0000380032	MDC015008.179:340-1209	1.79769e+308	AT3G09270.1	ATGSTU8,GSTU8	glutathione S-transferase TAU 8
MDP0000296890	MDC015025.46:19239-23920	4.28703	AT5G64010.1		
0	MDC015077.305:4104-4809	1.79769e+308	AT2G45570.1	CYP76C2	cytochrome P450, family 76, subfamily C, polypeptide 2
MDP0000242554	MDC015092.88:5088-9340	2.73401	AT1G32640.1	ATMYC2,JAI1,JIN1,MYC2,RD2	Basic helix-loop-helix (bHLH) DNA-binding family protein
MDP0000128423	MDC015104.166:2087-5923	2.28599	AT2G40095.1		Alpha/beta hydrolase related protein
MDP0000537361	MDC015142.537:14989-16316	-1.39589	AT1G17890.1	GER2	NAD(P)-binding Rossmann-fold superfamily protein
0	MDC015146.108:31720-32772	-1.98175	AT2G04780.2	FLA7	FASCICLIN-like arabinoogalactan 7
MDP0000141470	MDC015208.187:15605-15861	1.79769e+308	AT1G17440.1	EER4,TAF12B	Transcription initiation factor TFIID subunit A
MDP0000199052	MDC015262.201:26347-28858	1.79769e+308	AT3G12170.1		Chaperone DnaJ-domain superfamily protein
0	MDC015272.259:4201-4401	1.79769e+308	AT3G02050.1	KUP3, ATKUP3, ATKT4	K+ uptake transporter 3
MDP0000886138	MDC015326.176:6704-8094	3.222	AT2G41380.1		S-adenosyl-L-methionine-dependent methyltransferases superfamily protein
MDP0000128562	MDC015329.200:5868-6237	4.48568	AT1G24020.1	MLP423	MLP-like protein 423
MDP0000292846	MDC015334.66:31137-33830	-2.13953	AT3G12650.1		
MDP0000911067	MDC015370.131:7512-9083	2.65117	AT5G36110.1	CYP716A1	cytochrome P450, family 716, subfamily A, polypeptide 1
MDP0000211411	MDC015465.154:6369-6696	1.89734	AT2G46330.1	AGP16,ATAGP16	arabinogalactan protein 16
MDP0000643281	MDC015509.61:3168-14885	1.90115	AT5G50230.1		Transducin/WD40 repeat-like superfamily protein
MDP0000804928	MDC015524.135:32477-32885	2.39618	AT1G29290.1		
MDP0000211726	MDC015555.52:35553-37271	-3.57441	AT3G25570.1		Adenosylmethionine decarboxylase family protein
0	MDC015568.269:465-688	1.79769e+308			
MDP0000740648	MDC015573.110:11769-12270	-2.10837	AT1G76100.1	PETE1	plastocyanin 1
MDP0000361550	MDC015573.110:12297-12405	-2.18377			
MDP0000301453	MDC015614.99:996-8975	2.76432	AT4G36760.1	APP1,ATAPP1	aminopeptidase P1
MDP0000926531	MDC015688.120:16939-18822	1.79769e+308	AT5G55920.1	OLI2	S-adenosyl-L-methionine-dependent methyltransferases superfamily protein
0	MDC015710.110:1669-1846	1.79769e+308			
MDP0000622590	MDC015714.239:27931-31776	2.17217	AT2G41690.1	AT-HSFB3,HSFB3	heat shock transcription factor B3
MDP0000511014	MDC015753.354:8942-11096	-2.69683			
0	MDC015771.323:788-1107	1.79769e+308	AT1G31910.1		GHMP kinase family protein
MDP0000305934	MDC015800.101:15993-16548	-1.96377	AT1G47960.1	ATC/VIF1,C/VIF1	cell wall / vacuolar inhibitor of fructosidase 1
MDP0000304497	MDC015808.251:229-1821	-2.18938	AT5G38760.1		Late embryogenesis abundant protein (LEA) family protein
0	MDC015817.135:987-1875	2.75131			
MDP0000207654	MDC015828.56:17681-17876	1.79769e+308	AT2G20650.1		RING/U-box superfamily protein
MDP0000304369	MDC015831.96:1977-2271	-2.01267	AT5G59320.1	LTP3	lipid transfer protein 3
MDP0000575586	MDC015856.331:10564-10900	1.79769e+308	AT5G13640.1	ATPDAT,PDAT,PDAT1	phospholipid:diacylglycerol acyltransferase
MDP0000205889	MDC015861.207:6372-7099	-3.13665	AT2G46330.1	AGP16,ATAGP16	arabinogalactan protein 16
MDP0000214320	MDC015872.319:12837-13304	4.6241	AT5G42905.1		Polynucleotidyl transferase, ribonuclease H-like superfamily protein
0	MDC015872.319:14910-15332	1.79769e+308			
MDP0000242922	MDC015883.197:6246-9638	2.11224	AT5G25560.2		CHY-type/CTCHY-type/RING-type Zinc finger protein
0	MDC015908.225:5190-5252	1.79769e+308			
MDP0000331516	MDC015921.316:6350-6605	1.79769e+308			
MDP0000301675	MDC015929.425:11804-12965	-1.185			
MDP0000304911	MDC015982.214:20165-23127	1.79769e+308	AT3G26510.1		Octicosapeptide/Phox/Bem1p family protein
MDP0000641053	MDC016006.279:2737-11176	-1.59982	AT5G64260.1	EXL2	EXORDIUM like 2
0	MDC016046.75:14470-14707	1.79769e+308			
MDP0000920394	MDC016054.80:4256-4514	1.8797	AT1G32928.1		
MDP0000140330	MDC016069.201:7237-9538	1.79769e+308	AT2G43290.1	MSS3	Calcium-binding EF-hand family protein

MDP0000230446	MDC016069.206:8663-10840	1.79769e+308	AT5G32440.3		Ubiquitin system component Cue protein
MDP0000307358	MDC016071.225:7688-8061	1.79769e+308	AT5G64940.1	ATATH13,ATH13,ATOSA1,OS/	ABC2 homolog 13
MDP0000603546	MDC016163.86:1232-5672	3.30868	AT4G17880.1		Basic helix-loop-helix (bHLH) DNA-binding family protein
MDP0000150985	MDC016175.155:5520-8899	1.79769e+308	AT1G80420.1	ATXRCC1	BRCT domain-containing DNA repair protein
0	MDC016234.268:18791-19347	1.9365			
0	MDC016234.268:19756-19922	1.79769e+308	AT5G07050.1		nodulin MtN21 /EamA-like transporter family protein
0	MDC016257.132:1244-1326	1.79769e+308			
0	MDC016257.197:16577-16693	2.51199			
MDP0000214905	MDC016257.212:5600-8375	1.79769e+308	AT2G38740.1		Haloacid dehalogenase-like hydrolase (HAD) superfamily protein
MDP0000151417	MDC016323.148:1566-3293	-3.48835	AT3G25570.1		Adenosylmethionine decarboxylase family protein
MDP0000348327	MDC016409.316:14506-15265	-1.85887	AT1G12663.1		
MDP0000332786	MDC016470.67:22850-23923	3.16876	AT5G04200.1	AtMC9,MC9	metacaspase 9
MDP0000332786	MDC016470.67:24078-24292	1.79769e+308	AT5G04200.1	AtMC9,MC9	metacaspase 9
0	MDC016479.97:23574-23802	1.79769e+308			
MDP0000432402	MDC016505.50:1237-1427	1.79769e+308	AT5G38830.1		Cysteinyl-tRNA synthetase, class Ia family protein
MDP0000303139	MDC016572.135:2036-13919	2.64572	AT5G13560.1		
0	MDC016588.101:11842-12103	1.79769e+308			
MDP0000243237	MDC016615.207:310-1511	1.79769e+308	AT5G17820.1		Peroxidase superfamily protein
MDP0000903267	MDC016626.388:1735-2929	-1.99629	AT2G37400.1		Tetratricopeptide repeat (TPR)-like superfamily protein
0	MDC016663.136:1192-1407	1.79769e+308			
MDP0000817718	MDC016670.201:4492-4801	1.79769e+308	AT5G63610.1	ATCDK8,CDKE;1,HEN3	cyclin-dependent kinase E;1
MDP0000206461	MDC016676.357:6049-7681	-2.02641	AT5G50790.1		Nodulin MtN3 family protein
0	MDC016716.178:15966-16452	-1.91239			
0	MDC016737.160:344-544	1.79769e+308			
0	MDC016762.98:221-475	1.79769e+308			
MDP0000213265	MDC016787.195:6612-9680	1.79769e+308	AT1G07960.1	ATPDIL5-1,PDIL5-1	PDI-like 5-1
MDP0000356415	MDC016842.248:4052-4349	1.79769e+308			
MDP0000823528	MDC016855.631:39541-39971	-1.94464	AT4G27360.1		Dynein light chain type 1 family protein
MDP0000828077	MDC016904.89:2263-15477	2.31888	AT4G01470.1	ATTIP1.3,GAMMA-TIP3,TIP1;3	tonoplast intrinsic protein 1;3
MDP0000307237	MDC016920.355:2401-4083	1.84566	AT3G21760.1	HYR1	UDP-Glycosyltransferase superfamily protein
MDP0000389969	MDC016942.263:1081-4165	1.79769e+308	AT4G35260.1	IDH-I,IDH1	isocitrate dehydrogenase 1
MDP0000923628	MDC016948.79:12312-12459	-1.87881	AT3G57062.1		
MDP0000213179	MDC016970.176:123-435	-2.08908	AT5G65730.1	XTH6	xyloglucan endotransglucosylase/hydrolase 6
0	MDC017013.566:1815-2228	1.79769e+308	AT2G45220.1		Plant invertase/pectin methylesterase inhibitor superfamily
0	MDC017013.566:2346-2726	1.79769e+308	AT2G45220.1		Plant invertase/pectin methylesterase inhibitor superfamily
0	MDC017013.566:514-1355	1.79769e+308	AT2G45220.1		Plant invertase/pectin methylesterase inhibitor superfamily
0	MDC017021.252:20334-20684	1.79769e+308			
MDP0000304470	MDC017033.58:322-2820	2.05019	AT3G66654.1		Cyclophilin-like peptidyl-prolyl cis-trans isomerase family protein
MDP0000307402	MDC017035.467:27408-27827	3.77878	AT1G12650.1		
MDP0000234499	MDC017051.235:13603-17570	2.02915	AT5G42260.1	BGLU12	beta glucosidase 12
MDP0000215541	MDC017065.233:1015-1592	1.79769e+308			
MDP0000308097	MDC017091.105:21457-21821	1.79769e+308	AT2G04220.1		Plant protein of unknown function (DUF868)
MDP0000507003	MDC017117.184:9382-10816	2.94928	AT2G25060.1	AtENODL14,ENODL14	early nodulin-like protein 14
0	MDC017130.237:1246-1897	4.57808	AT1G18390.2		Protein kinase superfamily protein
0	MDC017130.237:652-870	1.79769e+308			
MDP0000252890	MDC017137.174:6376-7583	-2.13152	AT5G38410.1		Ribulose bisphosphate carboxylase (small chain) family protein
MDP0000243529	MDC017153.343:11521-14215	-2.09166	AT2G32670.1	ATVAMP725,VAMP725	vesicle-associated membrane protein 725
MDP0000513140	MDC017154.657:12553-12769	1.79769e+308	AT1G51190.1	PLT2	Integrase-type DNA-binding superfamily protein
MDP0000351526	MDC017237.222:14315-14834	-3.5073	AT2G45180.1		Bifunctional inhibitor/lipid-transfer protein/seed storage 2S albumin superfamily protein
MDP0000301189	MDC017251.289:4484-5683	1.79769e+308	AT2G39490.1		F-box family protein
0	MDC017263.315:11030-11368	1.79769e+308			
MDP0000233640	MDC017324.278:8810-20137	7.32266	AT1G06720.1		P-loop containing nucleoside triphosphate hydrolases superfamily protein
MDP0000215301	MDC017325.138:24398-26864	1.79769e+308	AT5G63060.1		Sec14p-like phosphatidylinositol transfer family protein
MDP0000755770	MDC017371.127:20493-20837	1.79769e+308			
MDP0000755770	MDC017371.127:21062-21293	1.79769e+308			
0	MDC017405.92:18343-18890	-1.91427			
MDP0000302905	MDC017443.214:7327-8900	1.79769e+308	AT5G13930.1	ATCHS,CHS,TT4	Chalcone and stilbene synthase family protein
0	MDC017450.136:8789-9313	1.79769e+308	AT3G16340.2	PDR1	pleiotropic drug resistance 1
0	MDC017540.252:44737-45439	1.79769e+308	AT4G20820.1		FAD-binding Berberine family protein
0	MDC017562.409:111-463	2.33993			
MDP0000206473	MDC017564.90:2113-2470	4.62132	AT1G51920.1		
MDP0000935120	MDC017578.59:5707-6257	-2.69309	AT5G27670.1	HTA7	histone H2A 7
MDP0000696497	MDC017597.315:14051-19216	2.53574	AT5G53110.1		RING/U-box superfamily protein
MDP0000214697	MDC017636.116:4199-6265	2.00073	AT2G28690.1		Protein of unknown function (DUF1635)
0	MDC017703.193:3608-3712	1.79769e+308			
MDP0000208899	MDC017766.81:3875-5414	1.79769e+308	AT5G13930.1	ATCHS,CHS,TT4	Chalcone and stilbene synthase family protein
0	MDC017818.70:8691-8779	1.79769e+308			
MDP0000130060	MDC017831.356:6069-8826	1.79769e+308	AT5G54580.1		RNA-binding (RRM/RBD/RNP motifs) family protein
MDP0000208137	MDC017843.86:131-1729	3.05473	AT1G56600.1	AtGolS2,GolS2	galactinol synthase 2
MDP0000492982	MDC017850.196:7405-7687	1.79769e+308	AT3G53000.1	AtPP2-A15,PP2-A15	phloem protein 2-A15
MDP0000305335	MDC017850.205:5561-9559	-1.74673	AT2G21990.1		Protein of unknown function, DUF617
MDP0000243861	MDC017880.286:5763-5960	1.79769e+308	AT2G28930.3	APK1B,PK1B	protein kinase 1B
MDP0000708692	MDC017895.316:14102-16075	1.79769e+308	AT2G38470.1	ATWRKY33,WRKY33	WRKY DNA-binding protein 33
0	MDC017935.299:4662-5932	4.08189	AT1G30700.1		FAD-binding Berberine family protein
0	MDC018035.72:1243-1306	-2.76812			
MDP0000261968	MDC018046.125:72337-81764	3.09387	AT3G07790.1		DGCR14-related
MDP0000306151	MDC018097.432:5006-5477	6.62325	AT2G23270.1		
0	MDC018101.293:13762-13915	1.79769e+308	AT1G10010.1	AAP8, ATAAP8	amino acid permease 8
MDP0000792088	MDC018107.153:10041-12319	2.51392	AT5G13080.1	ATWRKY75,WRKY75	WRKY DNA-binding protein 75
MDP0000302888	MDC018131.78:74830-75020	1.79769e+308	AT2G40730.1		Protein kinase family protein with ARM repeat domain
MDP0000623836	MDC018185.243:10459-11068	2.77536	AT3G21550.1	AtDMP2,DMP2	DUF679 domain membrane protein 2
0	MDC018197.265:11208-11227	1.79769e+308			
MDP0000794439	MDC018238.148:9565-11586	2.03383	AT1G80840.1	ATWRKY40,WRKY40	WRKY DNA-binding protein 40
MDP0000772208	MDC018262.284:13539-14055	1.79769e+308	AT3G22560.1		Acyl-CoA N-acyltransferases (NAT) superfamily protein
MDP0000210067	MDC018268.357:14874-17694	2.03795	AT2G14095.1		
MDP0000442718	MDC018328.126:1463-1767	1.79769e+308	AT4G14145.1		
0	MDC018333.136:493-815	1.79769e+308			
0	MDC018344.87:12861-13103	1.79769e+308			
MDP0000307191	MDC018367.267:12775-13673	4.45646	AT3G20840.1	PLT1	Integrase-type DNA-binding superfamily protein
0	MDC018370.363:15975-16396	1.79769e+308			
0	MDC018385.173:5099-5375	1.79769e+308			
MDP0000307797	MDC018389.342:13517-13812	2.72922	AT1G23880.1		NHL domain-containing protein
MDP0000244125	MDC018407.210:85-6962	1.79769e+308	AT1G01710.1		Acyl-CoA thioesterase family protein
MDP0000130449	MDC018419.403:1052-2613	1.87905	AT5G36110.1	CYP716A1	cytochrome P450, family 716, subfamily A, polypeptide 1
MDP0000689946	MDC018430.130:8323-8938	2.19853	AT5G47220.1	ATERF-2,ATERF2,ERF2	ethylene responsive element binding factor 2
MDP0000152589	MDC018440.197:73746-74343	-3.28078	AT5G52190.1		Sugar isomerase (SIS) family protein
MDP0000341297	MDC018445.308:1656-1818	1.79769e+308			
0	MDC018495.75:656-1593	2.5651	AT2G46150.1		Late embryogenesis abundant (LEA) hydroxyproline-rich glycoprotein family
MDP0000950987	MDC018501.311:3908-4131	1.79769e+308	AT4G03510.1	ATRMA1,RMA1	RING membrane-anchor 1
MDP0000212954	MDC018507.307:11511-11932	1.79769e+308	AT1G70670.1		Caleosin-related family protein
MDP0000789174	MDC018519.270:6935-7074	2.55974	AT1G24020.1	MLP423	MLP-like protein 423
MDP0000569487	MDC018527.183:1987-3563	1.79769e+308			
0	MDC018532.136:875-1203	1.79769e+308	AT4G25340.2	FKBP53	FK506 BINDING PROTEIN 53
MDP0000664781	MDC018583.121:765-1006	1.79769e+308	AT1G72040.1		P-loop containing nucleoside triphosphate hydrolases superfamily protein
MDP0000210867	MDC018599.370:2113-2425	1.79769e+308			
MDP0000260882	MDC018600.265:28896-33820	1.97082			
MDP0000207774	MDC018610.106:7849-8953	-1.84235	AT3G20820.1		Leucine-rich repeat (LRR) family protein
MDP0000300795	MDC018638.210:32468-32798	1.79769e+308	AT1G76710.1	ASHH1,SDG26	SET domain group 26
0	MDC018683.391:5370-5442	1.79769e+308			
MDP0000206239	MDC018755.93:19644-21164	-2.26016	AT2G18840.1		Integral membrane Yip1 family protein
0	MDC018763.284:10-79	1.79769e+308			
MDP0000255006	MDC018763.300:9907-13402	1.79769e+308	AT3G01720.1		
MDP0000302206	MDC018797.53:4472-4733	-2.17361			
0	MDC018803.242:11-340	2.78501			
MDP0000306963	MDC018808.231:3422-3614	1.79769e+308	AT1G09630.1	ATRAB-A2A,ATRAB11C,ATRAI	RAB GTPase 11C
MDP0000523487	MDC018812.251:54-3502	1.93781	AT3G55120.1	A11,CFI,TT5	Chalcone-flavanone isomerase family protein
MDP0000782882	MDC018829.211:2566-3010	2.05088			
0	MDC018858.178:25373-25918	2.18952	AT1G01490.2		Heavy metal transport/detoxification superfamily protein
0	MDC018858.178:4311-4783	1.79769e+308			
MDP0000304278	MDC018864.196:3981-5846	1.79769e+308	AT3G52040.1		
MDP0000130738	MDC018913.57:3010-3796	1.79769e+308	AT1G68450.1		VQ motif-containing protein
MDP0000215667	MDC018916.143:5252-7355	1.79769e+308			
MDP0000210595	MDC018926.465:13230-16290	2.28894	AT1G03110.1		Transducin/WD40 repeat-like superfamily protein
MDP0000304459	MDC018926.465:829-2636	1.49662	AT3G22550.1		Protein of unknown function (DUF581)
MDP0000211459	MDC018944.121:6482-6638	1.79769e+308	AT1G19850.1	ARF5,IAA24,MP	Transcriptional factor B3 family protein / auxin-responsive factor AUX/IAA-related
MDP0000921319	MDC018952.103:18323-18583	-4.80994	AT2G10940.1		Bifunctional inhibitor/lipid-transfer protein/seed storage 2S albumin superfamily protein
MDP0000921319	MDC018952.103:19128-19710	-4.48923	AT2G10940.1		Bifunctional inhibitor/lipid-transfer protein/seed storage 2S albumin superfamily protein
MDP0000210434	MDC018952.103:6545-13697	1.79769e+308	AT5G35430.1		Tetratricopeptide repeat (TPR)-like superfamily protein
MDP0000396298	MDC018995.219:2843-3681	1.80436	AT1G80940.1		
0	MDC019060.82:24775-25363	2.10784	AT1G01490.2		Heavy metal transport/detoxification superfamily protein
MDP0000207192	MDC019069.72:1250-2242	2.34966	AT4G14305.1		Peroxisomal membrane 22 kDa (Mpv17/PMP22) family protein
MDP0000386314	MDC019073.253:5201-5696	3.20804	AT4G25900.1		Galactose mutarotase-like superfamily protein
MDP0000386314	MDC019073.253:5757-13541	1.79769e+308	AT4G25900.1		Galactose mutarotase-like superfamily protein
MDP0000303781	MDC019089.170:15740-41193	2.27879	AT1G15740.1		Leucine-rich repeat family protein
MDP0000253234	MDC019095.61:11627-11992	2.71134	AT5G64420.1		DNA polymerase V family
0	MDC019105.344:1605-2183	1.79769e+308	AT4G23160.1	CRK8	cysteine-rich RLK (RECEPTOR-like protein kinase) 8
MDP0000358789	MDC019115.110:2266-2740	-2.58413			
0	MDC019132.134:222-374	1.79769e+308			
MDP0000365923	MDC019144.167:7606-7891	1.79769e+308	AT5G27430.1		Signal peptidase subunit
MDP0000336695	MDC019148.87:3921-4512	-4.33325	AT4G39250.1	ATRL1,RL1,RSM2	RAD-like 1
0	MDC019150.106:626-852	1.85283			
MDP0000936735	MDC019171.140:19104-20870	1.79769e+308	AT5G27410.2		D-aminoacid aminotransferase-like PLP-dependent enzymes superfamily protein
MDP0000321110	MDC019210.287:8349-16802	1.79769e+308	AT5G06550.1		
MDP0000837139	MDC019255.177:10423-10792	1.79769e+308	AT3G07340.1		basic helix-loop-helix (bHLH) DNA-binding superfamily protein

MDP0000827820	MDC019270.183:15826-16306	4.71788	AT1G24020.1	MLP423	MLP-like protein 423
0	MDC019296.227:19299-19915	-1.86788			
0	MDC019305.85:5690-5943	1.79769e+308			
MDP0000143208	MDC019350.189:9254-9462	1.79769e+308	AT5G08130.2	BIM1	basic helix-loop-helix (bHLH) DNA-binding superfamily protein
MDP0000222184	MDC019388.422:2997-5399	1.79769e+308	AT5G36930.2		Disease resistance protein (TIR-NBS-LRR class) family
MDP0000508081	MDC019411.248:5935-6349	1.79769e+308	AT2G44310.1		Calcium-binding EF-hand family protein
MDP0000696624	MDC019411.260:1762-2191	1.79769e+308	AT2G44310.1		Calcium-binding EF-hand family protein
MDP0000310308	MDC019445.155:6053-6213	1.79769e+308	AT2G06000.1		Pentatricopeptide repeat (PPR) superfamily protein
MDP0000322237	MDC019493.102:3700-11010	1.79769e+308	AT5G54570.1	BGLU41	beta glucosidase 41
MDP0000329499	MDC019532.134:17322-17472	1.79769e+308			
MDP0000880838	MDC019575.15:12756-12778	2.06399	AT1G73740.1		UDP-Glycosyltransferase superfamily protein
MDP0000261679	MDC019580.344:3704-9984	3.62236	AT5G26340.1	ATSTP13,MSS1,STP13	Major facilitator superfamily protein
0	MDC019583.195:2352-2815	2.75015	AT2G37760.1		NAD(P)-linked oxidoreductase superfamily protein
0	MDC019583.195:3051-3246	2.41451	AT2G37770.2		NAD(P)-linked oxidoreductase superfamily protein
MDP0000449901	MDC019585.197:5928-6189	2.48902	AT1G19900.1		glyoxal oxidase-related protein
MDP0000449901	MDC019585.197:6197-7127	1.92507	AT1G19900.1		glyoxal oxidase-related protein
MDP0000315936	MDC019603.257:26665-27463	1.79769e+308	AT4G24990.1	ATGP4	Ubiquitin family protein
MDP0000311438	MDC019656.167:25590-27856	1.79769e+308	AT2G03620.1	MGT3,MRS2-5	magnesium transporter 3
MDP0000218810	MDC019661.283:3325-4682	4.53059	AT4G10490.1		2-oxoglutarate (2OG) and Fe(II)-dependent oxygenase superfamily protein
0	MDC019710.194:14070-14423	2.21243			
MDP0000244775	MDC019719.227:864-1022	1.79769e+308	AT2G19330.1	PIRL6	plant intracellular ras group-related LRR 6
MDP0000230601	MDC019748.161:5809-7542	-2.15226	AT1G19000.1		Homeodomain-like superfamily protein
0	MDC019868.260:1962-2259	1.79769e+308			
MDP0000218699	MDC019885.322:5870-6995	2.2786	AT1G75800.1		Pathogenesis-related thaumatin superfamily protein
MDP0000632432	MDC019999.234:2519-2972	-2.69166	AT4G14890.1		2Fe-2S ferredoxin-like superfamily protein
MDP0000593249	MDC020032.306:1846-8801	-1.28641	AT2G32300.1	UCC1	uclacyanin 1
0	MDC020082.132:384-673	1.79769e+308			
0	MDC020082.224:15608-15824	1.79769e+308	AT1G66950.1	PDR11, ATPDR11	pleiotropic drug resistance 11
0	MDC020082.224:16228-16490	1.79769e+308	AT2G36380.1	PDR6, ATPDR6	pleiotropic drug resistance 6
0	MDC020082.224:16703-17360	5.93328	AT2G36380.1	PDR6, ATPDR6	pleiotropic drug resistance 6
0	MDC020098.119:12808-13292	1.79769e+308	AT3G16340.2	PDR1	pleiotropic drug resistance 1
MDP0000412490	MDC020101.37:12007-12985	-3.44931	AT1G56430.1	ATNAS4,NAS4	nicotianamine synthase 4
MDP0000357039	MDC020112.161:5075-5343	1.79769e+308			
MDP0000705053	MDC020153.98:3860-4787	2.38023	AT1G48320.1		Thioesterase superfamily protein
MDP0000705053	MDC020153.98:5120-5424	1.79769e+308	AT1G48320.1		Thioesterase superfamily protein
MDP0000805832	MDC020185.100:13522-14211	-2.15482	AT5G08050.1		Protein of unknown function (DUF1118)
0	MDC020190.132:475-670	1.79769e+308			
MDP0000312316	MDC020206.60:19611-29042	6.42087	AT1G10510.1	emb2004	RNI-like superfamily protein
0	MDC020206.60:80379-80919	3.66853	AT3G48180.1		unknown protein
MDP0000362305	MDC020215.147:7355-7948	-1.80632	AT2G02100.1	LCR69,PDF2.2	low-molecular-weight cysteine-rich 69
MDP0000253928	MDC020235.551:5712-6463	-1.97256	AT5G64820.1		
MDP0000351308	MDC020252.154:3698-3890	1.79769e+308			
MDP0000745504	MDC020314.68:30613-30835	1.79769e+308	AT1G02630.1		Nucleoside transporter family protein
MDP0000593536	MDC020317.187:1475-2264	3.7415	AT2G36690.1		2-oxoglutarate (2OG) and Fe(II)-dependent oxygenase superfamily protein
MDP0000322725	MDC020357.143:7538-13113	1.98536	AT4G29010.1	AIM1	Enoyl-CoA hydratase/isomerase family
0	MDC020381.113:1718-2064	1.79769e+308			
MDP0000219404	MDC020461.201:992-1634	-3.43143	AT3G22142.1		Bifunctional inhibitor/lipid-transfer protein/seed storage 2S albumin superfamily protein
MDP0000366807	MDC020476.369:154-1663	2.415			
MDP0000920189	MDC020483.205:11374-11570	1.79769e+308	AT3G23150.1	ETR2	Signal transduction histidine kinase, hybrid-type, ethylene sensor
MDP0000356161	MDC020502.79:5346-6108	1.79769e+308	AT3G29970.1		B12D protein
MDP0000356161	MDC020502.79:6418-6639	1.79769e+308	AT3G29970.1		B12D protein
MDP0000392904	MDC020542.316:6274-6781	-1.89038	AT2G25770.1		Polyketide cyclase/dehydrase and lipid transport superfamily protein
MDP0000131822	MDC020582.164:1549-3880	1.79769e+308	AT4G05100.1	AtMYB74,MYB74	myb domain protein 74
MDP0000827665	MDC020641.127:10915-12751	-1.90145	AT5G41080.1		PLC-like phosphodiesterases superfamily protein
MDP0000353639	MDC020660.55:9642-9750	1.79769e+308			
MDP0000831481	MDC020665.117:6027-6473	2.9494	AT5G14280.1		DNA-binding storekeeper protein-related
MDP0000218132	MDC020684.226:11654-14980	1.79769e+308	AT3G15070.1		RING/U-box superfamily protein
MDP0000472203	MDC020837.138:583-9198	2.24942	AT5G18600.1		Thioredoxin superfamily protein
MDP0000311115	MDC020959.82:16235-16669	1.79769e+308	AT5G51270.1		U-box domain-containing protein kinase family protein
0	MDC020997.87:1778-2245	1.79769e+308			
MDP0000215799	MDC021014.157:400-4861	1.79769e+308	AT1G50030.2	TOR	target of rapamycin
0	MDC021041.235:4758-5058	1.79769e+308			
MDP0000143860	MDC021083.97:4312-4666	1.79769e+308	AT4G15475.1		F-box/RNI-like superfamily protein
MDP0000314632	MDC021107.66:19990-21331	-2.88031	AT1G70760.1	CRR23	inorganic carbon transport protein-related
MDP0000217690	MDC021125.222:2795-2956	1.79769e+308	AT2G35980.1	ATNHL10,NHL10,YLS9	Late embryogenesis abundant (LEA) hydroxyproline-rich glycoprotein family
MDP0000758237	MDC021125.354:16615-17911	3.56375	AT5G01750.2		Protein of unknown function (DUF567)
MDP0000312359	MDC021195.261:14143-15009	2.1252	AT2G16060.1	AHB1,ARATH GLB1,ATGLB1,G	hemoglobin 1
0	MDC021221.287:44679-45111	2.02561			
MDP0000312258	MDC021224.329:17770-36527	1.79769e+308	AT1G79000.1	ATHAC1,ATHPCAT2,HAC1,PC	histone acetyltransferase of the CBP family 1
MDP0000312256	MDC021224.329:4725-8465	1.27257	AT5G38630.1	ACYB-1,CYB-1	cytochrome B561-1
MDP0000225981	MDC021229.327:8915-15234	1.79769e+308	AT3G45300.1	ATIVD,IVD,IVDH	isovaleryl-CoA-dehydrogenase
MDP0000313786	MDC021262.50:13957-14057	1.79769e+308	AT3G15880.2	TPR4,WSIP2	WUS-interacting protein 2
MDP0000222305	MDC021270.197:3504-5232	1.79769e+308	AT5G06710.1	HAT14	homeobox from Arabidopsis thaliana
MDP0000221246	MDC021379.127:3832-9589	1.79769e+308	AT1G02840.1	ATSRP34,SR1,SRP34	RNA-binding (RRM/RBD/RNP motifs) family protein
MDP0000332721	MDC021386.436:29757-30095	1.79769e+308			
MDP0000261935	MDC021421.104:11161-11618	2.97142	AT5G27460.1		Tetratricopeptide repeat (TPR)-like superfamily protein
MDP0000217124	MDC021463.474:2997-5056	1.79769e+308	AT1G77930.1		Chaperone DnaJ-domain superfamily protein
MDP0000142814	MDC021508.119:703-1183	2.05401	AT1G24020.1	MLP423	MLP-like protein 423
MDP0000314223	MDC021521.150:23479-23682	-2.38109	AT1G78610.1	MSL6	mechanosensitive channel of small conductance-like 6
MDP0000336734	MDC021527.347:5167-5551	-2.40514			
0	MDC021555.260:3534-3862	1.79769e+308	AT4G20970.1		basic helix-loop-helix (bHLH) DNA-binding superfamily protein
0	MDC021563.286:640-1084	2.21243	AT5G37850.3	SOS4	pfkB-like carbohydrate kinase family protein
MDP0000221871	MDC021563.301:16401-23355	2.03305	AT5G37850.1	ATSOS4,SOS4	pfkB-like carbohydrate kinase family protein
0	MDC021594.146:14-493	4.97147			
MDP0000315449	MDC021648.178:15444-17993	1.79769e+308	AT2G26070.1	RTE1	Protein of unknown function (DUF778)
0	MDC021658.157:9449-9957	-2.16656	AT1G60950.1	FED A, ATFD2	2Fe-2S ferredoxin-like superfamily protein
MDP0000392201	MDC021684.90:873-4447	-2.20956	AT3G20820.1		Leucine-rich repeat (LRR) family protein
MDP0000313454	MDC021689.441:3963-4557	5.51402	AT1G24020.1	MLP423	MLP-like protein 423
MDP0000312569	MDC021689.443:9371-9851	4.3114	AT1G24020.1	MLP423	MLP-like protein 423
MDP0000216907	MDC021689.444:11887-12520	4.4464	AT1G24020.1	MLP423	MLP-like protein 423
MDP0000500806	MDC021689.444:7604-11877	1.79769e+308	AT1G24020.1	MLP423	MLP-like protein 423
MDP0000576126	MDC021691.154:2961-7337	-3.79609	AT5G38020.1		S-adenosyl-L-methionine-dependent methyltransferases superfamily protein
MDP0000314777	MDC021716.303:5439-6015	3.25986	AT3G21550.1	AtDMP2,DMP2	DUF679 domain membrane protein 2
MDP0000313600	MDC021763.143:53-1175	2.44885	AT4G37300.1	MEE59	maternal effect embryo arrest 59
MDP0000764876	MDC021795.40:6148-6790	-3.43143	AT3G22142.1		Bifunctional inhibitor/lipid-transfer protein/seed storage 2S albumin superfamily protein
MDP0000542944	MDC021813.304:19401-20097	-1.88806	AT3G22840.1	ELIP,ELIP1	Chlorophyll A-B binding family protein
MDP0000132431	MDC021842.102:1393-3748	-2.43746	AT4G03210.1	XTH9	xyloglucan endotransglucosylase/hydrolase 9
MDP0000378203	MDC021843.194:2520-4873	-2.1428	AT4G03210.1	XTH9	xyloglucan endotransglucosylase/hydrolase 9
MDP0000132456	MDC021864.218:7479-8925	1.89573	AT5G42650.1	AOS,CYP74A,DDE2	allene oxide synthase
MDP0000574556	MDC021880.120:18168-18768	2.77916	AT5G39670.1		Calcium-binding EF-hand family protein
MDP0000142675	MDC021907.185:4850-5618	1.79769e+308			
MDP0000361589	MDC021912.336:80015-80513	-2.59832	AT3G45980.1	H2B,HTB9	Histone superfamily protein
MDP0000309694	MDC021925.357:9645-18947	2.03349	AT4G34640.1	ERG9,SQS1	squalene synthase 1
MDP0000312665	MDC021956.140:17630-30598	1.79769e+308	AT5G06600.1	UBP12	ubiquitin-specific protease 12
MDP0000701077	MDC022002.79:2723-4463	-2.10433	AT1G05850.1	ATCTL1,CTL1,ELP,ELP1,ERH2	Chitinase family protein
MDP0000245720	MDC022027.142:3660-5484	1.79769e+308	AT1G52800.1		2-oxoglutarate (2OG) and Fe(II)-dependent oxygenase superfamily protein
MDP0000261346	MDC022047.116:7935-11907	-3.22425	AT2G28900.1	ATOEP16-1,ATOEP16-L,OEP1(	outer plastid envelope protein 16-1
MDP0000219282	MDC022049.136:3911-5363	-3.07805	AT3G16520.3	UGT88A1	UDP-glucosyl transferase 88A1
MDP0000216076	MDC022049.144:1863-2445	-2.6847	AT3G16520.3	UGT88A1	UDP-glucosyl transferase 88A1
0	MDC022084.259:9269-9540	1.79769e+308			
0	MDC022115.179:2130-2784	1.76737	AT5G06320.1	NHL3	NDR1/HIN1-like 3
MDP0000245757	MDC022126.96:1332-1713	3.46619	AT1G72960.1		Root hair defective 3 GTP-binding protein (RHD3)
MDP0000309512	MDC022131.36:462-5980	1.79769e+308	AT5G03290.1	IDH-V	isocitrate dehydrogenase V
MDP0000388769	MDC022151.329:5891-9309	2.43417	AT2G37040.1	ATPAL1,PAL1	PHE ammonia lyase 1
MDP0000132621	MDC022159.201:1198-1649	4.00098	AT5G43580.1		Serine protease inhibitor, potato inhibitor I-type family protein
MDP0000699845	MDC022200.129:11651-13693	1.90489			
MDP0000309382	MDC022203.80:8540-9550	2.21842	AT5G13180.1	ANAC083,NAC083,VNI2	NAC domain containing protein 83
MDP0000223410	MDC022295.98:32919-34391	1.79769e+308	AT5G25170.1		PPPDE putative thiol peptidase family protein
MDP0000940098	MDC022297.232:13220-15870	-2.69487	AT3G11910.1	UBP13	ubiquitin-specific protease 13
MDP0000328060	MDC022301.307:90-353	1.79769e+308	AT5G61510.1		GroES-like zinc-binding alcohol dehydrogenase family protein
0	MDC022363.241:6084-6289	1.79769e+308			
0	MDC022366.63:24207-24828	3.6344	AT5G13220.4		jasmonate-zim-domain protein 10
0	MDC022366.63:25851-26449	3.55241			
MDP0000366309	MDC022435.108:200-507	1.79769e+308	AT1G05120.1		Helicase protein with RING/U-box domain
MDP0000372061	MDC022441.31:99-976	2.95152	AT5G66170.3	STR18	sulfurtransferase 18
MDP0000370937	MDC022441.35:130-1007	3.18225	AT5G66170.3	STR18	sulfurtransferase 18
MDP0000094255	MDC022474.98:3860-12957	-2.186	AT1G04820.1	TOR2,TUA4	tubulin alpha-4 chain
MDP0000094255	MDC022474.98:3860-12957	-2.1253	AT1G04820.1	TOR2,TUA4	tubulin alpha-4 chain
MDP0000656154	MDC022487.75:10821-11616	-1.97446	AT2G34430.1	LHB1B1,LHCB1.4	light-harvesting chlorophyll-protein complex II subunit B1
MDP0000656152	MDC022487.75:8161-8959	-1.86434	AT1G29930.1	AB140,CAB1,CAB140,LHCB1.3	chlorophyll A/B binding protein 1
0	MDC022531.114:4018-4893	3.36846	AT2G46150.1		Late embryogenesis abundant (LEA) hydroxyproline-rich glycoprotein family
0	MDC022564.61:3605-3774	1.79769e+308			
MDP0000591411	MDC022569.33:5324-5484	-3.41737	AT3G63380.1		ATPase E1-E2 type family protein / haloacid dehalogenase-like hydrolase family protein
MDP0000805894	MDC022597.70:34733-36744	-2.17549	AT1G11860.1		Glycine cleavage T-protein family
MDP0000465139	MDC022631.45:303-972	1.79769e+308	AT5G65310.1	ATHB-5,ATHB5,HB5	homeobox protein 5
MDP0000315498	MDC022702.107:16118-17225	-3.11467	AT3G12610.1	DRT100	Leucine-rich repeat (LRR) family protein
MDP0000092978	MDC022773.244:7871-8075	1.79769e+308	AT5G65290.1		LMBR1-like membrane protein
0	MDC022780.65:594-837	2.55647			
MDP0000132952	MDC022792.139:8367-9744	1.79769e+308	AT5G47980.1		HXXXD-type acyl-transferase family protein
MDP0000246042	MDC022803.121:4647-6548	1.79769e+308	AT3G17600.1	IAA31	indole-3-acetic acid inducible 31
MDP0000254473	MDC022821.76:29441-58233	1.9116	AT4G33680.1	AGD2	Pyridoxal phosphate (PLP)-dependent transferases superfamily protein
MDP0000225360	MDC022824.304:3660-3829	1.79769e+308	AT4G26140.1	BGAL12	beta-galactosidase 12
MDP0000225483	MDC022839.109:16313-16652	1.79769e+308	AT3G03860.1	APRL5,ATAPRL5	APR-like 5
0	MDC022879.56:1375-1884	1.79769e+308	AT2G22420.1		Peroxidase superfamily protein

MDP0000308875	MDC022879.64:8443-8780	1.79769e+308	AT5G10360.1	EMB3010,RPS6B	Ribosomal protein S6e
0	MDC022894.374:9989-10324	1.79769e+308			
MDP0000145050	MDC023085.39:1191-3316	1.79769e+308	AT2G31180.1	ATMYB14,MYB14,MYB14AT	myb domain protein 14
MDP0000154734	MDC023147.45:4964-7678	2.37369	AT5G13080.1	ATWRKY75,WRKY75	WRKY DNA-binding protein 75
MDP0000144280	MDC023306.53:6102-7273	1.79769e+308	AT1G35210.1		
MDP0000344130	MDC023311.35:7749-8338	1.72265			
MDP0000655939	MDC023492.75:33-679	3.53624	AT3G54420.1	ATCHITIV,ATEP3,CHIV,EP3	homolog of carrot EP3-3 chitinase
MDP0000310109	MDC023507.55:3458-7466	2.57308	AT4G01560.1	MEE49	Ribosomal RNA processing Brix domain protein
MDP0000824468	MDC023572.35:3291-4882	1.79769e+308	AT1G02120.1	VAD1	GRAM domain family protein
0	MDC023576.93:1279-1667	-2.75104			
MDP0000769652	MDC023607.46:9470-10046	4.48219	AT2G46150.1		Late embryogenesis abundant (LEA) hydroxyproline-rich glycoprotein family
MDP0000229468	MDC023757.22:743-2304	2.00331	AT5G36110.1	CYP716A1	cytochrome P450, family 716, subfamily A, polypeptide 1
MDP0000767063	MDC023849.16:6797-7283	1.79769e+308	AT1G35210.1		
MDP0000390049	MDC023913.71:5802-6066	2.65704	AT1G34060.1		Pyridoxal phosphate (PLP)-dependent transferases superfamily protein
MDP0000254078	MDC024051.25:30601-30826	1.79769e+308	AT5G63080.1		2-oxoglutarate (2OG) and Fe(II)-dependent oxygenase superfamily protein
MDP0000316310	MDC024251.13:2237-3536	2.97323	AT1G78860.1		D-mannose binding lectin protein with Apple-like carbohydrate-binding domain
MDP0000254057	MDC024467.18:10070-10976	1.79769e+308	AT2G27770.1		Plant protein of unknown function (DUF868)
0	MDC024785.32:20131-20658	1.79769e+308			
MDP0000229958	MDC025025.28:2836-3810	-4.49642	AT5G15230.1	GASA4	GAST1 protein homolog 4
MDP0000226405	MDC025032.16:6902-7125	1.79769e+308	AT1G02400.1	ATGA2OX4,ATGA2OX6,DTA1,(	gibberellin 2-oxidase 6
MDP0000316472	MDC025128.19:896-2663	1.79769e+308			
0	MDC025179.21:23-403	1.79769e+308			
MDP0000226193	MDC025338.53:10778-16491	1.79769e+308	AT5G50400.1	ATPAP27,PAP27	purple acid phosphatase 27
0	MDC025463.22:3749-4156	-3.07297			
MDP0000318256	MDC025619.11:2890-10293	1.79769e+308	AT1G73050.1		Glucose-methanol-choline (GMC) oxidoreductase family protein
MDP0000318069	MDC025702.20:9270-10841	2.65117	AT5G36110.1	CYP716A1	cytochrome P450, family 716, subfamily A, polypeptide 1
0	MDC026144.28:2897-3346	-1.97344			
MDP0000229338	MDC026688.7:88-304	2.13927	AT5G36110.1	CYP716A1	cytochrome P450, family 716, subfamily A, polypeptide 1
MDP0000316181	MDC027100.17:2364-7236	1.79769e+308	AT1G51740.1	ATSYP81,ATUFE1,SYP81,UFE	syntaxin of plants 81
MDP0000133520	MDC027330.53:6528-8747	1.79769e+308	AT2G26560.1	PLA IIA,PLA2A,PLP2	phospholipase A 2A
MDP0000228673	MDC027343.22:8537-10521	-1.88286	AT5G25610.1	ATRD22,RD22	BURP domain-containing protein
0	MDC027439.4:1106-2173	1.79769e+308			
0	MDC027586.25:17627-18649	3.74147	AT2G46150.1		Late embryogenesis abundant (LEA) hydroxyproline-rich glycoprotein family
0	MDC027586.25:9596-10534	3.70806	AT3G54200.1		Late embryogenesis abundant (LEA) hydroxyproline-rich glycoprotein family
MDP0000229796	MDC028034.30:3913-5269	1.79769e+308	AT4G10490.1		2-oxoglutarate (2OG) and Fe(II)-dependent oxygenase superfamily protein
MDP0000317816	MDC029025.16:37724-39632	-2.22916	AT5G01600.1	ATFER1,FER1	ferretin 1
MDP0000227827	MDC029110.8:34469-35629	-2.14091	AT2G03550.1		alpha/beta-Hydrolases superfamily protein
MDP0000246508	MDC029331.22:370-647	1.85922			
MDP0000228456	MDC029335.25:6007-7048	2.82399	AT2G15760.1		Protein of unknown function (DUF1645)
0	MDC029401.43:2619-2642	1.79769e+308			
0	MDC029428.27:19524-19555	1.79769e+308			
MDP0000357899	MDC029522.47:22578-23050	1.79769e+308			
MDP0000390563	MDC029522.47:26456-26678	1.79769e+308			
0	MDC029522.47:29375-29799	1.79769e+308			
MDP0000228257	MDC029541.17:30026-31667	-2.98044	AT5G15240.1		Transmembrane amino acid transporter family protein
MDP0000782085	MDC029683.23:9115-9368	4.22898	AT3G04720.1	HEL,PR-4,PR4	pathogenesis-related 4
0	MDC029690.2:317-603	1.79769e+308			
0	MDC031150.12:8267-8723	1.79769e+308	AT4G18160.1	KCO6, ATTPK3, ATKCO6, TPK	Ca2+ activated outward rectifying K+ channel 6
MDP0000910895	MDC031261.10:30690-31071	1.79769e+308	AT5G11970.1		Protein of unknown function (DUF3511)
MDP0000316468	MDC032201.3:1004-1193	1.79769e+308			
MDP0000227463	MDC032660.9:5945-14498	6.5024	AT2G21440.1		RNA-binding (RRM/RBD/RNP motifs) family protein
MDP0000705797	MDC034940.5:2269-7814	-2.70526	AT4G30430.1	TET9	tetraspanin9
MDP0000595200	MDC035405.21:18653-18905	1.79769e+308	AT1G17810.1	BETA-TIP	beta-tonoplast intrinsic protein
MDP0000661371	MDC035507.15:17028-17370	-2.3258	AT5G59320.1	LTP3	lipid transfer protein 3
MDP0000228304	MDC035519.7:9734-13224	4.85453	AT3G56400.1	ATWRKY70,WRKY70	WRKY DNA-binding protein 70
MDP0000761113	MDC035533.8:11708-13073	2.77336	AT2G14095.1		
MDP0000143463	MDC035751.16:12042-12657	4.53253	AT4G37290.1		
MDP0000143462	MDC035751.16:9288-9911	4.88732	AT4G37290.1		
0	MDC036102.13:6186-6377	1.79769e+308			
0	MDC036190.10:2148-2600	4.37825			
MDP0000859897	MDC037018.11:5057-5594	1.79769e+308	AT2G45760.1	BAL,BAP2	BON association protein 2
MDP0000478473	MDC037119.7:1598-3986	1.97805	AT5G36110.1	CYP716A1	cytochrome P450, family 716, subfamily A, polypeptide 1
0	MDC037361.9:6784-7143	1.79769e+308			
MDP0000836051	MDC037826.18:47629-48283	3.29594	AT2G46150.1		Late embryogenesis abundant (LEA) hydroxyproline-rich glycoprotein family
0	MDC038019.6:878-1176	1.79769e+308			
MDP0000739955	MDC038611.7:8913-9138	4.66013			
MDP0000739957	MDC038611.7:9423-17275	1.79769e+308			
MDP0000229364	MDC039821.7:261-870	3.78343	AT1G24020.1	MLP423	MLP-like protein 423
0	MDC039927.8:6532-7016	1.79769e+308	AT5G45230.1		Disease resistance protein (TIR-NBS-LRR class) family
MDP0000317158	MDC039977.12:10248-22411	-1.92458	AT2G42990.1		GDSL-like Lipase/Acylhydrolase superfamily protein
MDP0000627178	MDC039977.12:10248-22411	2.53116	AT3G11660.1	NHL1	NDR1/HIN1-like 1
MDP0000246775	MDC040311.10:4864-6142	1.95053	AT1G75800.1		Pathogenesis-related thaumatin superfamily protein
MDP0000635659	MDC041102.8:8881-9496	2.0994	AT1G17860.1		Kunitz family trypsin and protease inhibitor protein
MDP0000134064	MDC042250.3:3997-5643	1.79769e+308			
MDP0000345608	MDC043994.10:1318-1504	1.79769e+308			

Supplementary Table 2 **BLASTx results of the significantly differentially expressed novel ORFs in *M*. x *domestica* in response to fire blight infection.**

***M. domestica* locus**	***A. thaliana* accession**	***A. thaliana* name**	**Description**
MDC000127.652:4251-4492	AT4G33720.1		CAP (Cysteine-rich secretory proteins, Antigen 5, and Pathogenesis-related 1 protein) superfamily protein
MDC000446.497:2613-3451	AT3G19615.1		unknown protein
MDC000455.144:3026-3468	AT4G24620.1	PGI1, PGI	phosphoglucose isomerase 1
MDC001441.272:13692-14163	AT5G43920.1		transducin family protein / WD-40 repeat family protein
MDC001471.424:93507-93619	AT2G29260.1		NAD(P)-binding Rossmann-fold superfamily protein
MDC001671.165:2805-2973	AT1G78380.1	ATGSTU19, GST8, GSTU19	glutathione S-transferase TAU 19
MDC001963.417:4816-4925	AT4G27250.2		NAD(P)-binding Rossmann-fold superfamily protein
MDC002322.251:26803-27928	AT4G11280.1	ACS6, ATACS6	1-aminocyclopropane-1-carboxylic acid (acc) synthase 6
MDC002431.300:11772-11965	AT5G19130.2		GPI transamidase component family protein / Gaa1-like family protein
MDC002799.293:2023-2423	AT2G29330.1	TRI	tropinone reductase
MDC003306.225:3010-3406	AT2G16850.1	PIP3B, PIP2;8	plasma membrane intrinsic protein 2;8
MDC003716.306:46958-47066	AT1G03260.1		SNARE associated Golgi protein family
MDC004097.232:8798-9675	AT2G46150.1		Late embryogenesis abundant (LEA) hydroxyproline-rich glycoprotein family
MDC004475.200:15597-16341	AT2G46150.1		Late embryogenesis abundant (LEA) hydroxyproline-rich glycoprotein family
MDC005490.240:8153-8416	AT5G66180.3		S-adenosyl-L-methionine-dependent methyltransferases superfamily protein
MDC005927.393:5902-6584	AT5G10830.1		S-adenosyl-L-methionine-dependent methyltransferases superfamily protein
MDC006260.462:8079-8672	AT1G23440.1		Peptidase C15, pyroglutamyl peptidase I-like
MDC006289.408:7320-7584	AT2G45960.3	PIP1B, TMP-A, ATHH2, PIP1;2	plasma membrane intrinsic protein 1B
MDC006603.734:2300-2410	AT5G39110.1		RmlC-like cupins superfamily protein
MDC007088.513:4786-5156	AT5G24810.2		ABC1 family protein
MDC007581.595:2473-2576	AT4G28460.1		unknown protein
MDC007779.587:2989-3343	AT3G14630.1	CYP72A14	cytochrome P450, family 72, subfamily A, polypeptide 14
MDC007779.587:3489-4308	AT3G14680.1	CYP72A9	cytochrome P450, family 72, subfamily A, polypeptide 9
MDC008017.329:6473-7175	AT2G46150.1		Late embryogenesis abundant (LEA) hydroxyproline-rich glycoprotein family
MDC008031.166:8984-9384	AT3G60030.1	SPL12	squamosa promoter-binding protein-like 12
MDC008212.493:3616-4058	AT5G43180.1		Protein of unknown function, DUF599
MDC008216.318:26921-27156	AT4G20820.1		FAD-binding Berberine family protein
MDC008563.271:10114-10663	AT1G75620.1		glyoxal oxidase-related protein
MDC009139.374:7848-8934	AT2G46150.1		Late embryogenesis abundant (LEA) hydroxyproline-rich glycoprotein family
MDC009630.367:24050-24262	AT2G03810.4		18S pre-ribosomal assembly protein gar2-related
MDC009830.388:3008-4348	AT5G13930.1	CHS, TT4, ATCHS	Chalcone and stilbene synthase family protein
MDC010174.397:1163-1411	AT2G46150.1		Late embryogenesis abundant (LEA) hydroxyproline-rich glycoprotein family
MDC010474.340:3456-4185	AT2G46150.1		Late embryogenesis abundant (LEA) hydroxyproline-rich glycoprotein family
MDC010476.238:18498-19659	AT2G46150.1		Late embryogenesis abundant (LEA) hydroxyproline-rich glycoprotein family
MDC010912.583:5714-6300	AT2G36380.1	PDR6, ATPDR6	pleiotropic drug resistance 6
MDC010981.245:18382-19274	AT3G61510.1	ACS1, AT-ACS1	ACC synthase 1
MDC011449.97:11886-12570	AT2G18680.1		unknown protein
MDC011459.391:12253-12872	AT2G39210.1		Major facilitator superfamily protein
MDC011603.242:16362-17137	AT4G33720.1		CAP (Cysteine-rich secretory proteins, Antigen 5, and Pathogenesis-related 1 protein) superfamily protein
MDC011770.416:7739-8412	AT2G46150.1		Late embryogenesis abundant (LEA) hydroxyproline-rich glycoprotein family
MDC011773.84:2484-3065	AT2G23270.1		unknown protein
MDC012392.481:2083-2367	AT2G29420.1	ATGSTU7, GST25, GSTU7	glutathione S-transferase tau 7
MDC012392.481:2550-3061	AT3G09270.1	ATGSTU8, GSTU8	glutathione S-transferase TAU 8
MDC012733.121:11561-12529	AT4G11280.1	ACS6, ATACS6	1-aminocyclopropane-1-carboxylic acid (acc) synthase 6
MDC012900.215:12407-12925	AT5G01710.1		methyltransferases
MDC013340.296:1896-2851	AT5G66900.1		Disease resistance protein (CC-NBS-LRR class) family
MDC013531.194:13169-13505	AT3G54820.1	PIP2D, PIP2;5	plasma membrane intrinsic protein 2;5
MDC013883.433:28953-29243	AT2G27450.2	NLP1, ATNLP1, CPA	nitrilase-like protein 1
MDC015077.305:4104-4809	AT2G45570.1	CYP76C2	cytochrome P450, family 76, subfamily C, polypeptide 2
MDC015146.108:31720-32772	AT2G04780.2	FLA7	FASCICLIN-like arabinoogalactan 7
MDC015272.259:4201-4401	AT3G02050.1	KUP3, ATKUP3, ATKT4	K+ uptake transporter 3
MDC015771.323:788-1107	AT1G31910.1		GHMP kinase family protein
MDC016234.268:19756-19922	AT5G07050.1		nodulin MtN21 /EamA-like transporter family protein
MDC017013.566:1815-2228	AT2G45220.1		Plant invertase/pectin methylesterase inhibitor superfamily
MDC017013.566:2346-2726	AT2G45220.1		Plant invertase/pectin methylesterase inhibitor superfamily
MDC017013.566:514-1355	AT2G45220.1		Plant invertase/pectin methylesterase inhibitor superfamily
MDC017130.237:1246-1897	AT1G18390.2		Protein kinase superfamily protein
MDC017450.136:8789-9313	AT3G16340.2	PDR1	pleiotropic drug resistance 1
MDC017540.252:44737-45439	AT4G20820.1		FAD-binding Berberine family protein
MDC017935.299:4662-5932	AT1G30700.1		FAD-binding Berberine family protein
MDC018101.293:13762-13915	AT1G10010.1	AAP8, ATAAP8	amino acid permease 8
MDC018495.75:656-1593	AT2G46150.1		Late embryogenesis abundant (LEA) hydroxyproline-rich glycoprotein family
MDC018532.136:875-1203	AT4G25340.2	FKBP53	FK506 BINDING PROTEIN 53
MDC018858.178:25373-25918	AT1G01490.2		Heavy metal transport/detoxification superfamily protein
MDC019060.82:24775-25363	AT1G01490.2		Heavy metal transport/detoxification superfamily protein
MDC019105.344:1605-2183	AT4G23160.1	CRK8	cysteine-rich RLK (RECEPTOR-like protein kinase) 8
MDC019583.195:2352-2815	AT2G37760.1		NAD(P)-linked oxidoreductase superfamily protein
MDC019583.195:3051-3246	AT2G37770.2		NAD(P)-linked oxidoreductase superfamily protein
MDC020082.224:15608-15824	AT1G66950.1	PDR11, ATPDR11	pleiotropic drug resistance 11
MDC020082.224:16228-16490	AT2G36380.1	PDR6, ATPDR6	pleiotropic drug resistance 6
MDC020082.224:16703-17360	AT2G36380.1	PDR6, ATPDR6	pleiotropic drug resistance 6
MDC020098.119:12808-13292	AT3G16340.2	PDR1	pleiotropic drug resistance 1
MDC020206.60:80379-80919	AT3G48180.1		unknown protein
MDC021555.260:3534-3862	AT4G20970.1		basic helix-loop-helix (bHLH) DNA-binding superfamily protein
MDC021563.286:640-1084	AT5G37850.3	SOS4	pfkB-like carbohydrate kinase family protein
MDC021658.157:9449-9957	AT1G60950.1	FED A, ATFD2	2Fe-2S ferredoxin-like superfamily protein
MDC022115.179:2130-2784	AT5G06320.1	NHL3	NDR1/HIN1-like 3
MDC022366.63:24207-24828	AT5G13220.4		jasmonate-zim-domain protein 10
MDC022531.114:4018-4893	AT2G46150.1		Late embryogenesis abundant (LEA) hydroxyproline-rich glycoprotein family
MDC022879.56:1375-1884	AT2G22420.1		Peroxidase superfamily protein
MDC027586.25:17627-18649	AT2G46150.1		Late embryogenesis abundant (LEA) hydroxyproline-rich glycoprotein family
MDC027586.25:9596-10534	AT3G54200.1		Late embryogenesis abundant (LEA) hydroxyproline-rich glycoprotein family
MDC031150.12:8267-8723	AT4G18160.1	KCO6, ATTPK3, ATKCO6, TPK3	Ca2+ activated outward rectifying K+ channel 6
MDC039927.8:6532-7016	AT5G45230.1		Disease resistance protein (TIR-NBS-LRR class) family

Supplementary Table 3 **BLASTx results of all expressed (non-significant) novel ORFs in *M*. x *domestica*.**

***M. domestica* locus**	***A. thaliana* accession**	**PFAM**	**Panther**	**Clusters of c**	**Enzyme cod**	**KEGG orthology**	**Description**
MDC000047.154:15534-16428	AT5G10840.1	PF02990	PTHR10766	KOG1278			transmembrane 9 superfamily member, putative, expressed
MDC001087.439:6147-6450	AT5G20790.2						
MDC011504.316:3604-3768	AT5G56670.1	PF04758	PTHR12650	KOG0009		K02983	expressed protein
MDC011558.545:2913-3398	AT4G38840.1	PF02519				K14488	OsSAUR31 - Auxin-responsive SAUR gene family member, expressed
MDC011558.545:10592-11117	AT4G38840.1	PF02519				K14488	OsSAUR31 - Auxin-responsive SAUR gene family member, expressed
MDC011670.126:374-634	AT2G44620.1	PF00550	PTHR20863	KOG1748	1.6.5.3,1.6.99	K03955	acyl carrier protein, putative, expressed
MDC011700.243:1227-1605	AT5G08139.1	PF00097	PTHR22763	KOG1493			zinc finger, C3HC4 type domain containing protein, expressed
MDC011702.243:1833-2070	AT2G37660.1		PTHR14194	KOG1203			NAD dependent epimerase/dehydratase family protein, putative, expressed
MDC011702.243:3359-3717	AT2G37660.1		PTHR14194	KOG1203			NAD dependent epimerase/dehydratase family protein, putative, expressed
MDC011704.133:1724-2278	AT5G20680.3	PF03005					expressed protein
MDC011727.163:5647-5848	AT3G04730.1	PF02309					OsIAA30 - Auxin-responsive Aux/IAA gene family member, expressed
MDC011727.163:7232-7693	AT3G04730.1	PF02309					OsIAA30 - Auxin-responsive Aux/IAA gene family member, expressed
MDC011766.689:5483-5959	AT2G20142.1	PF01582					expressed protein
MDC011768.383:32501-33098	AT3G23920.1	PF01373					beta-amylase, putative, expressed
MDC011804.393:6327-6784	AT3G51670.1	PF03765,PF00650	PTHR23324	KOG1471			patellin protein, putative, expressed
MDC011809.297:12013-12383	AT3G44280.1						expressed protein
MDC011970.92:16025-16891	AT3G13920.4	PF00270,PF00271	PTHR10967	KOG0328			DEAD-box ATP-dependent RNA helicase, putative, expressed
MDC011970.92:17409-17837	AT1G54270.2	PF00270,PF00271	PTHR10967	KOG0327		K03257	DEAD-box ATP-dependent RNA helicase, putative, expressed
MDC012057.108:18172-18502	AT1G11170.1	PF05212					lysine ketoglutarate reductase trans-splicing related 1, putative, expressed
MDC012065.78:5672-5927	AT1G79040.1	PF04725				K03541	photosystem II 10 kDa polypeptide, chloroplast precursor, putative, expressed
MDC012139.232:7565-8258	AT1G73100.1	PF02182,PF05033,PF00856	PTHR22884	KOG1082			histone-lysine N-methyltransferase, H3 lysine-9 specific SUVH1, putative, expressed
MDC001187.272:8633-8985	AT1G36050.2	PF07970	PTHR10984	KOG2667			endoplasmic reticulum-Golgi intermediate compartment protein 3, putative, expressed
MDC012149.217:8136-8679	AT1G48830.2	PF01251	PTHR11278	KOG3320			40S ribosomal protein S7, putative
MDC012246.289:9924-10344	AT2G30710.1	PF00566	PTHR22957	KOG1092			TBC domain containing protein, expressed
MDC012278.208:17918-18313	AT5G62790.2	PF02670,PF08436			1.1.1.267	K00099	1-deoxy-D-xylulose 5-phosphate reductoisomerase, chloroplast precursor, putative, expressed
MDC012291.307:22481-23638	AT2G16365.4	PF00646					expressed protein
MDC012304.216:7950-8423	AT1G05660.1	PF00295					polygalacturonase, putative, expressed
MDC012304.216:8670-9266	AT1G02790.1	PF00295			3.2.1.67	K01213	polygalacturonase, putative, expressed
MDC012329.239:6033-6692	AT5G05340.1	PF00141			1.11.1.7	K00430	peroxidase precursor, putative, expressed
MDC001207.483:41049-41732	AT1G16560.4	PF04080	PTHR13148				per1-like family protein, putative, expressed
MDC012329.239:7209-7460	AT5G58390.1	PF00141			1.11.1.7	K00430	peroxidase precursor, putative, expressed
MDC012329.239:7574-7771	AT5G58390.1	PF00141			1.11.1.7	K00430	peroxidase precursor, putative, expressed
MDC012329.239:8539-8816	AT5G05340.1	PF00141			1.11.1.7	K00430	peroxidase precursor, putative, expressed
MDC012329.239:18042-18170	AT2G30330.1	PF06320	PTHR13073	KOG3390			expressed protein
MDC012329.239:18286-18476	AT2G30330.1	PF06320	PTHR13073	KOG3390			expressed protein
MDC012392.517:8036-8280	AT1G78380.1	PF02798,PF00043	PTHR11260	KOG0406			glutathione S-transferase, putative, expressed
MDC012438.121:1208-1390	AT4G29260.1	PF03767					HAD superfamily phosphatase, putative, expressed
MDC012447.123:2145-2841	AT4G01370.1	PF00069	PTHR11295	KOG0660	2.7.11.24	K04371	CGMC_MAPKCMGC_2_ERK.14 - CGMC includes CDA, MAPK, GSK3, and CLKC kinases, expressed
MDC012491.393:4-602	AT3G14840.2	PF00560,PF11721,PF07714,PF00069	PTHR23258	KOG1187			receptor-like protein kinase 2, putative, expressed
MDC012491.394:7041-7535	AT3G14840.2	PF00560,PF11721,PF07714,PF00069	PTHR23258	KOG1187			receptor-like protein kinase 2, putative, expressed
MDC012594.475:10716-11062	AT4G04630.1	PF04520					DUF584 domain containing protein, putative, expressed
MDC012645.61:1423-1710	AT1G56700.2		PTHR23402	KOG4755			pyrrolidone-carboxylate peptidase, putative, expressed
MDC012725.448:862-977	AT5G11260.1	PF00170,PF07716	PTHR13301				bZIP transcription factor domain containing protein
MDC012817.629:817-1307	AT2G38000.1	PF00684		KOG2813			expressed protein
MDC012823.282:222-434	AT3G15690.2	PF00364	PTHR18866:SF1				
MDC012824.191:6532-7133	AT1G30330.2	PF02362,PF06507,PF02309					auxin response factor, putative, expressed
MDC012868.151:2447-3068	AT1G79200.1		PTHR18460				splicing factor, arginine/serine-rich 12, putative, expressed
MDC012916.334:10310-10713	AT3G21700.2	PF08477	PTHR11708	KOG1673			ras-related protein, putative, expressed
MDC012916.334:12813-13262	AT3G21700.1	PF08477	PTHR11708	KOG1673			ras-related protein, putative, expressed
MDC012916.334:13398-13687	AT1G05205.1						expressed protein
MDC012939.284:5004-5485	AT3G27090.1	PF10539	PTHR23230				N-rich protein, putative, expressed
MDC013003.189:32585-33033	AT3G49590.3	PF10033		KOG4573			expressed protein
MDC013090.275:11344-11505	AT3G57450.1						conserved hypothetical protein
MDC013096.127:33850-34454	AT1G15080.1	PF01569	PTHR10165	KOG3030			lipid phosphatase protein, putative, expressed
MDC013173.321:5324-5406	AT5G59840.1	PF00071,PF08477	PTHR11708	KOG0078		K07901	ras-related protein, putative, expressed
MDC013184.220:3935-4234	AT5G47030.1	PF02823	PTHR13822	KOG1758	3.6.3.14	K02134	ATP synthase delta chain, mitochondrial precursor, putative, expressed
MDC013211.128:15219-15773	AT5G12340.1						expressed protein
MDC001250.283:31328-31743	AT3G06740.1	PF00320	PTHR22949:SF3				expressed protein
MDC013254.184:26548-26887	AT5G28750.1	PF02416				K03116	mttA/Hcf106 family protein, putative, expressed
MDC013272.255:2408-2881	AT1G24440.1	PF00097		KOG1039			zinc finger, RING-type, putative, expressed
MDC013292.269:3429-3656	AT3G52930.1	PF00274	PTHR11627	KOG1557	4.1.2.13	K01623	fructose-bisphospate aldolase isozyme, putative, expressed
MDC001262.252:30916-31314	AT4G13780.1	PF09334,PF01588	PTHR11946	KOG1247	6.1.1.10	K01874	methionyl-tRNA synthetase, putative, expressed
MDC013317.553:7451-7667	AT2G36630.1	PF01925	PTHR14255				membrane protein, putative, expressed
MDC013317.563:24791-24933	AT5G56670.1	PF04758	PTHR12650	KOG0009		K02983	expressed protein
MDC013338.153:2636-3071	AT1G18720.1	PF06127		KOG3292			YGL010w, putative, expressed
MDC013392.463:8-454	AT4G31130.1	PF06749					expressed protein
MDC001273.352:1230-1510	AT1G02680.1	PF02269	PTHR11380	KOG3901		K03127	transcription initiation factor IID, 18kD subunit family protein, expressed
MDC013426.256:1484-1797	AT1G33055.1						expressed protein
MDC013531.194:10446-10802	AT3G54820.1	PF00230	PTHR19139	KOG0223		K09872	aquaporin protein, putative, expressed
MDC013531.194:10944-11090	AT2G45960.3	PF00230	PTHR19139	KOG0223		K09872	aquaporin protein, putative, expressed
MDC013531.194:14302-14625	AT3G53420.2	PF00230	PTHR19139	KOG0223		K09872	aquaporin protein, putative, expressed
MDC013567.369:4887-5108	AT5G10700.1		PTHR11717	KOG3305			tyrosine phosphatase, putative, expressed
MDC013616.148:3703-3832	AT5G19140.2	PF12481,PF12504	PTHR11772				stem-specific protein TSJT1, putative, expressed
MDC013616.148:3970-4115	AT5G19140.1	PF12481,PF12504	PTHR11772				stem-specific protein TSJT1, putative, expressed
MDC013616.148:4213-4461	AT5G19140.2	PF12481,PF12504	PTHR11772				stem-specific protein TSJT1, putative, expressed
MDC013737.149:3896-4181	AT4G19645.2	PF03798	PTHR13439	KOG4561			transmembrane protein 56, putative, expressed
MDC001279.248:5742-6162	AT5G16010.1	PF02544	PTHR10556	KOG1638			3-oxo-5-alpha-steroid 4-dehydrogenase, putative, expressed

MDC013772.153:30323-30615	AT3G51100.3						expressed protein
MDC013796.456:5519-6034	AT3G22930.1	PF00036	PTHR23050	KOG0027		K13448	OsCam2 - Calmodulin, expressed
MDC013875.240:1656-1897	AT5G58240.1	PF01230	PTHR23089	KOG3379	3.6.1.29	K01522	histidine triad family protein, putative
MDC013883.433:41972-42740	AT2G27730.1						expressed protein
MDC013917.369:27861-28177	AT5G59970.1	PF00125	PTHR10484	KOG3467		K11254	Core histone H2A/H2B/H3/H4 domain containing protein, putative, expressed
MDC013978.597:11403-11729	AT5G03810.1	PF00657	PTHR22835:SF27				GDSL-like lipase/acylhydrolase, putative, expressed
MDC013998.456:5517-5937	AT3G03000.1	PF00036	PTHR10891	KOG0027		K13448	OsCML11 - Calmodulin-related calcium sensor protein, expressed
MDC014084.122:12618-13135	AT3G04630.3	PF06886					seed specific protein Bn15D14A, putative, expressed
MDC014084.127:14385-14849	AT3G04630.3	PF06886					seed specific protein Bn15D14A, putative, expressed
MDC014088.189:7845-8126	AT3G21770.1	PF00141			1.11.1.7	K00430	peroxidase precursor, putative, expressed
MDC014115.478:5475-5892	AT3G21000.1		PTHR11439				
MDC000185.278:3219-3514	AT3G62880.2	PF02466					
MDC001325.99:2744-3063	AT1G11200.1	PF03619	PTHR23423	KOG2641			domain of unknown function domain containing protein, expressed
MDC014140.253:47-409	AT3G47640.3	PF00010					helix-loop-helix DNA-binding domain containing protein, expressed
MDC014231.203:16823-17997	AT2G36460.1	PF00274	PTHR11627	KOG1557	4.1.2.13	K01623	fructose-bisphospate aldolase isozyme, putative, expressed
MDC015077.305:3146-3873	AT1G33730.1	PF00067	PTHR19383	KOG0156			cytochrome P450, putative, expressed
MDC015153.220:20005-20108	AT5G54580.1	PF00076	PTHR15241	KOG0114			RNA recognition motif containing protein, putative, expressed
MDC015183.122:8990-9049	AT5G47200.1	PF00071,PF08477	PTHR11708	KOG0084		K07976	ras-related protein, putative, expressed
MDC015226.470:54-427	AT5G36880.2	PF11930,PF00501	PTHR11968	KOG1175	6.2.1.1	K01895	AMP-binding enzyme, putative, expressed
MDC015226.508:6534-6780	AT2G18460.1	PF04367					protein of unknown function DUF502 domain containing protein, expressed
MDC015229.223:18448-18603	AT1G08830.2	PF00080	PTHR10003	KOG0441	1.15.1.1	K04565	copper/zinc superoxide dismutase, putative, expressed
MDC015291.262:409-673	AT4G18040.1	PF01652	PTHR11960	KOG1670		K03259	eukaryotic translation initiation factor, putative, expressed
MDC015300.124:5141-5526	AT4G00026.1	PF08294	PTHR13032	KOG4836			AGAP008572-PA, putative, expressed
MDC015306.223:5385-5889	AT1G25230.1	PF00149	PTHR10161	KOG2679			Ser/Thr protein phosphatase family protein, putative, expressed
MDC015317.38:1691-2088	AT2G19450.1	PF03062	PTHR10408	KOG0380	2.3.1.20,2.3.1	K11155	O-acyltransferase, putative, expressed
MDC015354.268:3980-4636	AT2G45260.1	PF04859					GIL1, putative, expressed
MDC015354.268:26756-27169	AT2G45240.1	PF01753,PF00557	PTHR10804	KOG2738	3.4.11.18	K01265	peptidase, M24 family protein, putative, expressed
MDC015354.268:30817-30986	AT2G45240.1	PF01753,PF00557	PTHR10804	KOG2738	3.4.11.18	K01265	peptidase, M24 family protein, putative, expressed
MDC015361.139:5122-6154	AT1G53910.3	PF00847				K09286	AP2 domain containing protein, expressed
MDC015361.232:11883-12412	AT4G17050.1	PF07883					cupin 2, conserved barrel domain protein, putative, expressed
MDC015377.320:8056-8386	AT1G16250.1	PF00646,PF07646,PF01344	PTHR23230				OsFBK22 - F-box domain and kelch repeat containing protein, expressed
MDC015388.349:245-728	AT2G33590.1	PF01370	PTHR10366	KOG1502			reductase, putative, expressed
MDC001370.310:10351-10720	ATMG00300.1		PTHR11439				
MDC015405.307:5999-6733	AT2G27730.1						expressed protein
MDC015405.307:11611-12008	AT2G27450.2	PF00795	PTHR23088	KOG0806			N-carbamoylputrescine amidase, putative, expressed
MDC015460.735:786-1170	AT3G17020.1	PF00582					universal stress protein domain containing protein, putative, expressed
MDC015489.206:24262-35335	AT4G12080.1	PF03479					AT hook motif domain containing protein, expressed
MDC015491.125:1309-1363	AT4G31300.3	PF00227	PTHR11599	KOG0174	3.4.25.1	K02738	peptidase, T1 family, putative, expressed
MDC015509.61:2649-2790	AT5G63160.1	PF00651,PF02135	PTHR23230	KOG1778			BTBZ2 - Bric-a-Brac, Tramtrack, and Broad Complex BTB domain with TAZ zinc finger and Calmodulin-binding domains, expressed
MDC015520.222:32010-33690	AT5G10770.1		PTHR13683	KOG1339			aspartic proteinase nepenthesin-2 precursor, putative, expressed
MDC015556.295:6786-7196	AT4G00030.1	PF04755					PAP fibrillin family domain containing protein, expressed
MDC015585.34:19739-20189	AT3G54280.2	PF02985,PF12054,PF00176,PF00271	PTHR10799	KOG0392			SNF2 family N-terminal domain containing protein, expressed
MDC015655.486:8519-9001	AT5G62200.1	PF06232					embryo-specific 3, putative, expressed
MDC015715.117:4672-5113	AT4G16580.1		PTHR12320	KOG1379			stage II sporulation protein E, putative, expressed
MDC015785.318:410-690	AT3G16080.1	PF01907	PTHR10768	KOG3475		K02922	ribosomal protein L37, putative, expressed
MDC015861.221:33780-34101	AT2G16430.2	PF00149	PTHR22953	KOG1378			purple acid phosphatase precursor, putative, expressed
MDC015884.83:20968-21098	AT1G50370.1	PF00149	PTHR11668	KOG0373	3.1.3.16	K01090	Ser/Thr protein phosphatase family protein, putative, expressed
MDC015884.83:23952-24093	AT3G19980.1	PF00149	PTHR11668	KOG0373	3.1.3.16	K01090	Ser/Thr protein phosphatase family protein, putative, expressed
MDC015884.83:25428-25873	AT3G19980.1	PF00149	PTHR11668	KOG0373	3.1.3.16	K01090	Ser/Thr protein phosphatase family protein, putative, expressed
MDC015885.118:3197-3618	AT1G15520.1	PF00005,PF01061,PF08370	PTHR19241	KOG0065			pleiotropic drug resistance protein, putative, expressed
MDC015885.118:3742-3985	AT1G15520.1	PF00005,PF01061,PF08370	PTHR19241	KOG0065			pleiotropic drug resistance protein, putative, expressed
MDC001441.272:2572-3023	AT3G04300.1	PF05899					enzyme of the cupin superfamily protein, putative, expressed
MDC015915.252:7548-9246	AT5G02500.1	PF00012	PTHR19375	KOG0100		K03283	DnaK family protein, putative, expressed
MDC016026.76:14360-14736	AT1G30580.1	PF01926,PF06071	PTHR23305	KOG1491		K06942	GTP-binding protein, putative, expressed
MDC016027.22:3681-3837	AT4G21105.1	PF02238					COX VIIa, putative, expressed
MDC016044.175:22582-23026	ATMG00310.1		PTHR19446				conserved hypothetical protein
MDC001441.272:13137-13500	AT5G43920.1	PF00400	PTHR22838	KOG0293			WD repeat-containing protein, putative, expressed
MDC016050.65:24-224	AT3G51000.1	PF12146,PF00561	PTHR10992	KOG4178			hydrolase, alpha/beta fold family domain containing protein, expressed
MDC016095.141:7510-7595	AT5G59840.1	PF00071,PF08477	PTHR11708	KOG0078		K07901	ras-related protein, putative, expressed
MDC016096.158:472-890	AT4G21490.1	PF07992,PF00070,PF00036	PTHR22915	KOG2495	1.6.99.3	K03885	NADH-ubiquinone oxidoreductase, mitochondrial precursor, putative, expressed
MDC016098.147:4891-5339	AT1G55280.1	PF03899					expressed protein
MDC000193.346:9537-9769	AT3G04400.2	PF00238	PTHR11761	KOG0901		K02894	ribosomal protein, putative, expressed
MDC016152.155:52339-52623	AT5G27760.1	PF04588					hypoxia-responsive family protein, putative, expressed
MDC016186.171:1006-1298	AT3G58970.1		PTHR13890	KOG2662			CorA-like magnesium transporter protein, putative, expressed
MDC016186.171:1596-2066	AT3G51890.1		PTHR10639				expressed protein
MDC001471.424:94139-94333	AT2G29170.1		PTHR19410				oxidoreductase, short chain dehydrogenase/reductase family domain containing protein, expressed
MDC016234.268:19514-19639	AT5G07050.1	PF00892					auxin-induced protein 5NG4, putative, expressed
MDC016234.268:20057-20273	AT5G07050.1	PF00892					auxin-induced protein 5NG4, putative, expressed
MDC016234.268:20680-20980	AT5G07050.1	PF00892					auxin-induced protein 5NG4, putative, expressed
MDC016261.58:2022-2456	AT4G21460.1	PF10213	PTHR13490:SF4				expressed protein
MDC016265.275:3809-5168	AT4G21990.1	PF01507,PF00085	PTHR18929	KOG0189	1.8.4.9	K05907	
MDC016279.371:5166-5698	AT4G16360.3	PF04739	PTHR10343	KOG1616			SNF1-related protein kinase regulatory subunit beta-1, putative, expressed
MDC016282.141:417-597	AT4G22220.1	PF01592	PTHR10093	KOG3361			iron-sulfur cluster assembly enzyme ISCU, mitochondrial precursor, putative, expressed
MDC016368.213:31185-31562	AT5G24350.2	PF08314		KOG1797			expressed protein
MDC001530.209:10977-11259	AT3G61040.1	PF00067	PTHR19383	KOG0156			cytochrome P450, putative, expressed
MDC016613.108:4593-5046	AT2G33590.1	PF01370	PTHR10366	KOG1502			reductase, putative, expressed
MDC016680.236:5904-6226	AT4G38790.1	PF00810	PTHR10585	KOG3106			ER lumen protein retaining receptor, putative, expressed
MDC001542.287:23512-23825	AT3G30340.1	PF00892					auxin-induced protein 5NG4, putative, expressed
MDC016719.244:7540-8369	AT5G41330.1	PF02214	PTHR11145	KOG2714			B4-BTB4 - Bric-a-Brac, Tramtrack, Broad Complex BTB domain with B4 subfamily conserved sequence, expressed
MDC016733.266:3079-3967	AT5G56670.1	PF04758	PTHR12650	KOG0009		K02983	expressed protein

MDC016767.549:526-899	AT1G21630.2	PF00036	PTHR11216	KOG0998			EF hand family protein, putative, expressed
MDC016767.586:2807-3215	AT1G21630.1	PF00036	PTHR11216	KOG0998			EF hand family protein, putative, expressed
MDC016797.262:982-1299	AT2G24860.1						tsi1-interacting protein TSIP1, putative, expressed
MDC001544.220:3084-3414	AT3G50440.1	PF00561	PTHR10992	KOG1454			OsPOP4 - Putative Prolyl Oligopeptidase homologue, expressed
MDC016994.91:1058-1430	AT5G58740.1	PF04969	PTHR12356	KOG2265			CS domain containing protein, putative, expressed
MDC001568.219:2517-2945	AT1G42960.1						expressed protein
MDC017149.109:416-866	AT3G17020.1	PF00582					universal stress protein domain containing protein, putative, expressed
MDC017149.109:1733-2403	AT3G36659.1	PF04043	PTHR22931:SF5				IWS1 homolog A, putative, expressed
MDC017203.93:49693-51044	AT2G32070.1	PF04857	PTHR10797	KOG0304			CAF1 family ribonuclease containing protein, putative, expressed
MDC017207.68:1532-2126	AT2G01650.1	PF00096,PF09409,PF00789	PTHR23153	KOG2699		K14011	UBX domain containing protein, expressed
MDC017303.260:762-1075	AT1G11880.1	PF04188	PTHR12468	KOG2647	2.4.1.-	K07542	GPI mannosyltransferase 2, putative, expressed
MDC017303.263:3880-4185	AT1G11880.1	PF04188	PTHR12468	KOG2647	2.4.1.-	K07542	GPI mannosyltransferase 2, putative, expressed
MDC017342.179:6762-7645	AT1G04170.1	PF00009,PF03144,PF09173	PTHR23115	KOG0466		K03242	eukaryotic translation initiation factor 2 subunit gamma, putative, expressed
MDC001577.2956:23137-23237	AT3G52930.1	PF00274	PTHR11627	KOG1557	4.1.2.13	K01623	fructose-bisphospate aldolase isozyme, putative, expressed
MDC017415.336:1875-2679	AT3G13275.1						
MDC017440.359:16037-16253	AT4G29230.1	PF02365					no apical meristem protein, putative, expressed
MDC001577.2956:23551-23754	AT5G03690.2	PF00274	PTHR11627	KOG1557	4.1.2.13	K01623	fructose-bisphospate aldolase isozyme, putative, expressed
MDC017491.272:6014-6252	AT2G37660.1		PTHR14194	KOG1203			NAD dependent epimerase/dehydratase family protein, putative, expressed
MDC017491.272:7509-7862	AT2G37660.1		PTHR14194	KOG1203			NAD dependent epimerase/dehydratase family protein, putative, expressed
MDC017518.269:8615-8848	AT1G05190.1	PF00347	PTHR11655	KOG3254			ribosomal protein L6, putative, expressed
MDC017518.269:9967-10438	AT1G05190.1	PF00347	PTHR11655	KOG3254			ribosomal protein L6, putative, expressed
MDC017540.252:42046-42444	AT4G20840.1	PF01565,PF08031	PTHR11748				reticuline oxidase-like protein precursor, putative, expressed
MDC017540.252:45858-46351	AT5G44410.1	PF01565,PF08031					reticuline oxidase-like protein precursor, putative, expressed
MDC017548.358:6850-7605	AT2G09990.1	PF00380	PTHR21569	KOG1753		K02960	ribosomal protein, putative, expressed
MDC017550.46:15204-15624	AT5G59970.1	PF00125	PTHR10484	KOG3467		K11254	Core histone H2A/H2B/H3/H4 domain containing protein, putative, expressed
MDC000205.451:1016-1345	AT5G43600.1	PF01546,PF07687	PTHR11014				peptidase, putative, expressed
MDC001577.2956:23830-23966	AT3G52930.1	PF00274	PTHR11627	KOG1557	4.1.2.13	K01623	fructose-bisphospate aldolase isozyme, putative, expressed
MDC017562.415:3865-4030	AT5G16400.1	PF00085	PTHR10438	KOG0907			thioredoxin, putative, expressed
MDC017562.415:5319-5607	AT3G02730.1	PF00085	PTHR10438	KOG0907			thioredoxin, putative, expressed
MDC017580.217:171-361	AT5G15780.1	PF01190					POEI17 - Pollen Ole e I allergen and extensin family protein precursor, expressed
MDC017593.111:3812-4134	AT2G44650.1	PF00166	PTHR10772	KOG1641			chaperonin, putative, expressed
MDC017611.70:9398-9922	AT5G16790.1	PF05615	PTHR14854	KOG3215		K13176	THO complex subunit 7, putative, expressed
MDC017634.105:22460-22837	AT1G15000.1	PF00450	PTHR11802	KOG1282	3.4.16.-	K09645	OsSCP31 - Putative Serine Carboxypeptidase homologue, expressed
MDC017693.122:6214-6551	AT5G44380.1	PF01565,PF08031	PTHR11748				berberine and berberine like domain containing protein, expressed
MDC017695.289:3392-3935	AT5G60580.4	PF00097	PTHR23012	KOG1609			zinc finger, C3HC4 type, domain containing protein, expressed
MDC017782.228:7791-8049	AT2G28710.1	PF12171,PF00096	PTHR11389				ZOS1-14 - C2H2 zinc finger protein, expressed
MDC017817.273:3318-3686	AT5G07900.1	PF02536		KOG1267			mTERF domain containing protein, expressed
MDC017829.667:3457-3899	AT3G10250.2	PF09713					plant-specific domain TIGR01589 family protein, putative, expressed
MDC001605.331:9735-10084	AT5G10770.1		PTHR13683	KOG1339			aspartic proteinase nepenthesin-2 precursor, putative, expressed
MDC017981.251:962-1158	AT3G48850.1	PF00153	PTHR11896	KOG0767			phosphate carrier protein, mitochondrial precursor, putative, expressed
MDC001627.237:2537-2717	AT1G07440.2	PF00106	PTHR19410	KOG0725			oxidoreductase, short chain dehydrogenase/reductase family protein, putative, expressed
MDC018008.230:9938-10349	AT2G37840.2		PTHR22982		2.7.11.1	K08269	CAMK_CAMK_like_ULKh_APGy.2 - CAMK includes calcium/calmodulin depedent protein kinases, expressed
MDC018009.138:415-646	AT5G37710.1	PF03893,PF01764	PTHR21493	KOG2088			calmodulin-binding heat-shock protein, putative, expressed
MDC018060.144:425-941	AT2G25700.1	PF03931,PF01466	PTHR11165	KOG1724		K03094	SKP1-like protein 1B, putative, expressed
MDC001627.237:2998-3224	AT2G29350.3	PF00106	PTHR19410	KOG0725			oxidoreductase, short chain dehydrogenase/reductase family protein, putative, expressed
MDC018073.537:1656-1915	AT5G65550.1		PTHR11926	KOG1192			glucosyltransferase, putative, expressed
MDC018073.590:292-852	AT5G65550.1		PTHR11926	KOG1192			glucosyltransferase, putative, expressed
MDC018099.109:12995-13315	AT3G17820.1	PF03951,PF00120	PTHR20852	KOG0683	6.3.1.2	K01915	glutamine synthetase, catalytic domain containing protein, expressed
MDC018139.149:19551-19623	AT4G34700.1	PF05347	PTHR12868	KOG3466	1.6.5.3,1.6.99	K03965	LYR motif containing protein, putative, expressed
MDC018160.157:3495-3833	AT2G16850.1	PF00230	PTHR19139	KOG0223		K09872	aquaporin protein, putative, expressed
MDC018160.157:3922-4062	AT5G60660.1	PF00230	PTHR19139	KOG0223		K09872	aquaporin protein, putative, expressed
MDC001627.237:4701-5152	AT2G29330.1	PF00106	PTHR19410	KOG0725	1.1.1.206	K08081	oxidoreductase, short chain dehydrogenase/reductase family domain containing protein, expressed
MDC018160.157:4161-4370	AT4G35100.2	PF00230	PTHR19139	KOG0223		K09872	aquaporin protein, putative, expressed
MDC018160.157:4570-4823	AT2G16850.1	PF00230	PTHR19139	KOG0223		K09872	aquaporin protein, putative, expressed
MDC018334.222:6621-6929	AT2G46070.2	PF00069,PF07714	PTHR11295	KOG0660	2.7.11.24	K04371	CGMC_MAPKCMGC_2_ERK.13 - CGMC includes CDA, MAPK, GSK3, and CLKC kinases, expressed
MDC018355.124:5469-5749	AT2G32950.1	PF00097,PF00400	PTHR22847	KOG0265	6.3.2.19	K10143	COP1, putative, expressed
MDC018359.70:1069-1579	AT2G02230.1	PF00646					OsFBX70 - F-box domain containing protein, expressed
MDC018409.250:3724-4557	AT2G09990.1	PF00380	PTHR21569	KOG1753		K02960	ribosomal protein, putative, expressed
MDC018412.103:26641-27151	AT3G01360.2	PF04819					viral-response family protein, expressed
MDC018491.206:16889-17230	AT5G11110.1	PF08550,PF00534	PTHR12526	KOG0853			sucrose-phosphate synthase, putative, expressed
MDC018507.307:20436-20547	AT1G23260.1	PF00179	PTHR11621	KOG0896		K10704	ubiquitin-conjugating enzyme, putative, expressed
MDC018507.307:20621-20737	AT2G36060.3		PTHR11621	KOG0896		K10704	ubiquitin-conjugating enzyme, putative, expressed
MDC018507.307:20888-21033	AT1G23260.1	PF00179	PTHR11621	KOG0896		K10704	ubiquitin-conjugating enzyme, putative, expressed
MDC018556.272:150-522	AT5G11760.1	PF08576					expressed protein
MDC001661.298:30577-31330	AT1G01980.1	PF01565,PF08031		KOG1231			reticuline oxidase-like protein precursor, putative, expressed
MDC018580.424:39-984	AT3G58030.4	PF00097	PTHR12313	KOG0823			zinc finger, C3HC4 type domain containing protein, expressed
MDC018648.267:14763-15015	AT3G44260.1	PF04857	PTHR10797	KOG0304			CAF1 family ribonuclease containing protein, putative, expressed
MDC018648.267:15175-15555	AT3G44260.1	PF04857	PTHR10797	KOG0304			CAF1 family ribonuclease containing protein, putative, expressed
MDC018667.247:1783-1896	AT4G23880.1						
MDC001665.416:2559-2944	AT4G38020.1	PF00588	PTHR12029	KOG2506			RNA methyltransferase, TrmH family protein, putative, expressed
MDC018728.83:12896-13234	AT3G05545.1	PF00097	PTHR22766				zinc finger, C3HC4 type domain containing protein, expressed
MDC018728.83:23735-24128	AT3G05530.1	PF07728,PF00004	PTHR23073	KOG0652		K03065	26S protease regulatory subunit 6A, putative, expressed
MDC018728.83:24253-24390	AT3G05530.1	PF07728,PF00004	PTHR23073	KOG0652		K03065	26S protease regulatory subunit 6A, putative, expressed
MDC018728.83:24487-24595	AT3G05530.1	PF07728,PF00004	PTHR23073	KOG0652		K03065	26S protease regulatory subunit 6A, putative, expressed
MDC018744.264:10711-11303	AT2G30950.1	PF06480,PF07728,PF00004,PF01434	PTHR23076	KOG0731	3.4.24.-	K03798	OsFtsH2 FtsH protease, homologue of AtFtsH2/8, expressed
MDC018744.264:11400-11646	AT1G06430.1	PF06480,PF07728,PF00004,PF01434	PTHR23076	KOG0731	3.4.24.-	K03798	OsFtsH2 FtsH protease, homologue of AtFtsH2/8, expressed
MDC018820.163:4887-5031	AT1G67530.2	PF04564,PF00514	PTHR22849				armadillo/beta-catenin repeat family protein, putative, expressed
MDC018837.177:26042-26469	AT5G41350.1	PF00097	PTHR22937				zinc finger, C3HC4 type domain containing protein, expressed
MDC018870.194:4803-4978	AT2G33590.1	PF01370	PTHR10366	KOG1502			reductase, putative, expressed
MDC018977.108:28169-28577	AT3G13677.2						expressed protein

MDC018995.224:13429-13760	AT4G30780.1						expressed protein
MDC019082.128:8813-9112	AT4G16500.1	PF00031					cysteine proteinase inhibitor 8 precursor, putative, expressed
MDC019091.216:2033-2693	AT5G21940.1						MTD1, putative, expressed
MDC019091.231:35553-36009	AT3G43860.1	PF00759	PTHR22298		3.2.1.4	K01179	endoglucanase, putative, expressed
MDC019095.63:22484-23000	AT5G09590.1	PF00012	PTHR19375	KOG0102		K03283	DnaK family protein, putative, expressed
MDC001766.131:11010-11410	AT4G32760.2	PF00790,PF03127	PTHR13856	KOG1087			VHS and GAT domain containing protein, expressed
MDC019117.269:5681-6073	AT4G13520.1						
MDC019147.46:9270-9569	AT1G54250.1	PF03870	PTHR10917	KOG3400		K03016	DNA-directed RNA polymerases I, II, and III subunit RPABC3, putative, expressed
MDC019238.44:5191-5727	AT5G18200.1		PTHR11943	KOG2958	2.7.7.12	K00965	galactose-1-phosphate uridyl transferase, putative, expressed
MDC019339.84:8665-9426	AT2G22420.1	PF00141			1.11.1.7	K00430	peroxidase precursor, putative, expressed
MDC019360.344:23048-23730	AT3G59080.2		PTHR13683	KOG1339			aspartic proteinase nepenthesin precursor, putative, expressed
MDC019368.126:5277-5781	AT1G67265.1	PF08137					expressed protein
MDC019383.46:34094-34336	AT5G48070.1	PF00722,PF06955			2.4.1.207	K08235	glycosyl hydrolases family 16, putative, expressed
MDC019400.208:6391-6498	AT5G47930.1	PF01667	PTHR11594	KOG1779		K02978	40S ribosomal protein S27, putative, expressed
MDC019454.54:10125-10559	AT5G14420.2	PF07002,PF00097	PTHR10857	KOG1327			copine, putative, expressed
MDC019461.80:27261-27542	AT5G52390.1	PF06521					
MDC019461.80:27755-28178	AT5G52390.1	PF06521					
MDC019484.38:4830-5300	AT2G39110.1	PF00069,PF07714	PTHR23258	KOG1187			protein kinase APK1A, chloroplast precursor, putative, expressed
MDC019522.115:11000-11388	AT5G11600.1						expressed protein
MDC001859.240:11026-11424	AT3G55070.1		PTHR12170	KOG0396			macrophage erythroblast attacher, putative, expressed
MDC019537.450:7236-7659	AT3G09700.1		PTHR12763	KOG0723			heat shock protein DnaJ, putative, expressed
MDC019598.191:5158-5543	ATMG00300.1		PTHR11439				
MDC019710.194:10352-10604	AT1G43650.2	PF00892					integral membrane protein DUF6 containing protein, expressed
MDC019710.194:11838-12171	AT1G43650.1	PF00892					integral membrane protein DUF6 containing protein, expressed
MDC019710.194:13383-13777	AT1G43650.1	PF00892					integral membrane protein DUF6 containing protein, expressed
MDC019720.430:2208-2873	AT5G24860.1						flowering promoting factor-like 1, putative, expressed
MDC001883.280:6135-6504	AT5G16790.1	PF05615	PTHR14854	KOG3215		K13176	THO complex subunit 7, putative, expressed
MDC019839.138:30491-30850	AT4G36130.1	PF00181,PF03947	PTHR13691	KOG2309		K02938	60S ribosomal protein L8, putative, expressed
MDC019846.92:3774-4356	AT5G44380.1	PF01565,PF08031	PTHR11748				berberine and berberine like domain containing protein, expressed
MDC019854.101:11089-11317	AT1G55740.1	PF05691					uncharacterized glycosyltransferase, putative, expressed
MDC019950.115:0-456	AT2G21110.1	PF03018	PTHR21495				dirigent, putative, expressed
MDC019959.138:16420-17121	AT2G44470.3	PF00232	PTHR10353	KOG0626	3.2.1.147	K01237	Os6bglu24 - beta-glucosidase homologue, similar to G. max isohydroxyurate hydrolase, expressed
MDC020065.141:605-1042	AT2G39420.1	PF12146,PF00561	PTHR11614	KOG1455			lipase, putative, expressed
MDC020127.174:13627-13974	AT2G18280.2	PF01167	PTHR16517	KOG2502			OsFBT4 - F-box and tubby domain containing protein, expressed
MDC020196.147:693-1117	AT4G29380.1	PF00069,PF02985,PF00400	PTHR22971	KOG1240	2.7.11.1	K08333	WD domain and HEAT domain containing protein, putative, expressed
MDC020226.287:8976-9718	AT5G46770.1						
MDC020272.72:5-623	AT3G25600.1	PF00036	PTHR10891	KOG0027		K13448	OsCML14 - Calmodulin-related calcium sensor protein, expressed
MDC000218.595:8811-8927	AT2G18600.1	PF00179	PTHR11621	KOG0420	6.3.2.19	K10579	ubiquitin-conjugating enzyme, putative, expressed
MDC020310.146:2259-2549	AT3G57770.1		PTHR23258				OsWAK53b - OsWAK receptor-like protein kinase, expressed
MDC020310.146:5216-5468	AT3G57770.1		PTHR23258				OsWAK53b - OsWAK receptor-like protein kinase, expressed
MDC020353.88:17968-18166	AT5G66450.2	PF01569	PTHR11247	KOG3146			phosphatase, putative, expressed
MDC020364.514:4946-5132	AT5G15790.2	PF00097	PTHR22766				zinc finger, C3HC4 type domain containing protein, expressed
MDC020450.123:5627-6146	AT4G03030.1	PF00646,PF01344,PF07646	PTHR23230:SF146				OsFBK6 - F-box domain and kelch repeat containing protein, expressed
MDC020479.248:5861-6227	AT1G43640.1	PF00646,PF01167	PTHR16517	KOG2502			OsFBT6 - F-box and tubby domain containing protein, expressed
MDC020690.260:22415-22574	AT5G64200.2	PF00076	PTHR23147	KOG4207		K12891	RNA recognition motif containing protein, expressed
MDC020733.167:1678-2210	AT1G55160.3						expressed protein
MDC020827.218:21752-22150	AT2G24940.1	PF00173	PTHR10281				cytochrome b5-like Heme/Steroid binding domain containing protein, expressed
MDC020867.67:244-615	AT5G62890.3	PF00860	PTHR11119	KOG1292			nucleobase-ascorbate transporter, putative, expressed
MDC021014.157:18162-18516	AT4G31720.1	PF03540	PTHR21242			K03134	transcription initiation factor TFIID subunit 10, putative, expressed
MDC021147.99:1202-1451	AT2G03500.1	PF00249					MYB family transcription factor, putative, expressed
MDC021331.203:2656-3088	AT5G11650.1	PF12146,PF00561	PTHR11614	KOG1455			hydrolase, alpha/beta fold family domain containing protein, expressed
MDC021393.363:4685-5100	AT4G39730.1	PF01477					wound/stress protein, putative, expressed
MDC021461.112:3144-3560	AT1G79510.2	PF10184		KOG4457			expressed protein
MDC021463.473:17752-18244	AT4G10840.1	PF07719,PF00515,PF07721	PTHR19959	KOG1840			tetratricopeptide repeat domain containing protein, expressed
MDC001963.417:4488-4646	AT4G27250.2	PF01370	PTHR10366	KOG1502			dihydroflavonol-4-reductase, putative, expressed
MDC021607.243:3134-3692	AT5G10520.1	PF00069,PF07714	PTHR23258	KOG1187			protein kinase domain containing protein, expressed
MDC021609.524:3718-3898	AT5G34850.1	PF00149	PTHR22953	KOG1378			Ser/Thr protein phosphatase family protein, putative, expressed
MDC021614.36:14793-15075	AT1G34760.2	PF00244	PTHR18860	KOG0841			14-3-3 protein, putative, expressed
MDC001963.417:5141-5310	AT4G27250.2	PF01370	PTHR10366	KOG1502			dihydroflavonol-4-reductase, putative, expressed
MDC021658.157:7798-8265	AT1G10950.1	PF02990,PF09680	PTHR10766	KOG1277			transmembrane 9 superfamily member, putative, expressed
MDC021663.22:7322-7560	AT5G59250.1	PF00083,PF07690	PTHR11600	KOG0254			transporter family protein, putative, expressed
MDC021673.67:2187-2416	AT5G15780.1	PF01190					POEI17 - Pollen Ole e I allergen and extensin family protein precursor, expressed
MDC021679.163:8234-8578	AT2G46020.1	PF08880,PF00176,PF00271,PF00439	PTHR10799	KOG0386			SNF2 family N-terminal domain containing protein, expressed
MDC021726.381:2654-2885	AT1G33810.1						expressed protein
MDC021888.183:8090-8386	AT5G60660.1	PF00230	PTHR19139	KOG0223		K09872	aquaporin protein, putative, expressed
MDC021946.181:5025-5983	AT3G44260.1	PF04857	PTHR10797	KOG0304			CAF1 family ribonuclease containing protein, putative, expressed
MDC021991.115:13313-13634	AT4G34265.2						
MDC022012.425:3903-4199	AT3G03080.1	PF00107	PTHR11695	KOG1196	1.3.1.74	K08070	NADP-dependent oxidoreductase, putative, expressed
MDC002052.246:116-455	AT3G55360.1	PF02544	PTHR10556	KOG1639	1.3.1.-	K10258	3-oxo-5-alpha-steroid 4-dehydrogenase, putative, expressed
MDC022074.29:9529-10735	AT2G37220.1	PF00076	PTHR10432	KOG0131			RNA recognition motif containing protein, putative, expressed
MDC022074.29:11143-11246	AT2G37220.1	PF00076	PTHR10432	KOG0131			RNA recognition motif containing protein, putative, expressed
MDC022078.140:14217-14720	AT5G64816.2						THION26 - Plant thionin family protein precursor, expressed
MDC022085.43:356-778	AT4G31300.3	PF00227	PTHR11599	KOG0174	3.4.25.1	K02738	peptidase, T1 family, putative, expressed
MDC022085.65:6537-7053	AT4G31300.3	PF00227	PTHR11599	KOG0174	3.4.25.1	K02738	peptidase, T1 family, putative, expressed
MDC022086.272:6317-6499	AT1G07440.2	PF00106	PTHR19410	KOG0725			oxidoreductase, short chain dehydrogenase/reductase family protein, putative, expressed
MDC022086.272:6776-7049	AT1G07440.2	PF00106	PTHR19410	KOG0725			oxidoreductase, short chain dehydrogenase/reductase family protein, putative, expressed
MDC022086.272:7788-8194	AT2G29330.1	PF00106	PTHR19410	KOG0725	1.1.1.206	K08081	oxidoreductase, short chain dehydrogenase/reductase family domain containing protein, expressed
MDC022119.74:4893-5155	AT1G65270.1			KOG4827			expressed protein
MDC022251.179:6243-7113	AT3G14310.1	PF04043,PF01095					pectinesterase, putative, expressed
MDC022362.462:10497-10811	AT1G03210.1	PF02567	PTHR13774	KOG3033		K06998	phenazine biosynthesis protein, putative, expressed

MDC022362.462:11464-11752	AT4G02860.1	PF02567	PTHR13774	KOG3033			phenazine biosynthesis protein, putative, expressed
MDC022376.75:3053-3379	AT3G03940.1	PF00069	PTHR11909	KOG1164			CK1_CaseinKinase_1a.3 - CK1 includes the casein kinase 1 kinases, expressed
MDC022484.73:18974-19931	AT3G13920.4	PF00270,PF00271	PTHR10967	KOG0328			DEAD-box ATP-dependent RNA helicase, putative, expressed
MDC022484.73:20443-20879	AT1G54270.2	PF00270,PF00271	PTHR10967	KOG0327		K03257	DEAD-box ATP-dependent RNA helicase, putative, expressed
MDC002113.246:13095-13300	AT2G25910.2	PF01612,PF00013	PTHR12124				3-5 exonuclease/ nucleic acid binding protein, putative, expressed
MDC022597.70:2400-2698	AT1G08410.1	PF01926	PTHR11089	KOG1424	3.6.1.-	K14539	GTPase of unknown function domain containing protein, putative, expressed
MDC022628.340:10959-11393	AT2G30950.1	PF06480,PF07728,PF00004,PF01434	PTHR23076	KOG0731	3.4.24.-	K03798	OsFtsH2 FtsH protease, homologue of AtFtsH2/8, expressed
MDC022628.340:12342-12976	AT2G30950.1	PF06480,PF07728,PF00004,PF01434	PTHR23076	KOG0731	3.4.24.-	K03798	OsFtsH2 FtsH protease, homologue of AtFtsH2/8, expressed
MDC022628.341:355-501	AT1G06430.1	PF06480,PF07728,PF00004,PF01434	PTHR23076	KOG0731	3.4.24.-	K03798	OsFtsH2 FtsH protease, homologue of AtFtsH2/8, expressed
MDC022637.126:5119-5368	AT1G19640.1	PF03492			2.1.1.141	K08241	SAM dependent carboxyl methyltransferase, putative, expressed
MDC022637.126:5454-5802	AT1G19640.1	PF03492			2.1.1.141	K08241	SAM dependent carboxyl methyltransferase, putative, expressed
MDC022637.126:6384-6996	AT1G19640.1	PF03492			2.1.1.141	K08241	SAM dependent carboxyl methyltransferase, putative, expressed
MDC022638.160:2961-3442	AT1G13245.1	PF08137					expressed protein
MDC022642.185:459-976	AT1G45180.1	PF00097	PTHR14155				zinc finger, C3HC4 type domain containing protein, expressed
MDC022756.223:2387-2824	AT2G40935.1	PF04749					uncharacterized Cys-rich domain containing protein, putative, expressed
MDC022778.400:862-1178	AT1G22510.2	PF06803	PTHR22894	KOG4604			zinc finger, C3HC4 type domain containing protein, expressed
MDC022787.507:14430-14765	AT4G23160.1	PF07727,PF01657,PF00069,PF07714,PF11883	PTHR11439	KOG1187			TKL_IRAK_DUF26-lc.20 - DUF26 kinases have homology to DUF26 containing loci, expressed
MDC022791.14:17058-17637	AT5G16970.1	PF00107	PTHR11695	KOG1196	1.3.1.74	K08070	NADP-dependent oxidoreductase, putative, expressed
MDC022803.125:8318-8996	AT5G28540.1	PF00012	PTHR19375	KOG0100		K09490	DnaK family protein, putative, expressed
MDC022824.308:8367-8863	AT4G32551.2	PF08513,PF00400	PTHR22847	KOG0266			transcriptional corepressor LEUNIG, putative, expressed
MDC022831.234:2-828	AT4G17690.1	PF00141			1.11.1.7	K00430	peroxidase family protein, expressed
MDC022831.238:287-403	AT4G27960.2	PF00179	PTHR11621	KOG0417			ubiquitin-conjugating enzyme, putative, expressed
MDC022880.59:13085-13467	AT4G30900.2						endonuclease/exonuclease/phosphatase family protein, putative, expressed
MDC022888.364:12533-12667	AT3G51730.1	PF05184,PF03489	PTHR11480	KOG1340			saposin-like type B, region 1 family protein, putative, expressed
MDC022888.364:12994-13189	AT3G51730.1	PF05184,PF03489	PTHR11480	KOG1340			saposin-like type B, region 1 family protein, putative, expressed
MDC022888.364:14577-14856	AT3G51730.1	PF05184,PF03489	PTHR11480	KOG1340			saposin-like type B, region 1 family protein, putative, expressed
MDC023032.62:43936-44459	AT5G53220.3						expressed protein
MDC023531.44:2818-2958	AT3G59540.1	PF01781	PTHR10965	KOG3499		K02923	60S ribosomal protein L38, putative, expressed
MDC023633.27:5099-5516	AT3G11670.2				2.4.1.241	K09480	digalactosyldiacylglycerol synthase, chloroplast precursor, putative, expressed
MDC023670.41:2268-2781	AT2G33590.1	PF01370	PTHR10366	KOG1502			reductase, putative, expressed
MDC026269.25:2726-2851	AT5G64730.1	PF00400	PTHR22842	KOG0316		K13124	WD domain, G-beta repeat domain containing protein, expressed
MDC026617.62:10076-10400	AT5G56190.2	PF00400	PTHR22847				WD-40 repeat family protein, putative, expressed
MDC002159.340:12619-13184	AT1G78290.3	PF00069	PTHR22982	KOG0583	2.7.11.1	K14498	CAMK_CAMK_like.7 - CAMK includes calcium/calmodulin depedent protein kinases, expressed
MDC027871.11:6913-8058	AT2G45260.1	PF04859					GIL1, putative, expressed
MDC032130.10:2771-3287	AT1G08180.1						
MDC036854.6:543-1070	AT2G46495.1	PF00097	PTHR22764				zinc finger, C3HC4 type domain containing protein, expressed
MDC002191.244:5992-6164	AT5G47030.1	PF02823	PTHR13822	KOG1758	3.6.3.14	K02134	ATP synthase delta chain, mitochondrial precursor, putative, expressed
MDC036969.18:8353-8496	AT1G15270.1	PF09072					coiled-coil domain-containing protein 72, putative, expressed
MDC040767.9:10828-11034	AT3G02250.1	PF10250					auxin-independent growth promoter protein, putative, expressed
MDC002203.349:4228-4862	AT1G62290.2	PF00026,PF03489,PF05184	PTHR13683	KOG1339	3.4.23.40	K08245	aspartic proteinase oryzasin-1 precursor, putative, expressed
MDC002205.555:7793-8082	AT1G11910.1	PF00026,PF03489,PF05184	PTHR13683	KOG1339	3.4.23.40	K08245	aspartic proteinase oryzasin-1 precursor, putative, expressed
MDC002205.555:8858-9031	AT1G62290.2	PF00026,PF03489,PF05184	PTHR13683	KOG1339	3.4.23.40	K08245	aspartic proteinase oryzasin-1 precursor, putative, expressed
MDC002205.604:5796-6019	AT3G15351.2						expressed protein
MDC002252.327:4583-5069	AT1G07520.1	PF03514					SCARECROW, putative, expressed
MDC002319.140:10705-11101	AT2G38905.1	PF01679	PTHR21659	KOG1773			OsRCI2-7 - Putative low temperature and salt responsive protein, expressed
MDC000071.210:1438-1573	AT4G16695.3						
MDC002360.331:36076-36218	AT3G17760.2	PF00282	PTHR11999	KOG1383	4.1.1.15	K01580	decarboxylase, putative, expressed
MDC002367.269:48264-48619	AT5G17330.1	PF00282	PTHR11999	KOG1383	4.1.1.15	K01580	glutamate decarboxylase, putative, expressed
MDC002367.269:48993-49472	AT1G65960.2	PF00282	PTHR11999	KOG1383	4.1.1.15	K01580	glutamate decarboxylase, putative, expressed
MDC002367.269:49581-49783	AT4G13195.1						
MDC002385.290:19935-20648	AT4G13230.1		PTHR23241:SF11				
MDC002412.304:11037-11650	AT5G19130.2	PF04114	PTHR13304	KOG3566		K05289	GPI transamidase component family protein, putative, expressed
MDC002431.300:12026-12395	AT3G26510.5	PF00564					PB1 domain containing protein, expressed
MDC002441.272:2839-3539	ATCG01250.1	PF00361	PTHR22773	KOG4668			NADPH-dependent oxidoreductase, putative
MDC002450.221:3501-4098	AT3G17668.1						expressed protein
MDC002453.447:10532-10849	AT3G18570.1	PF01277					oleosin, putative, expressed
MDC000298.380:1328-1593	AT5G56630.1	PF00365	PTHR13697	KOG2440	2.7.1.11	K00850	6-phosphofructokinase, putative, expressed
MDC002536.231:28261-28837	AT5G02520.1	PF09133					expressed protein
MDC002555.573:6522-6894	AT2G38740.1	PF00702	PTHR18901	KOG2914			haloacid dehalogenase-like hydrolase family protein, putative, expressed
MDC002562.442:3585-4052	AT2G38740.1	PF00702	PTHR18901	KOG2914			haloacid dehalogenase-like hydrolase family protein, putative, expressed
MDC002562.442:4163-4526	AT2G38740.1	PF00702	PTHR18901	KOG2914			haloacid dehalogenase-like hydrolase family protein, putative, expressed
MDC002562.442:4820-4968	AT2G38740.1	PF00702	PTHR18901	KOG2914			haloacid dehalogenase-like hydrolase family protein, putative, expressed
MDC002562.442:5044-5170	AT3G12587.1	PF10215					expressed protein
MDC002563.449:3601-3812	AT4G02290.1	PF00759	PTHR22298				endoglucanase, putative, expressed
MDC002579.350:565-1558	AT1G07440.2	PF00106	PTHR19410	KOG0725			oxidoreductase, short chain dehydrogenase/reductase family protein, putative, expressed
MDC002584.153:3776-3993	AT4G10840.1	PF07719,PF00515,PF07721	PTHR19959	KOG1840			tetratricopeptide repeat domain containing protein, expressed
MDC002655.565:10635-11102	AT4G10840.1	PF07719,PF00515,PF07721	PTHR19959	KOG1840			tetratricopeptide repeat domain containing protein, expressed
MDC002655.586:7567-8051	AT5G51700.1	PF04968	PTHR12621			K13458	rar1, putative, expressed
MDC002662.815:1633-1925	AT4G19003.1	PF05871	PTHR13149	KOG4068		K12189	vacuolar protein-sorting-associated protein 25, putative, expressed
MDC002663.516:8880-9798	AT3G12030.1	PF01956	PTHR20917	KOG3312			fb27, putative, expressed
MDC002683.322:3279-3696	AT1G23440.1		PTHR23402	KOG4755			pyrrolidone-carboxylate peptidase, putative, expressed
MDC002758.275:5034-5548	AT1G23440.1		PTHR23402	KOG4755			pyrrolidone-carboxylate peptidase, putative, expressed
MDC002758.275:7054-7287	AT1G52570.1	PF00168,PF00614,PF12357	PTHR18896	KOG1329			phospholipase D, putative, expressed
MDC002787.258:19523-20073	AT4G17690.1	PF00141			1.11.1.7	K00430	peroxidase family protein, expressed
MDC002848.461:9691-10126	AT1G66950.1	PF00005,PF01061,PF08370	PTHR19241	KOG0065			pleiotropic drug resistance protein, putative, expressed
MDC000368.236:1585-1999	AT2G01410.1						expressed protein
MDC002955.279:38802-39101	AT1G19240.1						expressed protein
MDC002986.271:21-529	AT1G02790.1	PF00295			3.2.1.67	K01213	polygalacturonase, putative, expressed
MDC003026.402:4690-4972	AT2G15450.1	PF00295					polygalacturonase, putative, expressed
MDC003026.402:5565-6093	AT5G65550.1		PTHR11926	KOG1192			glucosyltransferase, putative, expressed

MDC003081.405:1372-1864	AT5G65550.1		PTHR11926	KOG1192			glucosyltransferase, putative, expressed
MDC003081.405:1945-2200	AT4G21120.1	PF00324	PTHR11785	KOG1286			amino acid permease family protein, putative
MDC000368.238:33490-34602	AT3G07760.2	PF00536					expressed protein
MDC003198.202:2865-3190	AT5G53490.3	PF00805					thylakoid lumenal protein, putative, expressed
MDC003205.158:29546-29798	AT5G53490.3	PF00805					thylakoid lumenal protein, putative, expressed
MDC003205.158:29880-30320	AT2G46550.2						expressed protein
MDC003205.158:76934-77309	AT1G30450.2	PF00324	PTHR11827	KOG2082			amino acid permease family protein, putative, expressed
MDC000368.238:51135-51386	AT4G35100.2	PF00230	PTHR19139	KOG0223		K09872	aquaporin protein, putative, expressed
MDC003306.225:3479-3782	AT2G16850.1	PF00230	PTHR19139	KOG0223		K09872	aquaporin protein, putative, expressed
MDC003306.225:3868-4016	AT2G16850.1	PF00230	PTHR19139	KOG0223		K09872	aquaporin protein, putative, expressed
MDC003306.225:4116-4489	AT3G02720.1	PF01965	PTHR11019	KOG2764			DJ-1 family protein, putative, expressed
MDC003313.350:8410-8848	AT1G31817.1	PF00411	PTHR11759	KOG0408			ribosomal protein, putative, expressed
MDC003322.305:8692-9506	AT4G21440.1	PF00249	PTHR10641	KOG0048		K09422	MYB family transcription factor, putative, expressed
MDC003396.159:7266-7693	AT3G16340.2	PF01061,PF08370,PF00005	PTHR19241	KOG0065			pleiotropic drug resistance protein, putative, expressed
MDC003415.452:48231-48632	AT5G18790.1	PF00471		KOG3505			50S ribosomal protein L33, putative, expressed
MDC003417.148:4832-5225	AT2G02990.1	PF00445	PTHR11240	KOG1642			ribonuclease T2 family domain containing protein, expressed
MDC003465.666:11182-11467	AT2G47680.1	PF00271,PF00642	PTHR18934	KOG0920			zinc finger helicase family protein, putative, expressed
MDC003476.424:4739-5090	AT2G40370.1	PF07732,PF00394,PF07731	PTHR11709	KOG1263			laccase precursor protein, putative, expressed
MDC003534.362:4332-4810	AT5G48640.1	PF00134	PTHR10026	KOG0794			cyclin, putative, expressed
MDC003623.656:7835-8044	AT4G00585.1						expressed protein
MDC003652.401:5479-5810	AT3G58560.1	PF03372	PTHR12121	KOG0620	3.1.-.-	K12603	endonuclease/exonuclease/phosphatase family domain containing protein, expressed
MDC003661.185:20810-21803	AT5G08100.1	PF01112	PTHR10188	KOG1592	3.4.19.5	K13051	L-asparaginase precursor protein, putative, expressed
MDC003664.475:11513-12117	AT5G08120.1						microtubule-associated protein, putative, expressed
MDC003664.475:17093-17401	AT5G36110.1	PF00067	PTHR19383	KOG0157			cytochrome P450, putative, expressed
MDC003668.599:3948-4504	AT5G36110.1	PF00067	PTHR19383	KOG0157			cytochrome P450, putative, expressed
MDC003668.599:4608-4827	AT4G23420.3	PF00106	PTHR19410	KOG1208			oxidoreductase, short chain dehydrogenase/reductase family domain containing family, expressed
MDC003691.613:12408-12611	AT4G23420.3	PF00106	PTHR19410	KOG1208			oxidoreductase, short chain dehydrogenase/reductase family domain containing family, expressed
MDC003691.613:13105-13267	AT4G23420.3	PF00106	PTHR19410	KOG1208			oxidoreductase, short chain dehydrogenase/reductase family domain containing family, expressed
MDC003691.613:14240-14582	AT2G18110.1	PF00736	PTHR11595	KOG1668		K03232	elongation factor protein, putative, expressed
MDC003707.256:151-617	AT2G02720.1	PF04431,PF00544			4.2.2.2	K01728	pectate lyase precursor, putative, expressed
MDC000416.247:35408-35747	AT1G11910.1	PF00026,PF03489,PF05184	PTHR13683	KOG1339	3.4.23.40	K08245	aspartic proteinase oryzasin-1 precursor, putative, expressed
MDC003743.404:7074-7268	AT1G08230.2	PF01490	PTHR22950	KOG1303			transmembrane amino acid transporter protein, putative, expressed
MDC003758.352:13113-13479	AT3G20600.1						harpin-induced protein 1 domain containing protein, expressed
MDC003785.419:14126-14836	AT2G43780.2						conserved hypothetical protein
MDC003785.419:15852-16189	AT3G20800.1	PF04078	PTHR12262	KOG3036		K12606	expressed protein
MDC003833.254:923-1391	AT1G14420.1	PF04431,PF00544			4.2.2.2	K01728	pectate lyase precursor, putative, expressed
MDC000416.247:35907-36548	AT5G66740.1	PF04788					expressed protein
MDC003876.694:2083-2790	AT5G66740.1	PF04788					expressed protein
MDC003876.694:3305-3512	AT1G13360.1						expressed protein
MDC003878.401:325-1733	AT2G40470.1	PF03195					DUF260 domain containing protein, putative, expressed
MDC003882.313:12016-12674	AT2G42760.1						expressed protein
MDC003889.426:7020-7694	AT1G71010.1	PF01504	PTHR23086	KOG0230			1-phosphatidylinositol-4-phosphate 5-kinase/ zinc ion binding protein, putative, expressed
MDC000421.511:6588-6935	AT2G01940.3	PF12171,PF00096	PTHR11389				ZOS8-07 - C2H2 zinc finger protein, expressed
MDC003936.406:51932-52906	AT1G27530.1	PF08694	PTHR12921	KOG3357		K12165	ufm1-conjugating enzyme 1, putative, expressed
MDC003963.399:2459-2839	AT1G48830.2	PF01251	PTHR11278	KOG3320			40S ribosomal protein S7, putative
MDC004009.559:39608-40030	AT1G48830.2	PF01251	PTHR11278	KOG3320			40S ribosomal protein S7, putative
MDC004009.559:40130-40478	AT1G48830.2	PF01251	PTHR11278	KOG3320			40S ribosomal protein S7, putative
MDC004009.559:40660-40931	AT3G19760.1	PF00270,PF00271	PTHR10967	KOG0328	3.6.4.13	K13025	DEAD-box ATP-dependent RNA helicase, putative, expressed
MDC004010.194:30326-30466	AT2G01970.1	PF02990	PTHR10766	KOG1277			transmembrane 9 superfamily member, putative, expressed
MDC004088.250:961-1337	AT1G07830.1	PF06984,PF00831	PTHR21183	KOG3331			39S ribosomal protein L47, mitochondrial precursor, putative, expressed
MDC004095.243:345-694	AT2G46150.1	PF03168					harpin-induced protein 1 domain containing protein, expressed
MDC004097.230:13303-14099	AT3G54200.1	PF03168					harpin-induced protein 1 domain containing protein, expressed
MDC004097.232:10956-11698	AT2G44360.1						
MDC004168.448:5563-5714	AT2G17990.1						calcium-dependent protein kinase CPK1 adapter protein 2, putative, expressed
MDC004176.297:3407-3797	AT1G07440.2	PF00106	PTHR19410	KOG0725			oxidoreductase, short chain dehydrogenase/reductase family protein, putative, expressed
MDC004279.272:6053-6248	AT1G07440.2	PF00106	PTHR19410	KOG0725			oxidoreductase, short chain dehydrogenase/reductase family protein, putative, expressed
MDC004279.407:7341-7593	AT2G29320.1	PF00106	PTHR19410	KOG0725	1.1.1.206	K08081	oxidoreductase, short chain dehydrogenase/reductase family domain containing protein, expressed
MDC004279.407:8692-9013	AT5G48960.1	PF05761	PTHR12103	KOG2469			5-nucleotidase domain-containing protein, putative, expressed
MDC004302.250:3053-3502	AT3G48140.1	PF06522					B12D protein, putative, expressed
MDC000479.396:652-1113	AT5G18150.1		PTHR12133				expressed protein
MDC004356.237:6421-6708	AT5G18150.1		PTHR12133				expressed protein
MDC004356.237:6808-6910	AT5G59970.1	PF00125	PTHR10484	KOG3467		K11254	Core histone H2A/H2B/H3/H4 domain containing protein, putative, expressed
MDC004389.393:4277-4752	AT5G04500.1	PF09258	PTHR11062	KOG1022			exostosin, putative, expressed
MDC004425.303:2507-3293	AT2G46150.1	PF03168					harpin-induced protein 1 domain containing protein, expressed
MDC004475.200:4627-5729	AT3G12870.1						expressed protein
MDC004559.262:978-1347	AT3G25940.1	PF01096	PTHR11239	KOG2907	2.7.7.6	K03000	DNA-directed RNA polymerase I subunit RPA12, putative, expressed
MDC004588.262:3095-3346	AT5G53070.1	PF01281	PTHR21368	KOG4607			ribosomal L9, putative, expressed
MDC004652.210:27146-27473	AT1G11910.1	PF00026,PF03489,PF05184	PTHR13683	KOG1339	3.4.23.40	K08245	aspartic proteinase oryzasin-1 precursor, putative, expressed
MDC004719.334:33353-33870	AT1G11910.1	PF00026,PF03489,PF05184	PTHR13683	KOG1339	3.4.23.40	K08245	aspartic proteinase oryzasin-1 precursor, putative, expressed
MDC004719.334:35584-35741	AT1G02070.1						zinc finger protein, putative, expressed
MDC004742.548:8028-8154	AT3G18215.1	PF04654					expressed protein
MDC004763.566:3821-4013	AT5G18790.1	PF00471		KOG3505			50S ribosomal protein L33, putative, expressed
MDC004790.466:18774-19119	AT1G05960.2		PTHR12444	KOG1877			cyclin-related protein, putative, expressed
MDC004812.214:10140-10649	AT2G46590.2	PF02701					dof zinc finger domain containing protein, putative, expressed
MDC004894.273:6281-6551	AT4G30380.1	PF03330					beta-expansin precursor, putative, expressed
MDC004903.290:28-253	AT4G35783.1	PF08137					conserved hypothetical protein
MDC005044.363:2733-3247	AT4G25260.1	PF04043					invertase/pectin methylesterase inhibitor family protein, putative, expressed
MDC005053.110:4914-5359	AT5G62360.1	PF04043					invertase/pectin methylesterase inhibitor family protein, putative, expressed
MDC005053.110:5464-5590	AT4G23100.3	PF04107			6.3.2.2	K01919	glutamate--cysteine ligase, chloroplast precursor, putative, expressed
MDC005075.307:3581-3994	AT2G29590.1	PF03061	PTHR21660	KOG3328			thioesterase family protein, putative, expressed

MDC005094.389:2512-3049	AT2G06990.1	PF00270,PF00271,PF08148	PTHR11752	KOG0948			DSHCT domain containing protein, expressed
MDC005141.258:7613-7859	AT5G49810.1		PTHR11751	KOG0257	2.1.1.12	K08247	methionine S-methyltransferase, putative, expressed
MDC005190.583:11765-12203	AT5G20170.1						expressed protein
MDC005190.586:28693-29117	AT3G06170.1	PF03348	PTHR10383	KOG2592			TMS membrane protein/tumour differentially expressed protein, putative, expressed
MDC005242.172:12540-13598	AT5G66930.3	PF07855	PTHR13292	KOG4493			expressed protein
MDC005257.231:6275-6378	AT5G53370.1	PF04043,PF01095	PTHR22931:SF5				pectinesterase, putative, expressed
MDC005334.309:40809-41221	AT1G17020.1	PF03171	PTHR10209	KOG0143			naringenin,2-oxoglutarate 3-dioxygenase, putative, expressed
MDC000612.338:24965-25404	AT4G17190.1	PF00348	PTHR11525	KOG0711	2.5.1.1,2.5.1.:	K00787	polyprenyl synthetase, putative, expressed
MDC005353.186:3030-3296	AT1G34750.1	PF00481	PTHR13832	KOG0698			protein phosphatase 2C, putative, expressed
MDC005367.75:16852-17121	AT1G22280.3	PF00481	PTHR13832	KOG0698			protein phosphatase 2C, putative, expressed
MDC005367.75:17253-17433	AT1G61070.1	PF07333,PF00304					DEF8 - Defensin and Defensin-like DEFL family, expressed
MDC005456.101:32525-33076	AT3G14630.1	PF00067	PTHR19383	KOG0157			cytochrome P450 72A1, putative, expressed
MDC005483.475:1862-2359	AT3G14630.1	PF00067	PTHR19383	KOG0157			cytochrome P450 72A1, putative, expressed
MDC005483.475:2519-2878	AT5G56170.1						GPI-anchored protein, putative, expressed
MDC005607.185:3202-3687	AT4G30790.1	PF10377	PTHR13222				expressed protein
MDC000651.338:6690-7035	AT3G03520.1	PF04185			3.1.4.3	K01114	phosphoesterase family protein, putative, expressed
MDC005623.471:5146-5546	AT1G13440.2	PF00044,PF02800	PTHR10836	KOG0657	1.2.1.12	K00134	glyceraldehyde-3-phosphate dehydrogenase, putative, expressed
MDC005648.396:4843-5646	AT5G64610.1	PF11717,PF00385,PF01853	PTHR10615	KOG2747	2.3.1.48	K11308	MYST-like histone acetyltransferase 1, putative, expressed
MDC005683.152:7464-7625	AT1G21630.1	PF00036	PTHR11216	KOG0998			EF hand family protein, putative, expressed
MDC005717.683:79-577	AT1G05170.2	PF01762	PTHR11214	KOG2288			galactosyltransferase, putative, expressed
MDC005782.166:27529-28337	AT5G15790.2	PF00097	PTHR22766				zinc finger, C3HC4 type domain containing protein, expressed
MDC005795.287:6123-6431	AT2G39500.1						
MDC005856.445:7345-7621	AT5G51190.1	PF00847					AP2 domain containing protein, expressed
MDC005866.401:35521-36684	AT3G12360.1	PF00023	PTHR18958	KOG4412			ankyrin repeat-containing protein, putative, expressed
MDC005950.209:1841-2991	AT3G12360.1	PF00023	PTHR18958	KOG4412			ankyrin repeat-containing protein, putative, expressed
MDC005950.237:13639-14704	AT1G22070.1	PF00170,PF07716				K14431	transcription factor, putative, expressed
MDC000667.347:25394-25851	AT3G49590.3	PF10033		KOG4573			expressed protein
MDC006166.69:22399-22979	AT1G71865.1						expressed protein
MDC006167.203:6785-7413	AT5G51280.1	PF00270,PF00271,PF00098	PTHR10967	KOG0341	3.6.4.13	K13116	DEAD-box ATP-dependent RNA helicase 35A, putative, expressed
MDC006191.181:59671-60011	AT1G19130.1	PF06172				K09705	cupin superfamily protein, putative, expressed
MDC006200.189:255-378	AT1G19130.1	PF06172				K09705	cupin superfamily protein, putative, expressed
MDC006200.189:719-977	AT3G61770.1	PF02681					Divergent PAP2 family domain containing protein, expressed
MDC006204.278:5342-5722	AT1G33055.1						expressed protein
MDC006212.350:19119-19764	AT1G05810.1	PF00071,PF08477	PTHR11708	KOG0087		K07976	ras-related protein, putative, expressed
MDC006231.909:29746-30018	AT3G28050.1	PF00892					auxin-induced protein 5NG4, putative, expressed
MDC006246.466:8563-8734	AT1G23440.1		PTHR23402	KOG4755			pyrrolidone-carboxylate peptidase, putative, expressed
MDC006260.462:6583-6877	AT2G45960.3	PF00230	PTHR19139	KOG0223		K09872	aquaporin protein, putative, expressed
MDC006289.408:8288-8577	AT4G00430.1	PF00230	PTHR19139	KOG0223		K09872	aquaporin protein, putative, expressed
MDC006289.408:9216-9268	AT4G27750.1						impaired sucrose induction 1, putative, expressed
MDC006289.410:4115-4432	AT2G37750.1						
MDC006340.216:19210-20061	AT1G26670.1	PF05008,PF12352	PTHR21230	KOG1666		K08493	vesicle transport v-SNARE protein, putative, expressed
MDC006363.247:28792-29092	AT5G49525.1						expressed protein
MDC006424.194:9322-9729	AT1G67620.1	PF02410	PTHR21043	KOG3212			expressed protein
MDC006471.130:167-359	AT4G14103.2	PF00646,PF07723					OsFBL3 - F-box domain and LRR containing protein, expressed
MDC006517.413:12768-13371	AT1G65000.1						expressed protein
MDC006520.239:5991-6735	AT2G31085.1						
MDC006520.258:40616-41227	AT2G26740.1	PF12146,PF00561	PTHR10992	KOG4178			hydrolase, alpha/beta fold family domain containing protein, expressed
MDC006603.356:888-1307	AT3G04180.1	PF00190,PF07883					Cupin domain containing protein, expressed
MDC006603.734:7137-7471	AT1G67830.1	PF00657					GDSL-like lipase/acylhydrolase, putative, expressed
MDC006603.753:648-986	AT5G59960.1						expressed protein
MDC006624.226:615-886	AT5G59970.1	PF00125	PTHR10484	KOG3467		K11254	Core histone H2A/H2B/H3/H4 domain containing protein, putative, expressed
MDC006624.226:6675-7194	AT3G19760.1	PF00270,PF00271	PTHR10967	KOG0328	3.6.4.13	K13025	DEAD-box ATP-dependent RNA helicase, putative, expressed
MDC006639.149:9374-9510	AT3G19760.1	PF00270,PF00271	PTHR10967	KOG0328	3.6.4.13	K13025	DEAD-box ATP-dependent RNA helicase, putative, expressed
MDC006639.149:10169-10560	AT3G13530.1	PF00069,PF07714,PF00514,PF02985	PTHR22986	KOG0198			STE_MEKK_ste11_MAP3K.1 - STE kinases include homologs to sterile 7, sterile 11 and sterile 20 from yeast, expressed
MDC006639.149:10658-11039	AT4G17560.1	PF01245	PTHR15680	KOG1698			50S ribosomal protein L19, chloroplast precursor, putative, expressed
MDC006675.305:292-384	AT5G47190.1	PF01245	PTHR15680	KOG1698			50S ribosomal protein L19, chloroplast precursor, putative, expressed
MDC006675.305:1240-1535	AT3G54820.1	PF00230	PTHR19139	KOG0223		K09872	aquaporin protein, putative, expressed
MDC006710.363:6328-6707	AT3G54820.1	PF00230	PTHR19139	KOG0223		K09872	aquaporin protein, putative, expressed
MDC006710.363:6868-6988	AT2G37170.1	PF00230	PTHR19139	KOG0223		K09872	aquaporin protein, putative, expressed
MDC006710.363:8904-9171	AT3G54820.1	PF00230	PTHR19139	KOG0223		K09872	aquaporin protein, putative, expressed
MDC006710.363:9415-9810	AT5G09960.1	PF05627					expressed protein
MDC006772.187:518-757	AT5G51190.1	PF00847					AP2 domain containing protein, expressed
MDC000706.497:11725-12685	AT2G28840.2	PF00023	PTHR18958	KOG4214			ankyrin repeat domain-containing protein 28, putative, expressed
MDC006804.119:728-1335	AT1G08230.2	PF01490	PTHR22950	KOG1303			transmembrane amino acid transporter protein, putative, expressed
MDC006834.195:5931-6204	AT1G09900.1	PF01535	PTHR10483				pentatricopeptide repeat domain containing protein, putative, expressed
MDC007047.426:19297-19639	AT5G47820.2	PF00225	PTHR16012	KOG0244		K10395	kinesin motor domain containing protein, putative, expressed
MDC007057.148:1220-1518	AT4G22570.1	PF00156		KOG1712	2.4.2.7	K00759	phosphoribosyl transferase, putative, expressed
MDC007058.290:9956-10075	AT5G16380.1	PF04398					expressed protein
MDC007066.597:3722-4200	AT2G39970.1	PF00153	PTHR11896	KOG0769		K13354	mitochondrial carrier protein, putative, expressed
MDC007117.619:30844-31444	AT5G23070.1	PF00265	PTHR11441	KOG3125	2.7.1.21	K00857	thymidine kinase, putative, expressed
MDC007192.252:1298-1688	AT3G06980.1	PF00270,PF00271	PTHR10967	KOG0331			DEAD-box ATP-dependent RNA helicase, putative, expressed
MDC007239.507:18593-18889	AT1G74340.1	PF07297		KOG3488		K09658	dolichol phosphate-mannose biosynthesis regulatory protein, putative, expressed
MDC000741.237:17856-18144	AT5G19140.2	PF12481,PF12504	PTHR11772				stem-specific protein TSJT1, putative, expressed
MDC007283.1041:967-1726	AT3G57080.1	PF03871,PF01191	PTHR10535	KOG3218			DNA-directed RNA polymerases I, II, and III subunit RPABC1, putative, expressed
MDC007283.917:10694-10994	AT1G56070.1	PF00009,PF01926,PF03144,PF03764,PF00679	PTHR23115	KOG0469	3.6.5.3	K03234	elongation factor, putative, expressed
MDC007289.107:9052-9887	AT5G02490.1	PF00012	PTHR19375	KOG0100		K03283	DnaK family protein, putative, expressed
MDC007308.338:25153-25355	AT5G35910.1	PF01612,PF00570	PTHR12124	KOG2206	3.1.13.-	K12591	3-5 exonuclease family protein, putative, expressed
MDC007315.261:46384-46703	AT5G61670.2						OR, putative, expressed
MDC007344.1025:7310-7666	AT4G25620.1						expressed protein
MDC000796.524:10939-12475	AT4G09550.1	PF12554					expressed protein

MDC007350.839:8348-8675	AT2G28680.1	PF00190					cupin domain containing protein, expressed
MDC007376.494:17698-18125	AT2G28680.1	PF00190					cupin domain containing protein, expressed
MDC007376.494:18463-18905	AT1G07750.1	PF00190					cupin domain containing protein, expressed
MDC007376.494:18965-19412	AT3G12700.1		PTHR13683	KOG1339			aspartyl protease family protein, putative, expressed
MDC007422.177:2747-3819	AT3G12700.2		PTHR13683				aspartyl protease family protein, putative, expressed
MDC007422.177:4802-5629	AT5G11760.1	PF08576					expressed protein
MDC007454.249:3101-3502	AT1G75620.1	PF07250,PF09118					glyoxal oxidase-related, putative, expressed
MDC007469.326:38450-38743	AT5G62000.4	PF02362,PF06507,PF02309					auxin response factor, putative, expressed
MDC007485.683:834-1172	AT2G43370.1	PF00076	PTHR13952	KOG0113		K13155	RNA recognition motif containing protein, putative, expressed
MDC007509.254:671-866	AT5G41800.1	PF01490	PTHR22950	KOG1303			transmembrane amino acid transporter protein, putative, expressed
MDC000812.292:23436-23602	AT4G22720.2	PF00814	PTHR11735	KOG2708	3.4.24.57	K01409	O-sialoglycoprotein endopeptidase, putative, expressed
MDC007568.248:14938-15506	AT4G22720.2	PF00814	PTHR11735	KOG2708	3.4.24.57	K01409	O-sialoglycoprotein endopeptidase, putative, expressed
MDC007568.248:16771-17120	AT2G40470.1	PF03195					DUF260 domain containing protein, putative, expressed
MDC007582.375:15700-16040	AT4G19185.1	PF00892					nodulin, putative, expressed
MDC007598.175:7142-7412	AT4G19185.1	PF00892					nodulin, putative, expressed
MDC007598.175:13744-13944	AT1G31500.4	PF03372	PTHR12121	KOG0620			endonuclease/exonuclease/phosphatase family domain containing protein, expressed
MDC007614.448:8696-9035	AT3G23600.2	PF01738	PTHR17630	KOG3043			endo-1,3;1,4-beta-D-glucanase precursor, putative, expressed
MDC007624.239:4383-4761	AT5G41800.1	PF01490	PTHR22950	KOG1303			transmembrane amino acid transporter protein, putative, expressed
MDC000812.292:23731-24364	AT2G01480.1	PF10250					growth regulator related protein, putative
MDC007641.311:4854-5781	AT4G32300.1	PF01453,PF00069,PF07714	PTHR23258	KOG1187			lectin protein kinase family protein, putative, expressed
MDC007651.256:7095-7497	AT2G18030.1	PF01625	PTHR10173	KOG1635			peptide methionine sulfoxide reductase, putative, expressed
MDC007657.717:4681-5123	AT5G42960.1						DANA2, putative, expressed
MDC007713.205:2812-3135	AT4G13195.1						
MDC007725.313:21618-22339	AT1G11170.1	PF05212					lysine ketoglutarate reductase trans-splicing related 1, putative, expressed
MDC007736.142:17091-17712	AT4G30770.1						expressed protein
MDC000813.771:2702-3359	AT1G74530.3						expressed protein
MDC007790.237:180-282	AT2G02090.1	PF00176,PF00271	PTHR10799	KOG0389	3.6.4.12	K14439	SNF2 family N-terminal domain containing protein, expressed
MDC007791.237:10017-10398	AT1G76900.2	PF00646,PF01167	PTHR16517	KOG2502			OsFBT7 - F-box and tubby domain containing protein, expressed
MDC007792.401:10562-11508	AT2G33730.1	PF00270,PF00271	PTHR10967	KOG0333	3.6.4.13	K12858	DEAD-box ATP-dependent RNA helicase, putative, expressed
MDC007792.401:29323-29673	AT4G08770.1	PF00141			1.11.1.7	K00430	peroxidase precursor, putative, expressed
MDC007858.422:10539-10754	AT5G05340.1	PF00141			1.11.1.7	K00430	peroxidase precursor, putative, expressed
MDC007858.422:12370-12964	AT2G47900.3	PF00646,PF01167	PTHR16517	KOG2502			OsFBT5 - F-box and tubby domain containing protein, expressed
MDC007953.177:16669-16974	AT1G12410.1	PF00574	PTHR10381	KOG0840	3.4.21.92	K01358	OsClp9 - Putative Clp protease homologue, expressed
MDC008045.226:5778-6105	AT3G61430.2	PF00230	PTHR19139	KOG0223		K09872	aquaporin protein, putative, expressed
MDC008099.122:14997-15532	AT4G23400.1	PF00230	PTHR19139	KOG0223		K09872	aquaporin protein, putative, expressed
MDC008099.122:15720-15853	AT4G00300.2	PF11721					receptor-like protein kinase At3g46290 precursor, putative, expressed
MDC000814.336:7471-7949	AT1G01620.2	PF00230	PTHR19139	KOG0223		K09872	aquaporin protein, putative, expressed
MDC008099.122:15946-16282	AT4G00430.1	PF00230	PTHR19139	KOG0223		K09872	aquaporin protein, putative, expressed
MDC008099.122:16388-16809	AT5G40240.2	PF00892		KOG1441			auxin-induced protein 5NG4, putative, expressed
MDC008104.97:4350-4465	AT5G40230.1	PF00892					auxin-induced protein 5NG4, putative, expressed
MDC008104.97:4893-5080	AT3G17490.1	PF00646,PF07734					OsFBX464 - F-box domain containing protein
MDC008108.99:7464-9223	AT2G34340.1	PF04520					DUF584 domain containing protein, putative, expressed
MDC008119.473:1280-1805	AT2G24100.1						expressed protein
MDC008177.674:13219-13664	AT4G12710.1	PF00514	PTHR23316	KOG4646			armadillo/beta-catenin repeat family protein, putative, expressed
MDC008184.205:8772-9239	AT1G54540.1	PF03168					harpin-induced protein 1 domain containing protein, expressed
MDC008212.498:491-1195	AT1G69410.1	PF00467,PF01287	PTHR11673	KOG3271		K03263	eukaryotic translation initiation factor 5A, putative, expressed
MDC008233.164:1510-1669	AT4G02420.1	PF00139,PF00069,PF07714	PTHR23258	KOG1187			receptor like protein kinase, putative, expressed
MDC008313.318:4207-4571	AT1G74530.3						expressed protein
MDC008325.354:8874-8959	AT5G17760.1	PF00004	PTHR23070	KOG0743			mitochondrial chaperone BCS1, putative, expressed
MDC008336.464:6231-7078	AT4G28210.1						
MDC008345.348:4511-4874	AT5G47470.1	PF00892					integral membrane protein DUF6 domain containing protein, expressed
MDC008453.913:10123-10374	AT4G21800.2	PF03029	PTHR21231	KOG1532		K06883	ATP binding protein, putative, expressed
MDC008485.183:6542-7083	AT5G42965.1						conserved hypothetical protein
MDC008496.393:3311-4214	AT5G47180.2	PF00635	PTHR10809	KOG0439			MSP domain containing protein, expressed
MDC008501.186:4780-5080	AT2G24100.1						expressed protein
MDC008511.144:10693-11189	AT4G10360.2	PF03798	PTHR13439	KOG4561			transmembrane protein 56, putative, expressed
MDC008521.354:1690-2152	AT1G06650.2	PF03171	PTHR10209	KOG0143			1-aminocyclopropane-1-carboxylate oxidase homolog 2, putative, expressed
MDC000835.215:2033-2459	AT1G30970.2		PTHR23215	KOG2893			ZOS9-19 - C2H2 zinc finger protein, expressed
MDC008558.180:22776-23209	AT5G27770.1	PF01776	PTHR10064	KOG3434		K02891	60S ribosomal protein L22-2, putative, expressed
MDC008575.351:1527-1902	AT5G27770.1	PF01776	PTHR10064	KOG3434		K02891	60S ribosomal protein L22-2, putative, expressed
MDC008575.351:2734-2941	AT5G41330.1	PF02214	PTHR11145	KOG2714			B4-BTB4 - Bric-a-Brac, Tramtrack, Broad Complex BTB domain with B4 subfamily conserved sequence, expressed
MDC008579.487:3510-3869	AT1G71950.1	PF05922	PTHR10795				subtilisin N-terminal Region family protein, expressed
MDC008657.482:3281-3527	AT4G12020.3	PF02671,PF03106,PF00931,PF07725,PF00560,PF00069,PF07714	PTHR22986	KOG0198			STE_MEKK_ste11_MAP3K.12 - STE kinases include homologs to sterile 7, sterile 11 and sterile 20 from yeast, expressed
MDC008801.230:1128-1609	AT1G07510.1	PF06480,PF00004,PF01434	PTHR23076	KOG0731	3.4.24.-	K08956	OsFtsH8 FtsH protease, homologue of AtFtsH3/10, expressed
MDC008815.152:3297-3548	AT1G50670.1	PF02338	PTHR13312		3.1.2.-	K13719	OTU-like cysteine protease family protein, putative, expressed
MDC008844.652:13597-13959	AT1G20430.1		PTHR21568				expressed protein
MDC008880.141:64155-64719	AT2G36985.1	PF08137					expressed protein
MDC008909.172:124-491	AT1G20980.1	PF03110					OsSPL15 - SBP-box gene family member, expressed
MDC008927.255:16667-17470	AT5G66780.1						expressed protein
MDC008947.382:12415-12822	AT1G30760.1	PF01565,PF08031	PTHR11748				reticuline oxidase-like protein precursor, putative, expressed
MDC000845.305:5283-5857	AT1G11910.1	PF00026,PF03489,PF05184	PTHR13683	KOG1339	3.4.23.40	K08245	aspartic proteinase oryzasin-1 precursor, putative, expressed
MDC008968.446:15847-16400	AT4G31750.1	PF00481	PTHR13832	KOG0698			protein phosphatase 2C, putative, expressed
MDC009079.262:4671-4942	AT4G31750.1	PF00481	PTHR13832	KOG0698			protein phosphatase 2C, putative, expressed
MDC009079.262:6115-6244	AT4G31750.1	PF00481	PTHR13832	KOG0698			protein phosphatase 2C, putative, expressed
MDC009079.262:7239-7401	AT1G25510.1		PTHR13683	KOG1339			aspartic proteinase, putative, expressed
MDC000851.382:15529-16034	AT5G40230.1	PF00892					auxin-induced protein 5NG4, putative, expressed
MDC009088.260:67-301	AT3G28050.1	PF00892					auxin-induced protein 5NG4, putative, expressed
MDC009088.260:680-830	AT3G28050.1	PF00892					auxin-induced protein 5NG4, putative, expressed
MDC009088.260:987-1845	AT4G37270.1	PF00122,PF00702	PTHR11939	KOG0207			cadmium/zinc-transporting ATPase, putative, expressed
MDC009100.743:18942-19318	AT5G58950.1	PF07714,PF00069	PTHR23257	KOG0192			protein kinase domain containing protein, expressed

MDC009128.602:32330-32740	AT2G23090.1	PF04419					expressed protein
MDC009154.309:1280-1387	AT3G60210.1	PF00166	PTHR10772	KOG1641			chaperonin, putative, expressed
MDC009197.269:132-321	AT1G24260.3	PF00319,PF01486	PTHR11945	KOG0014			OsMADS7 - MADS-box family gene with MIKCc type-box, expressed
MDC009276.331:6201-6936	AT1G58120.1						CPuORF38 - conserved peptide uORF-containing transcript, expressed
MDC009282.275:3906-4729	AT1G16900.1	PF03901	PTHR22760	KOG2515	2.4.1.-	K03846	Alg9-like mannosyltransferase protein, putative, expressed
MDC009291.126:1104-1457	AT5G59970.1	PF00125	PTHR10484	KOG3467		K11254	Core histone H2A/H2B/H3/H4 domain containing protein, putative, expressed
MDC009303.382:1557-1878	AT1G51060.1	PF00125,PF00808	PTHR23430	KOG1756		K11251	core histone H2A/H2B/H3/H4, putative, expressed
MDC000876.364:2401-2783	AT5G38200.1	PF07722					
MDC009325.280:310-749	AT2G39730.1	PF00004		KOG0651			AAA-type ATPase family protein, putative, expressed
MDC009368.188:2702-3008	AT5G36930.2	PF01582,PF00560,PF00931,PF07725	PTHR23155	KOG4658			disease resistance protein RGA3, putative, expressed
MDC009423.544:1637-2373	AT2G17420.1	PF07992,PF00070	PTHR22912	KOG0404	1.8.1.9	K00384	thioredoxin reductase 2, putative, expressed
MDC009436.50:32007-32411	AT3G45140.1	PF01477,PF00305	PTHR11771		1.13.11.12	K00454	lipoxygenase, putative, expressed
MDC009458.215:14228-14402	AT5G56670.1	PF04758	PTHR12650	KOG0009		K02983	expressed protein
MDC009483.38:3322-3461	AT5G47310.1	PF05903	PTHR12378	KOG0324			ethylene-responsive element-binding protein, putative, expressed
MDC009489.259:582-956	AT5G47310.1	PF05903	PTHR12378	KOG0324			ethylene-responsive element-binding protein, putative, expressed
MDC009489.259:2379-2689	AT5G47310.1	PF05903	PTHR12378	KOG0324			ethylene-responsive element-binding protein, putative, expressed
MDC009489.375:2207-2531	AT2G23570.1						OsPOP4 - Putative Prolyl Oligopeptidase homologue, expressed
MDC009490.558:6365-6741	AT2G31600.1						expressed protein
MDC009501.247:2219-2729	AT4G16143.2	PF01749,PF00514,PF02985,PF03130	PTHR23316	KOG0166			importin subunit alpha, putative, expressed
MDC009595.146:45-416	AT3G14430.1						expressed protein
MDC009596.264:70-344	AT2G20290.1	PF02736,PF00063,PF00612,PF01843	PTHR13140	KOG0160			myosin, putative, expressed
MDC009602.113:10106-10498	AT1G71950.1	PF05922	PTHR10795				subtilisin N-terminal Region family protein, expressed
MDC009615.292:631-756	AT4G38790.1	PF00810	PTHR10585	KOG3106			ER lumen protein retaining receptor, putative, expressed
MDC009626.195:1302-1624	AT5G41760.2	PF04142	PTHR10231	KOG2234			UAA transporter family domain containing protein, expressed
MDC000140.134:54551-54847	AT5G10180.1	PF00916,PF01740	PTHR11814	KOG0236			sulfate transporter, putative, expressed
MDC009685.334:6702-7096	AT1G75460.1	PF02190	PTHR23327	KOG4159			ATP-dependent protease, putative, expressed
MDC009708.152:51041-51699	AT5G40150.1	PF00141			1.11.1.7	K00430	peroxidase family protein, expressed
MDC000908.450:4034-4471	AT3G04730.1	PF02309					OsIAA30 - Auxin-responsive Aux/IAA gene family member, expressed
MDC009814.418:6273-6657	AT4G17690.1	PF00141			1.11.1.7	K00430	peroxidase family protein, expressed
MDC000908.450:4523-5057	AT4G14550.1	PF02309					OsIAA30 - Auxin-responsive Aux/IAA gene family member, expressed
MDC009814.418:7581-7800	AT3G28430.1	PF09758	PTHR21481	KOG2219			expressed protein
MDC009830.368:4257-4492	AT5G65780.1	PF01063	PTHR11825	KOG0975	2.6.1.42	K00826	aminotransferase domain containing protein, putative, expressed
MDC009850.21:558-987	AT1G19580.1	PF00132	PTHR22572	KOG4042			bacterial transferase hexapeptide domain containing protein, expressed
MDC009856.139:35298-35913	AT1G06990.1	PF00657	PTHR22835:SF27				GDSL-like lipase/acylhydrolase, putative, expressed
MDC009859.245:7701-7947	AT1G16180.2	PF03348	PTHR10383	KOG2592			TMS membrane protein/tumour differentially expressed protein, putative, expressed
MDC009907.294:23684-23868	AT1G16180.2	PF03348	PTHR10383	KOG2592			TMS membrane protein/tumour differentially expressed protein, putative, expressed
MDC009907.294:24320-24561	AT1G16180.2	PF03348	PTHR10383	KOG2592			TMS membrane protein/tumour differentially expressed protein, putative, expressed
MDC009907.294:24876-25099	AT4G34700.1	PF05347	PTHR12868	KOG3466	1.6.5.3,1.6.99	K03965	LYR motif containing protein, putative, expressed
MDC009946.417:4427-4703	AT5G37600.1	PF03951,PF00120	PTHR20852	KOG0683	6.3.1.2	K01915	glutamine synthetase, catalytic domain containing protein, expressed
MDC009950.283:2248-2601	AT1G66200.3	PF03951,PF00120	PTHR20852	KOG0683			glutamine synthetase, catalytic domain containing protein, expressed
MDC009950.283:3474-3584	AT5G37600.1	PF03951,PF00120	PTHR20852	KOG0683	6.3.1.2	K01915	glutamine synthetase, catalytic domain containing protein, expressed
MDC009950.283:3884-4023	AT5G37600.1	PF03951,PF00120	PTHR20852	KOG0683	6.3.1.2	K01915	glutamine synthetase, catalytic domain containing protein, expressed
MDC009950.283:4266-4401	AT5G16570.1	PF03951,PF00120	PTHR20852	KOG0683	6.3.1.2	K01915	glutamine synthetase, catalytic domain containing protein, expressed
MDC009950.288:5596-6266	AT5G32470.1	PF03070	PTHR20858				TENA/THI-4 family protein, putative, expressed
MDC009976.359:12472-13055	AT5G32470.1	PF03070	PTHR20858				TENA/THI-4 family protein, putative, expressed
MDC009976.359:13136-13571	AT2G36070.1	PF04280	PTHR10721	KOG2580			tim44-like domain containing protein, expressed
MDC009991.166:1671-1911	AT4G23160.1	PF07727,PF01657,PF00069,PF07714,PF11883	PTHR11439	KOG1187			TKL_IRAK_DUF26-lc.20 - DUF26 kinases have homology to DUF26 containing loci, expressed
MDC009992.373:5233-5529	AT5G02230.2	PF00702	PTHR18901	KOG3109			haloacid dehalogenase-like hydrolase family protein, putative, expressed
MDC010058.303:36260-36547	AT5G41800.1	PF01490	PTHR22950	KOG1303			transmembrane amino acid transporter protein, putative, expressed
MDC010065.349:3326-3668	AT2G22640.1					K05752	BRICK1, putative, expressed
MDC010071.397:392-525	AT1G56700.3		PTHR23402	KOG4755			pyrrolidone-carboxylate peptidase, putative, expressed
MDC000997.260:16008-16410	AT4G16450.2						expressed protein
MDC010085.172:13-504	AT3G10050.1	PF00291,PF00585	PTHR10314	KOG1250	4.3.1.19	K01754	threonine dehydratase biosynthetic, chloroplast precursor, putative, expressed
MDC010099.229:4737-5125	AT5G06700.1	PF03005					leaf senescence related protein, putative, expressed
MDC010104.1012:30864-31577	AT2G32000.2	PF01751,PF01131	PTHR11390	KOG1957	5.99.1.2	K03165	DNA topoisomerase 3 protein, putative, expressed
MDC010172.365:23895-24342	AT2G37170.1	PF00230	PTHR19139	KOG0223		K09872	aquaporin protein, putative, expressed
MDC010185.231:6884-7177	AT1G56700.3		PTHR23402	KOG4755			pyrrolidone-carboxylate peptidase, putative, expressed
MDC000997.260:17123-17228	AT3G50590.1	PF00400	PTHR12816				WD domain, G-beta repeat domain containing protein, expressed
MDC010186.403:23504-23954	AT5G26800.1	PF09597					expressed protein
MDC010192.333:5522-5655	AT3G12360.1	PF00023	PTHR18958	KOG4412			ankyrin repeat-containing protein, putative, expressed
MDC010192.348:12503-13571	AT5G62790.2	PF02670,PF08436			1.1.1.267	K00099	1-deoxy-D-xylulose 5-phosphate reductoisomerase, chloroplast precursor, putative, expressed
MDC010200.476:4261-4691	AT5G62790.2	PF02670,PF08436			1.1.1.267	K00099	1-deoxy-D-xylulose 5-phosphate reductoisomerase, chloroplast precursor, putative, expressed
MDC010200.476:4901-5052	AT5G51970.2	PF08240,PF00107	PTHR11695	KOG0024	1.1.1.14	K00008	dehydrogenase, putative, expressed
MDC010241.223:17213-18085	AT1G23440.1		PTHR23402	KOG4755			pyrrolidone-carboxylate peptidase, putative, expressed
MDC000997.260:17440-17768	AT5G40350.1	PF00249	PTHR10641	KOG0048		K09422	MYB family transcription factor, putative
MDC010252.317:1021-1375	AT5G64360.4	PF00226	PTHR11821				heat shock protein DnaJ, putative, expressed
MDC010254.244:14459-14910	AT3G56150.2	PF05470,PF01399	PTHR13937	KOG1076		K03252	eukaryotic translation initiation factor 3 subunit C, putative, expressed
MDC010268.425:12245-12627	AT3G42860.1	PF06839,PF00098	PTHR23002	KOG4400			GRF zinc finger family protein, expressed
MDC010300.238:15044-16078	AT3G55640.1	PF00153	PTHR11896	KOG0752			mitochondrial carrier protein, putative, expressed
MDC010318.100:9492-9884	AT3G19080.1	PF08766,PF02201	PTHR13844	KOG1946			upstream activation factor subunit spp27, putative, expressed
MDC010319.227:8072-8321	AT1G49820.1	PF01636					methylthioribose kinase, putative, expressed
MDC010325.233:4683-4903	AT1G48300.1						expressed protein
MDC010328.213:18449-19160	AT1G62290.2	PF00026,PF03489,PF05184	PTHR13683	KOG1339	3.4.23.40	K08245	aspartic proteinase oryzasin-1 precursor, putative, expressed
MDC010328.215:28204-28446	AT3G46890.1						expressed protein
MDC010431.261:2731-2961	AT2G19080.1		PTHR12289	KOG3028			expressed protein
MDC010441.401:17991-18191	AT5G49525.1						expressed protein
MDC010450.908:6750-7226	AT1G08530.1						expressed protein
MDC010450.949:3543-4321	AT5G45970.1	PF00071,PF08477	PTHR11708	KOG0393		K07975	ras-related protein, putative, expressed
MDC001007.507:15632-16109	AT2G02130.1	PF07333,PF00304					DEF8 - Defensin and Defensin-like DEFL family, expressed
MDC010466.196:24-204	AT5G56170.1						GPI-anchored protein, putative, expressed

MDC010492.245:666-1340	AT5G36110.1	PF00067	PTHR19383	KOG0157			cytochrome P450, putative, expressed
MDC010598.348:9370-9523	AT1G32340.1	PF05773,PF00097,PF01485	PTHR11685	KOG1814	6.3.2.19	K11971	ara54-like RING finger protein, putative, expressed
MDC010640.116:5178-5493	AT4G12760.1						expressed protein
MDC010687.251:5882-6163	AT4G21810.1	PF04511	PTHR11009	KOG0858			Der1-like family domain containing protein, expressed
MDC010697.307:4965-5070	AT4G35790.1	PF00168,PF00614,PF12357	PTHR18896	KOG1329			phospholipase D, putative, expressed
MDC010709.414:585-1037	AT1G02090.3	PF01399	PTHR15350	KOG3250		K12180	proteasome subunit, putative, expressed
MDC010751.335:5076-5282	AT5G27870.1	PF04043,PF01095,PF04886					pectinesterase, putative, expressed
MDC010779.408:15723-16139	AT3G61060.1	PF00646					OsFBX391 - F-box domain containing protein, expressed
MDC010783.139:10332-11071	AT3G61060.1	PF00646					OsFBX391 - F-box domain containing protein, expressed
MDC010783.139:11498-12068	AT1G49700.2	PF09713					plant-specific domain TIGR01589 family protein, putative, expressed
MDC010817.271:27525-27737	AT3G15360.1	PF00085	PTHR10438	KOG0910			thioredoxin, putative, expressed
MDC010842.294:4654-4752	AT1G15520.1	PF00005,PF01061,PF08370	PTHR19241	KOG0065			pleiotropic drug resistance protein, putative, expressed
MDC010937.194:12046-12338	AT5G66900.1	PF05659,PF00931,PF00560	PTHR23155	KOG4658			disease resistance protein, putative, expressed
MDC010969.378:7112-8073	AT3G51430.2	PF03088	PTHR10426	KOG1520			strictosidine synthase, putative, expressed
MDC001040.307:555-1041	AT4G14960.2	PF00091,PF03953	PTHR11588	KOG1376		K07374	tubulin/FtsZ domain containing protein, putative, expressed
MDC001048.310:7356-8038	AT3G15630.1						expressed protein
MDC011163.587:1351-1663	AT3G51000.1	PF12146,PF00561	PTHR10992	KOG4178			hydrolase, alpha/beta fold family domain containing protein, expressed
MDC011167.337:32-262	AT5G48100.1	PF07732,PF00394,PF07731	PTHR11709	KOG1263			laccase precursor protein, putative, expressed
MDC011283.393:3854-4077	AT3G21610.2	PF02681					Divergent PAP2 family domain containing protein, expressed
MDC011325.298:41258-41415	AT3G51240.1	PF03171	PTHR10209	KOG0143	1.14.11.9	K00475	naringenin,2-oxoglutarate 3-dioxygenase, putative, expressed
MDC011373.244:3946-4433	AT3G51240.2	PF03171	PTHR10209	KOG0143	1.14.11.9	K00475	naringenin,2-oxoglutarate 3-dioxygenase, putative, expressed
MDC011373.244:4627-5027	AT3G51240.2	PF03171	PTHR10209	KOG0143	1.14.11.9	K00475	naringenin,2-oxoglutarate 3-dioxygenase, putative, expressed
MDC011373.244:5822-6338	AT4G09830.1						holocarboxylase synthetase, putative, expressed
MDC011377.138:14053-14246	AT4G09830.1						holocarboxylase synthetase, putative, expressed
MDC011377.138:16729-17502	AT1G22790.2						expressed protein
MDC011377.138:23384-24008	AT1G34000.1						high light inducible protein, putative, expressed
MDC011377.138:30623-31125	AT1G34000.1						high light inducible protein, putative, expressed
MDC011377.138:31736-32194	AT1G72730.1	PF00270,PF00271	PTHR10967	KOG0327		K03257	DEAD-box ATP-dependent RNA helicase, putative, expressed
MDC011423.269:13482-14391	AT1G54270.2	PF00270,PF00271	PTHR10967	KOG0327		K03257	DEAD-box ATP-dependent RNA helicase, putative, expressed
MDC011423.269:14495-14923	AT5G20350.1	PF00023,PF01529	PTHR22883	KOG0509			palmitoyltransferase TIP1, putative, expressed
MDC011475.283:13870-14453	AT1G69450.2	PF02714	PTHR13018	KOG1134			DUF221 domain containing protein, expressed
MDC011493.183:13848-14159	AT4G23330.2						
